# Supplementary material for: CMG helicase disassembly is essential and driven by two pathways in budding yeast
Source: EMBO J. 2024 Jul 22;43(18):2. doi: 10.1038/s44318-024-00161-x (PMC11405719; doi:10.1038/s44318-024-00161-x)
Supplement: Supplementary file 1 — Appendix [file 44318_2024_161_MOESM1_ESM.pdf]

Appendix – Table of Contents:

CMG helicase disassembly is essential and mediated by two pathways in budding yeast

Cristian Polo Rivera, Tom D. Deegan and Karim P.M. Labib

Appendix Table S1 Page 1

Appendix Table S2 Page 3

Appendix Table S3 Page 5

Appendix Table S1
Mass spectrometry data showing that CMG and associated replisome components are the major partners of SCF^{Dia2}, largely dependent on the TPR domain of Dia2. Also see Dataset EV1.
This is the first of two independent experiments (the other is in Appendix Table S2).

**Protein**	**Complex**	**Spectral counts (control)**	**Spectral counts (TAP-Sld5)**	**Spectral counts (ProteinA-Dia2)**	**Spectral counts (ProteinA-Dia2-ΔTPR)**
Dia2 (85kDa)	SCF^{Dia2}	85	404	4585	2919
Cdc53 (94kDa)	SCF^{Dia2}	19	351	3008	2938
Skp1 (22kDa)	SCF^{Dia2}	3	85	1802	1440
Hrt1 (14kDa)	SCF^{Dia2}	4	20	134	205
Rub1 (9kDa)	SCF^{Dia2} (NEDD8)	2	17	173	222
Psf1 (24kDa)	CMG (GINS)	15	321	94	11
Psf2 (25kDa)	CMG (GINS)	20	269	66	5
Psf3 (22kDa)	CMG (GINS)	0	194	97	10
Sld5 (34kDa)	CMG (GINS)	2	521	205	24
Cdc45 (74kDa)	CMG	3	551	415	17
Mcm2 (99kDa)	CMG (Mcm2-7)	14	1428	1003	42
Mcm3 (107kDa)	CMG (Mcm2-7)	6	1475	1301	47
Mcm4 (105kDa)	CMG (Mcm2-7)	13	1667	1100	40
Mcm5 (86kDa)	CMG (Mcm2-7)	12	973	670	40
Mcm6 (113kDa)	CMG (Mcm2-7)	13	1550	916	26
Mcm7 (95kDa)	CMG (Mcm2-7)	5	979	794	42
Ctf4 (104kDa)	Replisome	21	2244	2640	68

Tof1 (141kDa)	Replisome (Tof1-Csm3)	4	1224	960	43
Csm3 (36kDa)	Replisome (Tof1-Csm3)	0	140	211	6
Mrc1 (124kDa)	Replisome	2	1483	1225	68
Top1 (90kDa)	Replisome	0	357	181	3
Spt16 (119kDa)	Replisome (FACT)	208	1705	934	217
Pob3 (63kDa)	Replisome (FACT)	78	729	332	92
Pol2 (256kDa)	Pol ε	8	352	100	0
Dpb2 (78kDa)	Pol ε	0	94	39	2
Dpb3 (23kDa)	Pol ε	0	18	14	4
Dpb4 (22kDa)	Pol ε	0	19	7	0
Pol1 (167kDa)	Pol α	3	212	156	35
Pol12 (79kDa)	Pol α	2	72	36	0
Pri1 (48kDa)	Pol α	0	51	26	0
Pri2 (62kDa)	Pol α	0	47	42	0
**Other reported substrates of SCF^{Dia2}**					
Rad51 (51kDa)	-	0	5	17	2
Cdc6	ORC-CDC6	0	0	0	0
Tec1 (55kDa)		0	0	2	0
Sir4 (152kDa)		32	12	62	53

Appendix Table S2
Mass spectrometry data showing that CMG and associated replisome components are the major partners of SCF^{Dia2}, largely dependent on the TPR domain of Dia2. Also see Dataset EV2.
This is the second of two independent experiments (the other is in Appendix Table S1).

**Protein**	**Complex**	**Spectral counts (control)**	**Spectral counts (TAP-Sld5)**	**Spectral counts (ProteinA-Dia2)**	**Spectral counts (ProteinA-Dia2-ΔTPR)**
Dia2 (85kDa)	SCF^{Dia2}	73	515	4917	3775
Cdc53 (94kDa)	SCF^{Dia2}	47	372	2125	2078
Skp1 (22kDa)	SCF^{Dia2}	5	47	1002	998
Hrt1 (14kDa)	SCF^{Dia2}	4	14	160	132
Rub1 (9kDa)	SCF^{Dia2} (NEDD8)	2	24	105	77
Psf1 (24kDa)	CMG (GINS)	0	182	90	2
Psf2 (25kDa)	CMG (GINS)	0	145	46	0
Psf3 (22kDa)	CMG (GINS)	0	60	27	0
Sld5 (34kDa)	CMG (GINS)	0	358	109	0
Cdc45 (74kDa)	CMG	2	502	300	8
Mcm2 (99kDa)	CMG (Mcm2-7)	18	1160	735	5
Mcm3 (107kDa)	CMG (Mcm2-7)	5	1319	790	18
Mcm4 (105kDa)	CMG (Mcm2-7)	9	1353	924	5
Mcm5 (86kDa)	CMG (Mcm2-7)	11	921	545	16
Mcm6 (113kDa)	CMG (Mcm2-7)	4	1303	835	20
Mcm7 (95kDa)	CMG (Mcm2-7)	0	1026	583	13
Ctf4 (104kDa)	Replisome	4	1746	1275	8

Tof1 (141kDa)	Replisome (Tof1-Csm3)	2	1015	645	11
Csm3 (36kDa)	Replisome (Tof1-Csm3)	0	132	103	2
Mrc1 (124kDa)	Replisome	0	1280	736	7
Top1 (90kDa)	Replisome	0	329	140	5
Spt16 (119kDa)	Replisome (FACT)	375	1491	821	260
Pob3 (63kDa)	Replisome (FACT)	95	534	264	88
Pol2 (256kDa)	Pol ε	17	210	114	22
Dpb2 (78kDa)	Pol ε	0	38	34	4
Dpb3 (23kDa)	Pol ε	0	10	10	2
Dpb4 (22kDa)	Pol ε	2	19	12	2
Pol1 (167kDa)	Pol α	5	204	127	19
Pol12 (79kDa)	Pol α	0	58	57	3
Pri1 (48kDa)	Pol α	0	44	39	7
Pri2 (62kDa)	Pol α	0	37	38	4
**Other reported substrates of SCF^{Dia2}**					
Rad51 (51kDa)	-	2	5	13	7
Cdc6	ORC-CDC6	0	0	0	0
Tec1 (55kDa)		0	0	0	3
Sir4 (152kDa)		39	13	45	34

Appendix Table S3

Reagents and resources used in this study.

REAGENT or RESOURCE	SOURCE	IDENTIFIER
**Antibodies**		
Yeast Mcm2 [antigen 1-222; sheep polyclonal]<br>Use 1 in 2,000 for immunoblotting	MRC-PPU Reagents and Services	DU62638
Yeast Mcm3 [antigen 1-222; sheep polyclonal]<br>Use 1 in 1,000 for immunoblotting	MRC-PPU Reagents and Services	N/A
Yeast Mcm4 [antigen 831-933; sheep polyclonal]<br>Use 1 in 2,000 for immunoblotting	MRC-PPU Reagents and Services	DU62578
Yeast Mcm5 [antigen 1-222; sheep polyclonal]<br>Use 1 in 1,000 for immunoblotting	MRC-PPU Reagents and Services	DU62708
Yeast Mcm6 [antigen 801-1017; sheep polyclonal]<br>Use 1 in 2,000 for immunoblotting	MRC-PPU Reagents and Services	DU62612
Yeast Mcm7 [antigen 1-222; sheep polyclonal]<br>Use 1 in 1,000 for immunoblotting	MRC-PPU Reagents and Services	N/A
Yeast Mcm7 [antigen 624-846; sheep polyclonal]<br>Use 1 in 2,000 for immunoblotting	MRC-PPU Reagents and Services	DU56413
Yeast Cdc45 [antigen 1-222; sheep polyclonal]<br>Use 1 in 1,000 for immunoblotting	MRC-PPU Reagents and Services	N/A
Yeast Psf1 [antigen full-length; sheep polyclonal]<br>Use 1 in 3,000 for immunoblotting	MRC-PPU Reagents and Services	DU73858
Yeast Psf2 [antigen full-length; sheep polyclonal]<br>Use 1 in 250 for immunoblotting	MRC-PPU Reagents and Services	DU73962
Yeast Psf3 [antigen full-length; sheep polyclonal]<br>Use 1 in 3,000 for immunoblotting	MRC-PPU Reagents and Services	DU73874
Yeast Sld5 [antigen full-length; sheep polyclonal]<br>Use 1 in 1,000 for immunoblotting	MRC-PPU Reagents and Services	DU73863
Yeast Cdc53 [antigen 1-288; sheep polyclonal]<br>Use 1 in 1,000 for immunoblotting	MRC-PPU Reagents and Services	DU21749

Yeast Sic1 [antigen full-length; sheep polyclonal]<br>Use 1 in 250 for immunoblotting	Santa Cruz Biotechnologies	sc-50441
Peroxidase Anti-Peroxidase Soluble Complex (for immunoblotting against the TAP tag)<br>Use 1 in 5,000 for immunoblotting	Sigma Aldrich	P1291
Anti-sheep IgG HRP [from donkey]<br>Use 1 in 10,000 for immunoblotting	Sigma Aldrich	A3415
Anti-rabbit IgG HRP [from donkey]<br>Use 1 in 10,000 for immunoblotting	Thermo Scientific	A-21207
Anti-FLAG M2 affinity resin	Sigma-Aldrich	A2220-10ML
**Chemicals, Peptides, Recombinant Proteins, other reagents**		
Agar (Bacto Agar)	Becton Dickinson	214010
Amicon Ultra-15 centrifugal Filter Unit, 30kDa	Millipore	UFC903024
Amicon Ultra-15 centrifugal Filter Unit, 100kDa	Millipore	UFC910024
β-glucuronidase	Sigma-Aldrich	G7770
4-12% Bis-Tris NuPAGE	Life Technologies	NP0301
β-mercaptoethanol	Sigma-Aldrich	M6520
Calmodulin affinity resin	Agilent Technologies	214303
DAPI (4', 6-Diamidino-2phenylindole)	Thermo	62248
Dynabeads M-270 Epoxy	Life Technologies	14302D
ECL western blotting detection reagent	VWR	RPN2106
FLAG peptide	Sigma-Aldrich	F4799
Galactose	Formedium	GAL03
Gibson Assembly Cloning Kit	New England Biolabs	E2611
Hydroxyurea	Molekula group	10872383
iBlot membrane	ThermoFisher Scientific	IB301031
IGEPAL CA-630	Sigma-Aldrich	18896
3-Indoleacetic Acid	Sigma-Aldrich	I3750-5G-A
'InstantBlue' colloidal Coomassie blue dye	Abcam	ab119211
4X LDS sample buffer	ThermoFisher Scientific	NP0007
Microscopy slides (VWR SuperFrost Plus, Adhesion Slides)	VWR	631-0108
Magnetic beads (Dynabeads M-270 Epoxy)	Thermo Scientific	14302D
Mating pheromone (α-factor)	Pepceuticals	N/A
Nuclease (Pierce Universal Nuclease)	Fisher Scientific	PN88702

NuPAGE MOPS SDS buffer	ThermoFisher Scientific	NP0001
NuPAGE Tris-Acetate SDS buffer	ThermoFisher Scientific	LA0041
NuPAGE 3–8% Tris-Acetate gels	ThermoFisher Scientific	EA0375BOX & WG1602BOX
NuPAGE Novex 4-12% Bis-Tris gels	ThermoFisher Scientific	NP0321 & WG1402A
Paraformaldehyde, 16% v/v aqueous solution, methanol free	Thermo Scientific	043368.9L
Peptone (Oxoid Peptone Bacteriological)	ThermoFisher Scientific	LP0037B
Pierce Universal Nuclease	ThermoFisher Scientific	88702
Propidium iodide	Sigma-Aldrich	P4170
Protease inhibitor cocktail (Complete Protease Inhibitor Cocktail)	Roche	05056489001
Raffinose	Formedium	RAF04
Roche cOmplete EDTA-free protease inhibitor cocktail	Roche	11873580001
RNase A	ThermoFisher Scientific	EN0531
Simply Blue	ThermoFisher Scientific	LC6060
T4 DNA ligase	New England Biolabs	M202
TEV protease	MRC PPU Reagents and Services	DU6811
Tween 20	Sigma-Aldrich	P1379
Yeast Extract (Bacto Yeast Extract)	Becton Dickinson	212750
Yeast nitrogen base without amino acids	Becton Dickinson	291940
**Experimental Models: Yeast Strains**		
Saccharomyces cerevisiae: YBH295<br>*MAT***a**<br>*RAD52-GFP (TRP1)*	B. Hodgson	N/A
Saccharomyces cerevisiae: YCPR33<br>*MAT***a** / *MAT*α<br>*pep4Δ::kanMX / pep4Δ::kanMX*<br>*bar1Δ::hph-NT / bar1Δ::hphNT*<br>*ura3-1 / ura3-1::URA3(GAL-MCM2 GAL-CBP-TEV-MCM3)*<br>*his3-11 / his3-11::HIS3(GAL-CDC45iFLAG2 GAL-GAL4)*	This study	N/A

trp1-1::TRP1(GAL-PSF1 GAL-SLD5) / trp1-1::TRP1(GAL-MCM5 GAL-MCM4)<br>*leu2-3::LEU2(GAL-PSF2 GAL-PSF3) / leu2-3::LEU2(GAL-mcm7-3TEV(T394) GAL-MCM6)*<br>*ctf4-I901E / ctf4-I901E*		
Saccharomyces cerevisiae: YCPR38<br>*MAT***a** / *MATα*<br>*pep4Δ::kanMX / pep4Δ::kanMX*<br>*bar1Δ::hph-NT / bar1Δ::hphNT*<br>*ura3-1 / ura3-1::URA3(GAL-MCM2 GAL-CBP-TEV-MCM3)*<br>*his3-11 / his3-11::HIS3(GAL-CDC45iFLAG2 GAL-GAL4)*<br>*trp1-1::TRP1(GAL-PSF1 GAL-SLD5) / trp1-1::TRP1(GAL-MCM5 GAL-MCM4)*<br>*leu2-3::LEU2(GAL-PSF2 GAL-PSF3) / leu2-3::LEU2(GAL-mcm7-10R-3TEV(A219) GAL-MCM6)*<br>*ctf4-I901E / ctf4-I901E*	This study	N/A
Saccharomyces cerevisiae: YCPR66<br>*MAT***a** / *MATα*<br>*pep4Δ::kanMX / pep4Δ::kanMX*<br>*bar1Δ::hph-NT / bar1Δ::hphNT*<br>*ura3-1 / ura3-1::URA3(GAL-MCM2 GAL-CBP-TEV-MCM3)*<br>*his3-11 / his3-11::HIS3(GAL-CDC45iFLAG2 GAL-GAL4)*<br>*trp1-1::TRP1(GAL-PSF1 GAL-SLD5) / trp1-1::TRP1(GAL-MCM5 GAL-MCM4)*<br>*leu2-3::LEU2(GAL-PSF2 GAL-PSF3) / leu2-3::LEU2(GAL-mcm7-10R GAL-MCM6)*<br>*ctf4-I901E / ctf4-I901E*	This study	N/A
Saccharomyces cerevisiae: YCPR68<br>*MAT***a**<br>*mcm7-10R (HIS3MX)*	This study	N/A
Saccharomyces cerevisiae: YCPR79<br>*MAT***a**<br>*pep4Δ::ADE2*<br>*TAP-SLD5 (kanMX)*<br>*mcm7-10R (HIS3MX)*	This study	N/A
Saccharomyces cerevisiae: YCPR83<br>*MAT***a** / *MATα*<br>*MCM7 / mcm7-10R (HIS3MX)*<br>*PIF1 / pif1Δ::URA3CP*<br>*RRM3 / rrm3Δ::hphNT*	This study	N/A
Saccharomyces cerevisiae: YCPR86<br>*MAT***a**<br>*pep4Δ::ADE2*<br>*cdc48-AID (hphNT)*<br>*ura3-1::URA3&K.l.TRP1(ADH1-OsTIR1-9MYC)*<br>*TAP-SLD5 (kanMX)*<br>*mcm7-10R (HIS3MX)*	This study	N/A
Saccharomyces cerevisiae: YCPR103	This study	N/A

MATa<br>*mcm7-10R (HIS3MX)*<br>*dia2-ΔTPR (hphNT)*		
Saccharomyces cerevisiae: YCPR111<br>*MATa*<br>*mcm7-10R (HIS3MX)*<br>*dia2-ΔTPR (hphNT)*<br>*TAP-SLD5 (kanMX)*<br>*pep4Δ::ADE2*	This study	N/A
Saccharomyces cerevisiae: YCPR138<br>*MATa*<br>*pif1Δ::URA3*<br>*TAP-SLD5 (kanMX)*<br>*pep4Δ::ADE2*	This study	N/A
Saccharomyces cerevisiae: YCPR141<br>*MATa*<br>*pif1Δ::URA3*<br>*TAP-SLD5 (kanMX)*<br>*pep4Δ::ADE2*<br>*mcm7-10R (HIS3MX)*	This study	N/A
Saccharomyces cerevisiae: YCPR143<br>*MATa*<br>*mcm7-10R (HIS3MX)*<br>*rrm3Δ::hphNT*<br>*TAP-SLD5 (kanMX)*<br>*pep4Δ::ADE2*	This study	N/A
Saccharomyces cerevisiae: YCPR334<br>*MATa*<br>*pep4Δ::ADE2*<br>*TAP-SLD5 (kanMX)*<br>*rrm3Δ::hphNT*<br>*mcm7-10R (HIS3MX)*<br>*leu2-3::LEU2(GAL-RRM3)*	This study	N/A
Saccharomyces cerevisiae: yCPR357<br>*MATa*<br>*TAP-SLD5 (kanMX)*<br>*pep4Δ::ADE2*<br>*pif1-m2*<br>*rrm3Δ::hphNT*	This study	N/A
Saccharomyces cerevisiae: YCPR359<br>*MATa*<br>*TAP-SLD5 (kanMX)*<br>*pep4Δ::ADE2*<br>*pif1-m2*	This study	N/A
Saccharomyces cerevisiae: YCPR406<br>*MATa*<br>*pep4Δ::ADE2*<br>*TAP-SLD5 (kanMX)*	This study	N/A

chl1Δ::URA3CP<br>*mcm7-10R (HIS3MX)*		
Saccharomyces cerevisiae: YCPR410<br>*MAT***a**<br>*pep4Δ::ADE2*<br>*TAP-SLD5 (kanMX)*<br>*chl1Δ::URA3CP*	This study	N/A
Saccharomyces cerevisiae: YCPR412<br>*MAT***a**<br>*pep4Δ::ADE2*<br>*TAP-SLD5 (kanMX)*<br>*mcm7-10R (HIS3MX)*<br>*srs2Δ::kanMX*	This study	N/A
Saccharomyces cerevisiae: YCPR421<br>*MAT***a**<br>*pep4Δ::ADE2*<br>*TAP-SLD5 (kanMX)*<br>*srs2Δ::kanMX*	This study	N/A
Saccharomyces cerevisiae: YCPR428<br>*MAT***a**<br>*pep4Δ::ADE2*<br>*TAP-SLD5 (kanMX)*<br>*sgs1Δ::URA3CP*	This study	N/A
Saccharomyces cerevisiae: YCPR429<br>*MAT***a**<br>*pep4Δ::ADE2*<br>*TAP-SLD5 (kanMX)*<br>*sgs1Δ::URA3CP*<br>*mcm7-10R (HIS3MX)*	This study	N/A
Saccharomyces cerevisiae: YCPR445<br>*MAT***a**<br>*RAD52-GFP (TRP1)*<br>*mcm7-10R (HIS3MX)*	This study	N/A
Saccharomyces cerevisiae: YCPR447<br>*MAT***a**<br>*RAD52-GFP (TRP1)*<br>*mcm7-10R (HIS3MX)*<br>*dia2-ΔTPR (hphNT)*	This study	N/A
Saccharomyces cerevisiae: YHM28<br>*MAT***a**<br>*dia2Δ::HIS3*	H. Morohashi	N/A
Saccharomyces cerevisiae: YHM130<br>*MAT***a**<br>*dia2Δ::HIS3*<br>*TAP2-SLD5 (KanMX)*<br>*ADE2*<br>*pep4Δ::URA3*	H. Morohashi	N/A
Saccharomyces cerevisiae: YHM306	H. Morohashi	N/A

*MAT***a**<br>*dia2-ΔTPR (hphNT)*		
Saccharomyces cerevisiae: YPM224<br>*MAT***a** / *MATα*<br>*pep4Δ::kanMX / pep4Δ::kanMX*<br>*bar1Δ::hph-NT / bar1Δ::hphNT*<br>*ura3-1 / ura3-1::URA3(GAL-MCM2 GAL-CBP-TEV-MCM3)*<br>*his3-11 / his3-11::HIS3(GAL-CDC45iFLAG2 GAL-GAL4)*<br>*trp1-1::TRP1(GAL-PSF1 GAL-SLD5) / trp1-1::TRP1(GAL-MCM5 GAL-MCM4)*<br>*leu2-3::LEU2(GAL-PSF2 GAL-PSF3) / leu2-3::LEU2(GAL-mcm7-K29A GAL-MCM6)*<br>*ctf4-I901E / ctf4-I901E*	Deegan et al., 2020	N/A
Saccharomyces cerevisiae: YPM229<br>*MAT***a** / *MATα*<br>*pep4Δ::kanMX / pep4Δ::kanMX*<br>*bar1Δ::hph-NT / bar1Δ::hphNT*<br>*ura3-1 / ura3-1::URA3(GAL-MCM2 GAL-CBP-TEV-MCM3)*<br>*his3-11 / his3-11::HIS3(GAL-CDC45iFLAG2 GAL-GAL4)*<br>*trp1-1::TRP1(GAL-PSF1 GAL-SLD5) / trp1-1::TRP1(GAL-MCM5 GAL-MCM4)*<br>*leu2-3::LEU2(GAL-PSF2 GAL-PSF3) / leu2-3::LEU2(GAL-mcm7-3TEV(M167) GAL-MCM6)*<br>*ctf4-I901E / ctf4-I901E*	This study	N/A
Saccharomyces cerevisiae: YPM230<br>*MAT***a** / *MATα*<br>*pep4Δ::kanMX / pep4Δ::kanMX*<br>*bar1Δ::hph-NT / bar1Δ::hphNT*<br>*ura3-1 / ura3-1::URA3(GAL-MCM2 GAL-CBP-TEV-MCM3)*<br>*his3-11 / his3-11::HIS3(GAL-CDC45iFLAG2 GAL-GAL4)*<br>*trp1-1::TRP1(GAL-PSF1 GAL-SLD5) / trp1-1::TRP1(GAL-MCM5 GAL-MCM4)*<br>*leu2-3::LEU2(GAL-PSF2 GAL-PSF3) / leu2-3::LEU2(GAL-mcm7-3TEV(A219) GAL-MCM6)*<br>*ctf4-I901E / ctf4-I901E*	This study	N/A
Saccharomyces cerevisiae: YPNK337<br>*MAT***a**<br>*pep4Δ::ADE2*<br>*cdc48-AID (hphNT)*<br>*ura3-1::URA3&K.l.TRP1(ADH1-OsTIR1-9MYC)*<br>*TAP-SLD5 (kanMX)*	P. Nkosi	N/A
Saccharomyces cerevisiae: YSS47<br>*MAT***a**<br>*pep4Δ::ADE2*<br>*TAP-SLD5 (kanMX)*	S. Sengupta	N/A
Saccharomyces cerevisiae: YTM74<br>*MAT***a**<br>*RAD52-GFP (TRP1)*	T. Maculins	N/A

dia2-ΔTPR (hph-NT)		
Saccharomyces cerevisiae: YTM115<br>*MAT***a**<br>*RAD52-GFP (TRP1)*<br>*dia2Δ::HIS3*	T. Maculins	N/A
Saccharomyces cerevisiae: YTM265<br>*MAT***a**<br>*TAP2-SLD5 (KanMX)*<br>*pep4Δ::URA3*<br>*ADE2*<br>*dia2-ΔTPR (hphNT)*	T. Maculins	N/A
Saccharomyces cerevisiae: YPM224<br>*MAT***a** / *MATα*<br>*pep4Δ::kanMX / pep4Δ::kanMX*<br>*bar1Δ::hph-NT / bar1Δ::hphNT*<br>*ura3-1 / ura3-1::URA3(GAL-MCM2 GAL-CBP-TEV-MCM3)*<br>*his3-11 / his3-11::HIS3(GAL-CDC45iFLAG2 GAL-GAL4)*<br>*trp1-1::TRP1(GAL-PSF1 GAL-SLD5) / trp1-1::TRP1(GAL-MCM5 GAL-MCM4)*<br>*leu2-3::LEU2(GAL-PSF2 GAL-PSF3) / leu2-3::LEU2(GAL-mcm7-K29A GAL-MCM6)*<br>*ctf4-I901E / ctf4-I901E*	Deegan et al., 2020	N/A
Saccharomyces cerevisiae: YPM229<br>*MAT***a** / *MATα*<br>*pep4Δ::kanMX / pep4Δ::kanMX*<br>*bar1Δ::hph-NT / bar1Δ::hphNT*<br>*ura3-1 / ura3-1::URA3(GAL-MCM2 GAL-CBP-TEV-MCM3)*<br>*his3-11 / his3-11::HIS3(GAL-CDC45iFLAG2 GAL-GAL4)*<br>*trp1-1::TRP1(GAL-PSF1 GAL-SLD5) / trp1-1::TRP1(GAL-MCM5 GAL-MCM4)*<br>*leu2-3::LEU2(GAL-PSF2 GAL-PSF3) / leu2-3::LEU2(GAL-mcm7-3TEV(M167) GAL-MCM6)*<br>*ctf4-I901E / ctf4-I901E*	This study	N/A
Saccharomyces cerevisiae: YPM230<br>*MAT***a** / *MATα*<br>*pep4Δ::kanMX / pep4Δ::kanMX*<br>*bar1Δ::hph-NT / bar1Δ::hphNT*<br>*ura3-1 / ura3-1::URA3(GAL-MCM2 GAL-CBP-TEV-MCM3)*<br>*his3-11 / his3-11::HIS3(GAL-CDC45iFLAG2 GAL-GAL4)*<br>*trp1-1::TRP1(GAL-PSF1 GAL-SLD5) / trp1-1::TRP1(GAL-MCM5 GAL-MCM4)*<br>*leu2-3::LEU2(GAL-PSF2 GAL-PSF3) / leu2-3::LEU2(GAL-mcm7-3TEV(A219) GAL-MCM6)*<br>*ctf4-I901E / ctf4-I901E*	This study	N/A
Saccharomyces cerevisiae: YCPR33<br>*MAT***a** / *MATα*	This study	N/A

pep4Δ::kanMX / pep4Δ::kanMX<br>*bar1Δ::hph-NT / bar1Δ::hphNT*<br>*ura3-1 / ura3-1::URA3(GAL-MCM2 GAL-CBP-TEV-MCM3)*<br>*his3-11 / his3-11::HIS3(GAL-CDC45iFLAG2 GAL-GAL4)*<br>*trp1-1::TRP1(GAL-PSF1 GAL-SLD5) / trp1-1::TRP1(GAL-MCM5 GAL-MCM4)*<br>*leu2-3::LEU2(GAL-PSF2 GAL-PSF3) / leu2-3::LEU2(GAL-mcm7-3TEV(T394) GAL-MCM6)*<br>*ctf4-I901E / ctf4-I901E*		
Saccharomyces cerevisiae: YCPR38<br>*MAT***a** / *MATα*<br>*pep4Δ::kanMX / pep4Δ::kanMX*<br>*bar1Δ::hph-NT / bar1Δ::hphNT*<br>*ura3-1 / ura3-1::URA3(GAL-MCM2 GAL-CBP-TEV-MCM3)*<br>*his3-11 / his3-11::HIS3(GAL-CDC45iFLAG2 GAL-GAL4)*<br>*trp1-1::TRP1(GAL-PSF1 GAL-SLD5) / trp1-1::TRP1(GAL-MCM5 GAL-MCM4)*<br>*leu2-3::LEU2(GAL-PSF2 GAL-PSF3) / leu2-3::LEU2(GAL-mcm7-10R-3TEV(A219) GAL-MCM6)*<br>*ctf4-I901E / ctf4-I901E*	This study	N/A
Saccharomyces cerevisiae: YCPR66<br>*MAT***a** / *MATα*<br>*pep4Δ::kanMX / pep4Δ::kanMX*<br>*bar1Δ::hph-NT / bar1Δ::hphNT*<br>*ura3-1 / ura3-1::URA3(GAL-MCM2 GAL-CBP-TEV-MCM3)*<br>*his3-11 / his3-11::HIS3(GAL-CDC45iFLAG2 GAL-GAL4)*<br>*trp1-1::TRP1(GAL-PSF1 GAL-SLD5) / trp1-1::TRP1(GAL-MCM5 GAL-MCM4)*<br>*leu2-3::LEU2(GAL-PSF2 GAL-PSF3) / leu2-3::LEU2(GAL-mcm7-10R GAL-MCM6)*<br>*ctf4-I901E / ctf4-I901E*	This study	N/A
Saccharomyces cerevisiae: YPNK337<br>*MAT***a**<br>*pep4Δ::ADE2*<br>*cdc48-AID (hphNT)*<br>*ura3-1::URA3&K.l.TRP1(ADH1-OsTIR1-9MYC)*<br>*TAP-SLD5 (kanMX)*	P. Nkosi	N/A
Saccharomyces cerevisiae: YCPR86<br>*MAT***a**<br>*pep4Δ::ADE2*<br>*cdc48-AID (hphNT)*<br>*ura3-1::URA3&K.l.TRP1(ADH1-OsTIR1-9MYC)*<br>*TAP-SLD5 (kanMX)*<br>*mcm7-10R (HIS3MX)*	This study	N/A
Saccharomyces cerevisiae: YSS47<br>*MAT***a**<br>*pep4Δ::ADE2*<br>*TAP-SLD5 (kanMX)*	S. Sengupta	N/A

Saccharomyces cerevisiae: YCPR79<br>*MAT***a**<br>*pep4Δ::ADE2*<br>*TAP-SLD5 (kanMX)*<br>*mcm7-10R (HIS3MX)*	This study	N/A
Saccharomyces cerevisiae: YHM130<br>*MAT***a**<br>*dia2Δ::HIS3*<br>*TAP2-SLD5 (KanMX)*<br>*ADE2*<br>*pep4Δ::URA3*	H. Morohashi	N/A
Saccharomyces cerevisiae: YTM265<br>*MAT***a**<br>*TAP2-SLD5 (KanMX)*<br>*pep4Δ::URA3*<br>*ADE2*<br>*dia2-ΔTPR (hphNT)*	T. Maculins	N/A
Saccharomyces cerevisiae: YCPR111<br>*MAT***a**<br>*mcm7-10R (HIS3MX)*<br>*dia2-ΔTPR (hphNT)*<br>*TAP-SLD5 (kanMX)*<br>*pep4Δ::ADE2*	This study	N/A
Saccharomyces cerevisiae: YHM28<br>*MAT***a**<br>*dia2Δ::HIS3*	H. Morohashi	N/A
Saccharomyces cerevisiae: YCPR103<br>*MAT***a**<br>*mcm7-10R (HIS3MX)*<br>*dia2-ΔTPR (hphNT)*	This study	N/A
Saccharomyces cerevisiae: YCPR68<br>*MAT***a**<br>*mcm7-10R (HIS3MX)*	This study	N/A
Saccharomyces cerevisiae: YHM306<br>*MAT***a**<br>*dia2-ΔTPR (hphNT)*	H. Morohashi	N/A
Saccharomyces cerevisiae: YBH295<br>*MAT***a**<br>*RAD52-GFP (TRP1)*	B. Hodgson	N/A
Saccharomyces cerevisiae: YTM115<br>*MAT***a**<br>*RAD52-GFP (TRP1)*<br>*dia2Δ::HIS3*	T. Maculins	N/A
Saccharomyces cerevisiae: YCPR447<br>*MAT***a**<br>*RAD52-GFP (TRP1)*<br>*mcm7-10R (HIS3MX)*<br>*dia2-ΔTPR (hphNT)*	This study	N/A

Saccharomyces cerevisiae: YCPR445<br>*MAT***a**<br>*RAD52-GFP (TRP1)*<br>*mcm7-10R (HIS3MX)*	This study	N/A
Saccharomyces cerevisiae: YTM74<br>*MAT***a**<br>*RAD52-GFP (TRP1)*<br>*dia2-ΔTPR (hph-NT)*	T. Maculins	N/A
Saccharomyces cerevisiae: YCPR83<br>*MAT***a** */ MATα*<br>*MCM7 / mcm7-10R (HIS3MX)*<br>*PIF1 / pif1Δ::URA3CP*<br>*RRM3 / rrm3Δ::hphNT*	This study	N/A
Saccharomyces cerevisiae: YCPR143<br>*MAT***a**<br>*mcm7-10R (HIS3MX)*<br>*rrm3Δ::hphNT*<br>*TAP-SLD5 (kanMX)*<br>*pep4Δ::ADE2*	This study	N/A
Saccharomyces cerevisiae: YCPR138<br>*MAT***a**<br>*pif1Δ::URA3*<br>*TAP-SLD5 (kanMX)*<br>*pep4Δ::ADE2*	This study	N/A
Saccharomyces cerevisiae: YCPR141<br>*MAT***a**<br>*pif1Δ::URA3*<br>*TAP-SLD5 (kanMX)*<br>*pep4Δ::ADE2*<br>*mcm7-10R (HIS3MX)*	This study	N/A
Saccharomyces cerevisiae: YCPR421<br>*MAT***a**<br>*pep4Δ::ADE2*<br>*TAP-SLD5 (kanMX)*<br>*srs2Δ::kanMX*	This study	N/A
Saccharomyces cerevisiae: YCPR412<br>*MAT***a**<br>*pep4Δ::ADE2*<br>*TAP-SLD5 (kanMX)*<br>*mcm7-10R (HIS3MX)*<br>*srs2Δ::kanMX*	This study	N/A
Saccharomyces cerevisiae: YCPR428<br>*MAT***a**<br>*pep4Δ::ADE2*<br>*TAP-SLD5 (kanMX)*<br>*sgs1Δ::URA3CP*	This study	N/A
Saccharomyces cerevisiae: YCPR429<br>*MAT***a**	This study	N/A

pep4Δ::ADE2<br>*TAP-SLD5 (kanMX)*<br>*sgs1Δ::URA3CP*<br>*mcm7-10R (HIS3MX)*		
Saccharomyces cerevisiae: YCPR410<br>*MAT***a**<br>*pep4Δ::ADE2*<br>*TAP-SLD5 (kanMX)*<br>*chl1Δ::URA3CP*	This study	N/A
Saccharomyces cerevisiae: YCPR406<br>*MAT***a**<br>*pep4Δ::ADE2*<br>*TAP-SLD5 (kanMX)*<br>*chl1Δ::URA3CP*<br>*mcm7-10R (HIS3MX)*	This study	N/A
Saccharomyces cerevisiae: YCPR359<br>*MAT***a**<br>*TAP-SLD5 (kanMX)*<br>*pep4Δ::ADE2*<br>*pif1-m2*	This study	N/A
Saccharomyces cerevisiae: yCPR357<br>*MAT***a**<br>*TAP-SLD5 (kanMX)*<br>*pep4Δ::ADE2*<br>*pif1-m2*<br>*rrm3Δ::hphNT*	This study	N/A
Saccharomyces cerevisiae: YCPR334<br>*MAT***a**<br>*pep4Δ::ADE2*<br>*TAP-SLD5 (kanMX)*<br>*rrm3Δ::hphNT*<br>*mcm7-10R (HIS3MX)*<br>*leu2-3::LEU2(GAL-RRM3)*	This study	N/A
**Recombinant DNA (Plasmids)**		
pCPR11 [Expression vector to express Mcm6 and Mcm7-10R-3TEV-A219 to produce recombinant CMG]	MRC PPU Reagents and Services (This study)	DU78160
pCPR12 [Expression vector to express Mcm6 and Mcm7-3TEV-T394 to produce recombinant CMG]	MRC PPU Reagents and Services (This study)	DU78172
pCPR18 [Expression vector to express Mcm6 and Mcm7-10R to produce recombinant CMG]	MRC PPU Reagents and Services (This study)	DU78161
pCPR33 [Vector based on pCPR18 to generate a DNA template for replacing endogenous Mcm7 with Mcm7-10R]	MRC PPU Reagents and	DU78162

	Services (This study)	
pCPR34 [Expression vector to express a second copy of the RRM3 gene controlled by the galactose promoter]	MRC PPU Reagents and Services (This study)	DU78163
pET21b Npl4 [to express yeast Npl4 in *E. coli*]	Stein et al., 2014	N/A
K27 SUMO CDC48 [to express 14His-SMT3-tagged version of yeast Cdc48 in *E. coli*]	Stein et al., 2014	N/A
K27 SUMO Ufd1 [to express yeast Ufd1 in *E. coli*]	Stein et al., 2014	N/A
pPM21 [to express Mcm6 and Mcm7-3TEV(M167) in yeast to produce recombinant CMG]	MRC PPU Reagents and Services (This study)	DU78174
pPM22 [Expression vector to express Mcm6 and Mcm7-3TEV-A219 to produce recombinant CMG]	MRC PPU Reagents and Services (This study)	DU78173
pPM51 [to express Mcm6 and Mcm7-K29A in yeast to produce recombinant CMG]	Deegan et al., 2020	N/A
pTDK3 [to express Skp1 and Dia2 subunits of SCF^{Dia2} in yeast]	Deegan et al., 2020	N/A
pTDK6 [to express Hrt1 and Cdc53 subunits of SCF^{Dia2} in yeast]	Deegan et al., 2020	N/A
pKL506 [Template for PCR amplification of the URA3CP cassette, for one-step gene deletion]	K. Labib	N/A
pYM18 [Template for PCR amplification of the kanMX cassette, for one-step gene deletion]	Janke et al (2004)	N/A
**Oligonucleotides**		
Check integration of pRS series vectors; 1802: GGCATCAGAGCAGATTGTAC	This study	N/A
Check integration of pRS series vectors; 1803: CTCCTTACGCATCTGTGCGG	This study	N/A
Check integration of pRS series vectors; 1806: ACAACGACCAAGCTCACATC	This study	N/A
Check integration of pRS series vectors; 1807: ACATTATGGGTGGTATGTTGG	This study	N/A
Check integration of pRS series vectors; 1810: TGGAGTTCAATGCGTCCATC	This study	N/A
Check integration of pRS series vectors; 1811: CTACTGCGCCAATTGATGAC	This study	N/A
To generate a fragment of Mcm7 containing 3TEV-M167 or 3TEV-A219; 8360: GAAAATTCAATATAAGGCGCGCCAT- GAGTGCGGCACTTCC	This study	N/A

To generate a fragment of Mcm7 containing 3TEV-M167 or 3TEV-A219; 8361: CGAATTCCTGCAGCCCGGGTCAAG- CGTCTTGTAGATCG	This study	N/A
To generate a fragment of Mcm7 containing 3TEV-T394; 8963: ATGGATGAGGAGGAAGACAATCTGTC	This study	N/A
To generate a fragment of Mcm7 containing 3TEV-T394; 8964: GACAGATTGTCTTCCTCCTCATCCAT	This study	N/A
To generate a fragment of Mcm7 containing 3TEV-T394; 8965: GAGGGTGGCTCGAACACCAAAGGC	This study	N/A
To generate a fragment of Mcm7 containing 3TEV-T394; 8965: GCCTTTGGTGTTCGAGCCACCCTC	This study	N/A
To generate a plasmid to express Mcm7-K29A; 8364: GAAAATTCAATATAAGGCGCGCCATGAGTGCGGCACTTCC	Deegan et al., 2020; This study	N/A
To generate a plasmid to express Mcm7-K29A; 8365: CGAATTCCTGCAGCCCGGGTCAAGCGTCTTGTAGATCG	Deegan et al., 2020; This study	N/A
To remove the 3TEV sites in Mcm7-10R- 3TEV-A219; 9004: TTTGAAAATTCAATATAAGGCGCGCCATGAGTGCGGCACTTCCATC	This study	N/A
To remove the 3TEV sites in Mcm7-10R- 3TEV-A219; 9005: GAACTAATTGCTCTTCTCCTGTAACGAC	This study	N/A
To remove the 3TEV sites in Mcm7-10R- 3TEV-A219; 9006: AGGAGAAGAGCAATTAGTTCTAAACCATTATCTGTTAG	This study	N/A
To amplify Mcm7- 10R-HIS3MX for allelic replacement to the endogenous MCM7 locus; 9182: AGTCGATTTGCTTCTTTTTTCACGAAAGTGAGATACCAGCAAAGGACCAGAGCGAGATGTACTACAGTAGCAGGCTACATACACGTCTTTACCAATAAATTACATTTAGAAAGGCAATATAATCACTACTTAGATTAGCTTCAATCTTGCAGGAAGAACTCAATACAGACAGACCAGATTATGAGTGCGGCACTTCCATC	This study	N/A
To amplify Mcm7- 10R-HIS3MX for allelic replacement to the endogenous MCM7 locus; 9183: AATATTAGTAAAATTATAATAATACAGAATACATATCTTGAAAACTGGGCCTTGGAAGTAAGTTACAGAAGGTAAATTTGAATGTGCAGCTATATGGAAATGAAAAAAAAGGGGGAAAAAGAATAAGGAATGAAGGCCCTGTTGCTTTTTTTTTTAGAACTTTCAAGCGTCTTGTAGATCATCGATGAATTCGAGCTCGT	This study	N/A
To amplify the RRM3 gene from genomic DNA to make GAL-RRM3; 7511: TCATGGGATCCATGTTCAGGTCGCATGCCTCCGGTAACAAG	This study	N/A
To amplify RRM3 gene from genomic DNA to make GAL-RRM3; 7512: TAGTACCCCGGGTCATTTCAAAGTTTCTAAACGTTTATAG	This study	N/A
To knockout the SRS2 gene with the kanMX cassette from pYM18; 9844:	This study	N/A

GAGTATCATTCCAATTTGATCTTTCTTCTACCGGTACTTAGGG ATAGCAAGACATGGAGGCCCAGAATAC		
To knockout the SRS2 gene with the kanMX cassette from pYM18; 9845: AAATTATAAACCGCCTCCAATAGTTGACGTAGTCAGGCATGA AAGTGCTACAGTATAGCGACCAGCATTC	This study	N/A
To check SRS2 deletion with the kanMX cassette; 9846: CTTGTATACACAACCACTGC	This study	N/A
To check SRS2 dele- tion with the kanMX cassette; 9847: TAAGCCTTCTACGCAAGACG	This study	N/A
To check SRS2 dele- tion with the kanMX cassette; 9848: TGACGAGCGTAATGGCTGGC	This study	N/A
To knockout the SRS2 gene with the kanMX cassette from pYM18; 9844: GAGTATCATTCCAATTTGATCTTTCTTCTACCGGTACTTAGGG ATAGCAAGACATGGAGGCCCAGAATAC	This study	N/A
To knockout the SRS2 gene with the kanMX cassette from pYM18; 9845: AAATTATAAACCGCCTCCAATAGTTGACGTAGTCAGGCATGA AAGTGCTACAGTATAGCGACCAGCATTC	This study	N/A
To check SRS2 deletion with the kanMX cassette; 9846: CTTGTATACACAACCACTGC	This study	N/A
To check SRS2 deletion with the kanMX cassette; 9847: TAAGCCTTCTACGCAAGACG	This study	N/A
To check SRS2 deletion with the kanMX cassette; 9848: TGACGAGCGTAATGGCTGGC	This study	N/A
To check SRS2 deletion with the kanMX cassette; 9849: GCCAGCCATTACGCTCGTCA	This study	N/A
To knockout the CHL1 gene with the URA3CP cassette from pKL506; 9827: ACCCAAAAGAGTAGAAAACCAGGCTAAAAACAGTCACACTAG TCCAAAAAATTAAGGCGCGCCAGATCTG	This study	N/A
To knockout the CHL1 gene with the URA3CP cassette from pKL506; 9828: TTACTATAATATATAGTAGTAATCACAGTATACACGTAAACGT ATTCCTTATCGATGAATTCGAGCTCGT	This study	N/A
To check CHL1 deletion with the URA3CP cassette; 9829: GCTATTAATCTCTTGCCTGC	This study	N/A
To check CHL1 deletion with the URA3CP cassette; 9830: GCTTGCGTATTATCTATAGC	This study	N/A
For sequencing TEV insertions and point mutants in Mcm7; 8919: GGCACTTCCATCAATTCAGC	This study	N/A
For sequencing TEV insertions and point mutants in Mcm7; 8920: CCTAGGCCAATTGATTACCG	This study	N/A
For sequencing TEV insertions and point mutants in Mcm7; 8921: GTTGCTGCTATTACTTGTCG	This study	N/A
For sequencing TEV insertions and point mutants in Mcm7; 8922:	This study	N/A

ACCAAGTAGAGATGACGACG		
For sequencing TEV insertions and point mutants in Mcm7; 8923: TTCTCAGAAGCACCTGCAGC	This study	N/A
For sequencing TEV insertions and point mutants in Mcm7; 8924: CGATATCAGAATCTTGGGCG	This study	N/A
For sequencing the *RRM3* gene; 6861: CGTCGGGATCATCATCTCC	This study	N/A
For sequencing the *RRM3* gene; 6862: CGCTTGGTATCTCTCGGG	This study	N/A
For sequencing the *RRM3* gene; 6852: CCGGTAACAAGAAGCAATGG	This study	N/A
For sequencing the *RRM3* gene; 6853: GCTGAATTCAAGAAGTTGGTCG	This study	N/A
For sequencing the *RRM3* gene; 6854: CCACAGATCCGCTTCTTCC	This study	N/A
For sequencing the *RRM3* gene; 6855: GGTTGACGGTAACTTGTTGG	This study	N/A
For sequencing the *RRM3* gene; 6856: CGATTACGCTGACGGTATCG	This study	N/A
For sequencing the *RRM3* gene; 6857: GGAAATCTACAAGATCGTCG	This study	N/A
**Software and Algorithms**		
AlphaFold2-multimer (ColabFold v1.5.3)	Google Deepmind	https://colab.research.google.com/github/sokrypton/ColabFold/blob/main/AlphaFold2.ipynb
Prism 9	GraphPad	https://www.grap hpad.com/scienti ficsoftware/prism/
FlowJo	BD Biosciences	https://www.flowj o.com/
ImageJ	NIH	N/A
