# Supplementary material for: CMG helicase disassembly is essential and driven by two pathways in budding yeast
Source: EMBO J. 2024 Jul 22;43(18):2. doi: 10.1038/s44318-024-00161-x (PMC11405719; doi:10.1038/s44318-024-00161-x)
Supplement: Supplementary file 7 — Source data Fig. 1 [file 44318_2024_161_MOESM7_ESM.zip › Source Data_Figure 1/1E/Figure 1E_Blots_Mcm7.pdf]

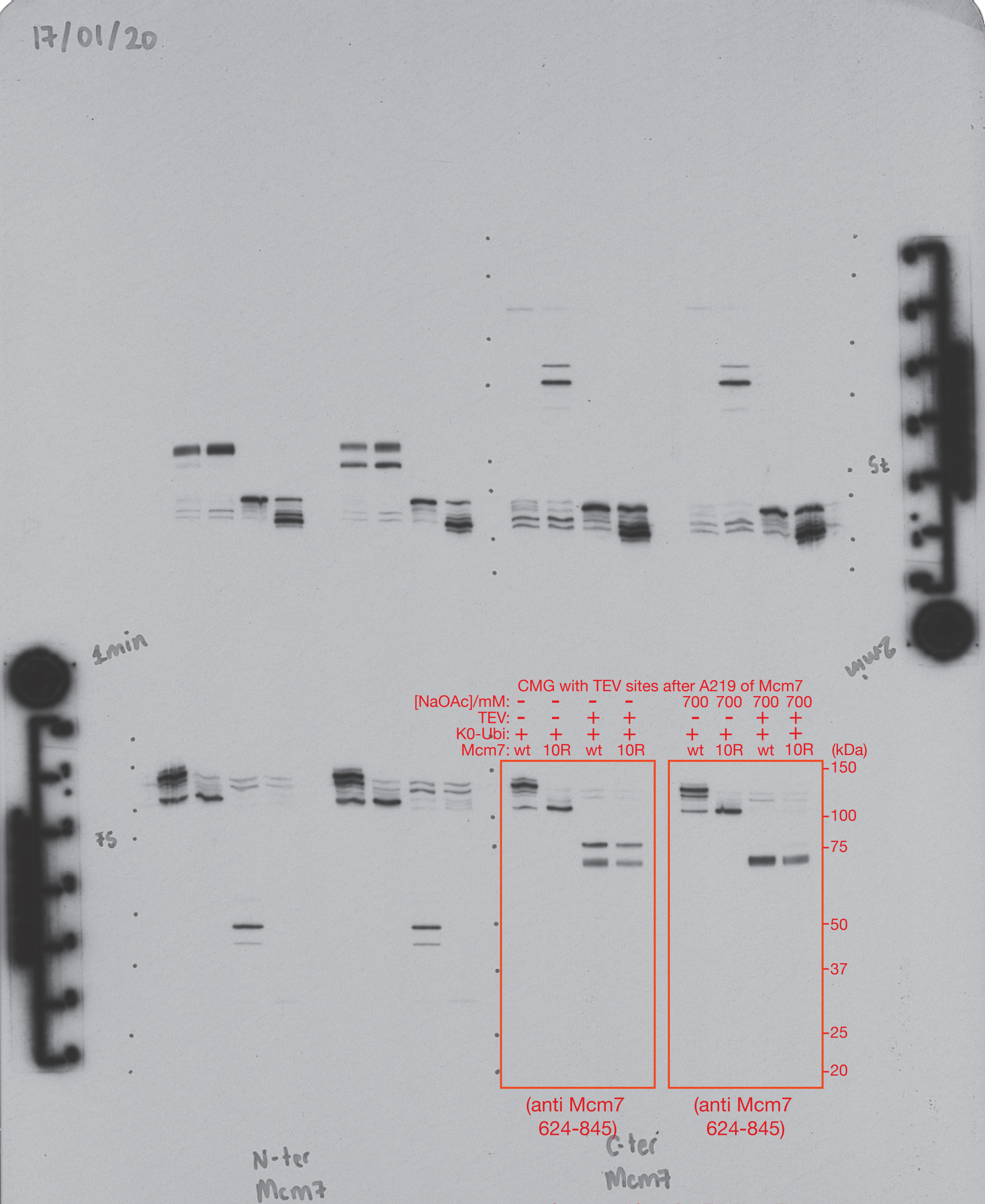

Immunoblots for Figure 1E
