# Supplementary figures and images for: CMG helicase disassembly is essential and driven by two pathways in budding yeast
Source: EMBO J. 2024 Jul 22;43(18):2. doi: 10.1038/s44318-024-00161-x (PMC11405719; doi:10.1038/s44318-024-00161-x)

16/08/19

15 min

CMG-3TEV-T394

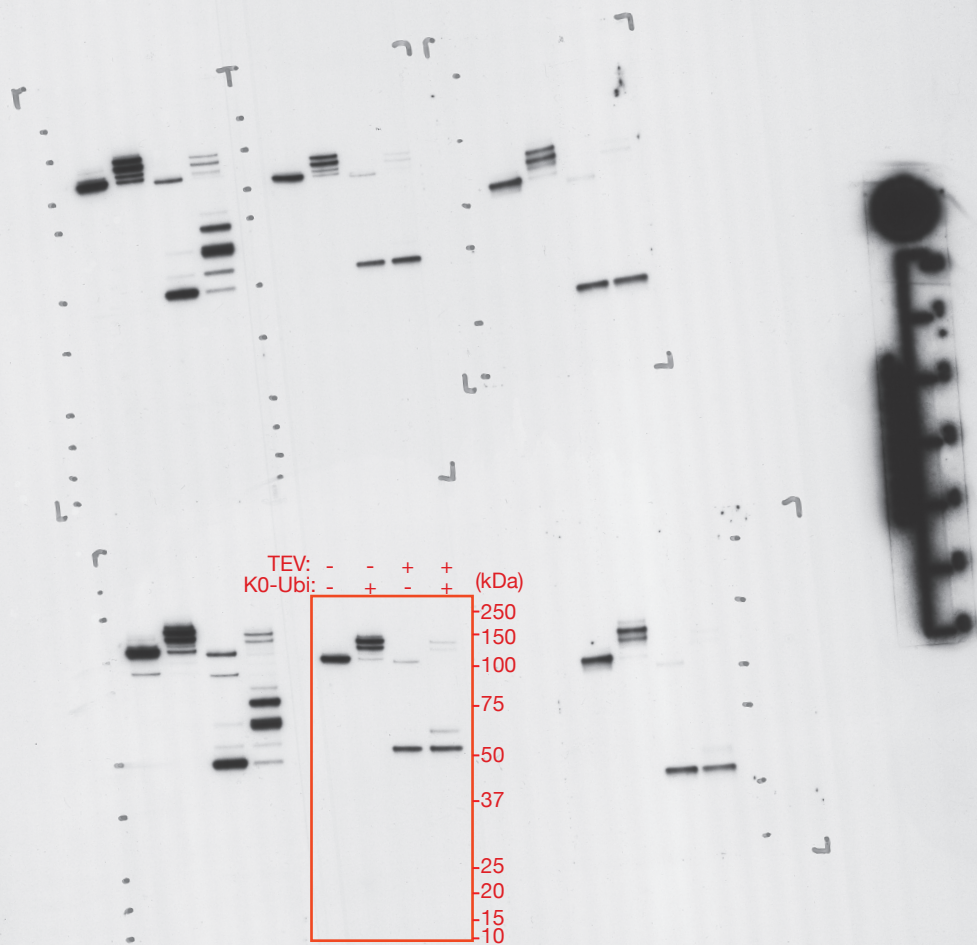

Immunoblot for Figure 1A  
(anti Mcm7 624-845)

Supplement: Supplementary file 7 — Source data Fig. 1 [file 44318_2024_161_MOESM7_ESM.zip › Source Data_Figure 1/1A/Figure 1A - Blot_anti Mcm7 624-845.pdf]

16/08/19

2min

TEV: - - + +  
K0-Ubi: - + - + (kDa)

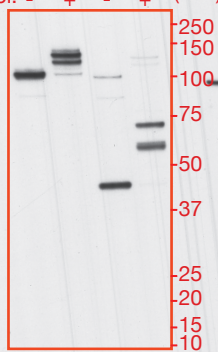

Immunoblot for Figure 1A  
(anti Mcm7 1-222)

Supplement: Supplementary file 7 — Source data Fig. 1 [file 44318_2024_161_MOESM7_ESM.zip › Source Data_Figure 1/1A/Figure 1A - Blot_anti Mcm7 1-222.pdf]

Immunoblots for Figure 1B

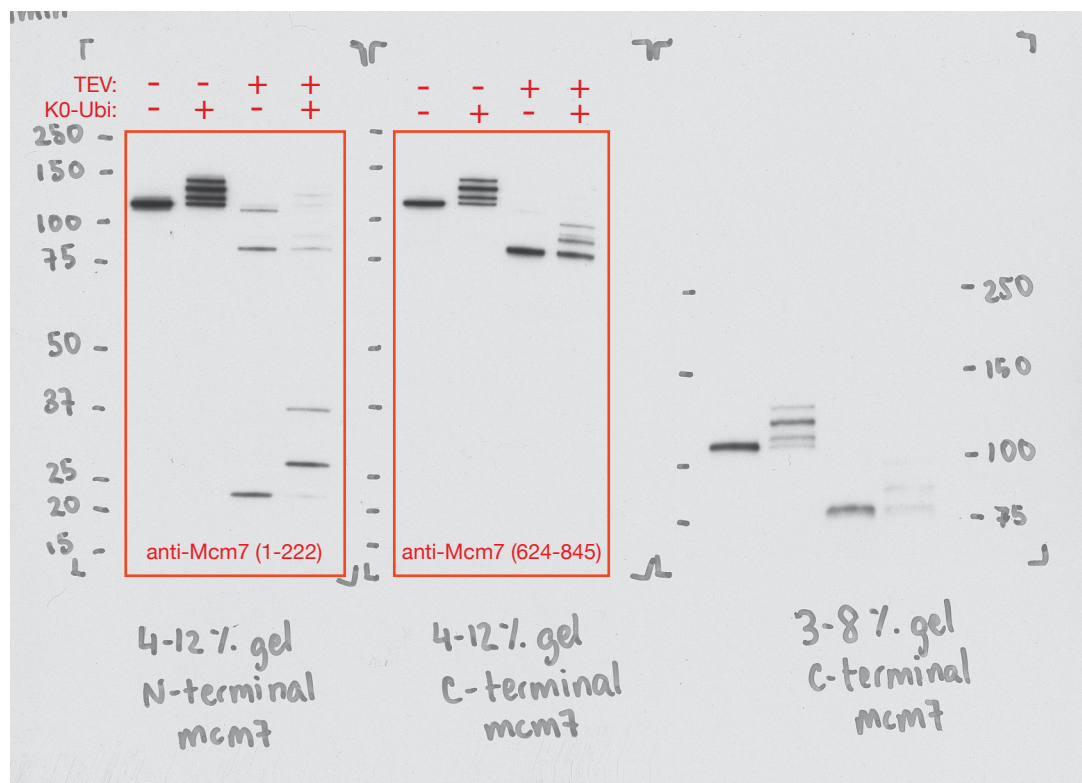

Supplement: Supplementary file 7 — Source data Fig. 1 [file 44318_2024_161_MOESM7_ESM.zip › Source Data_Figure 1/1B/Figure 1B_Blots_Mcm7.pdf]

07/05/2019

1min

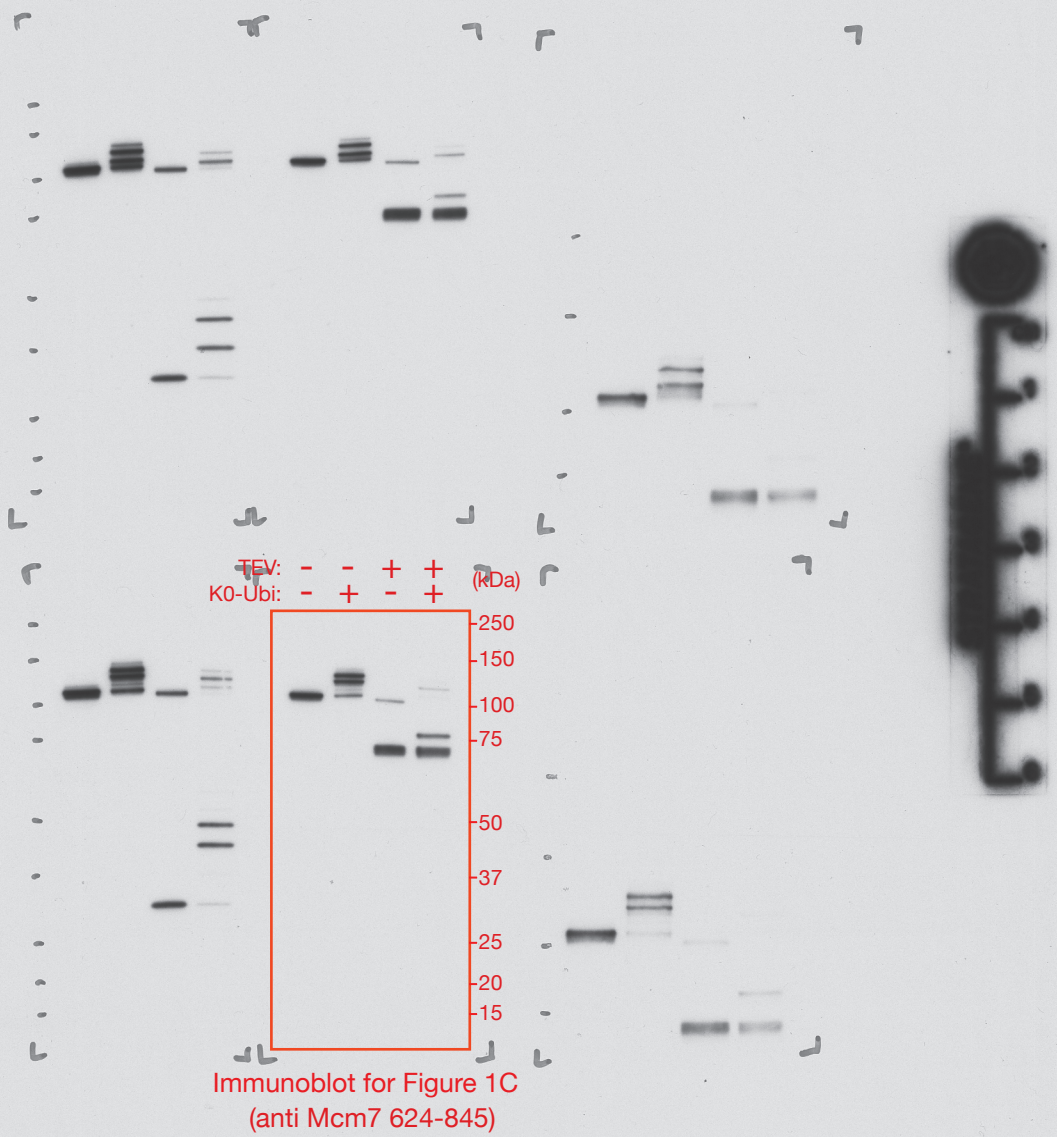

Supplement: Supplementary file 7 — Source data Fig. 1 [file 44318_2024_161_MOESM7_ESM.zip › Source Data_Figure 1/1C/Figure 1C - Blot_Mcm7 anti 624-845.pdf]

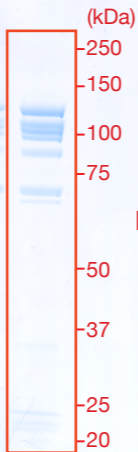

Image crop for Figure 2A

Supplement: Supplementary file 8 — Source data Fig. 2 [file 44318_2024_161_MOESM8_ESM.zip › Source Data_Figure 2/2A/Figure 2A_Coomassie gel.pdf]

10/03/20

Long (2h30')

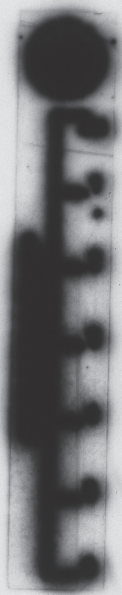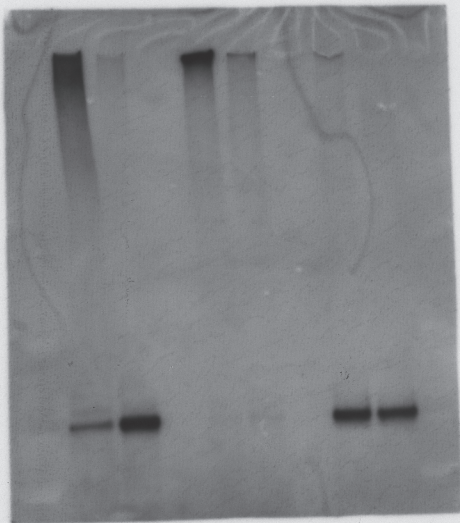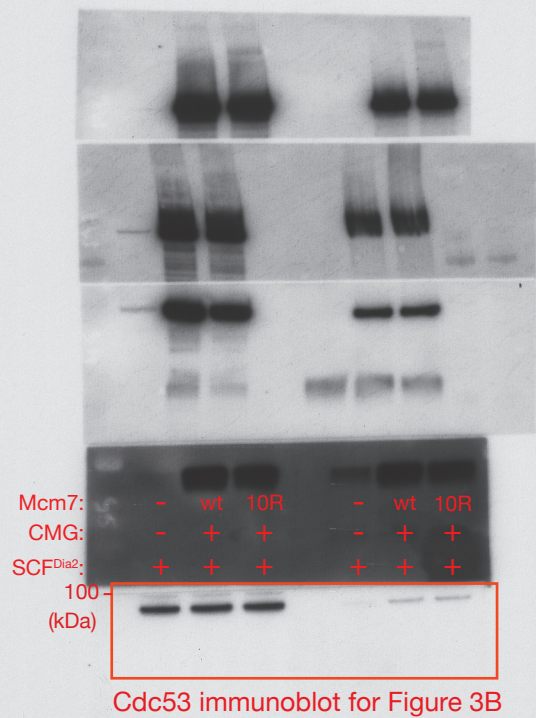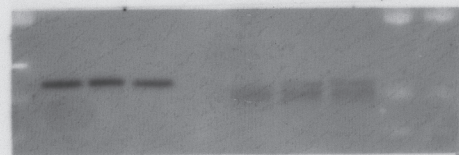

Supplement: Supplementary file 9 — Source data Fig. 3 [file 44318_2024_161_MOESM9_ESM.zip › Source Data_Figure 3/3B/Figure 3B_Blot_Cdc53.pdf]

long

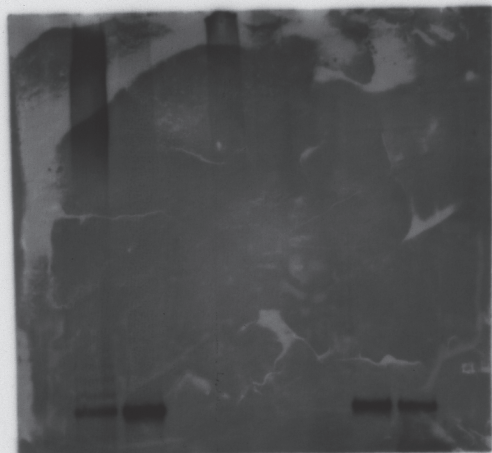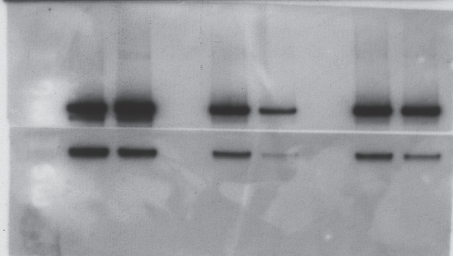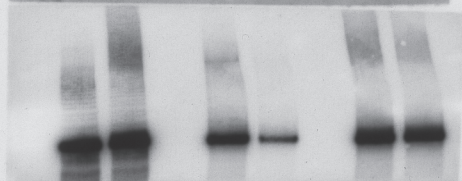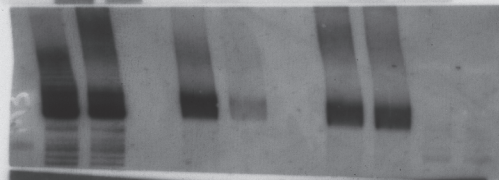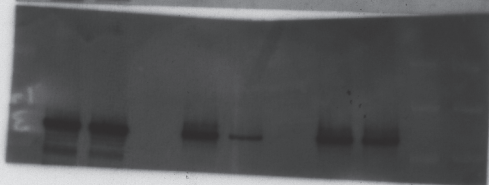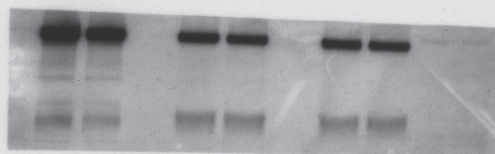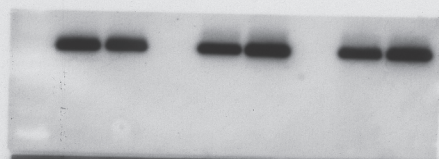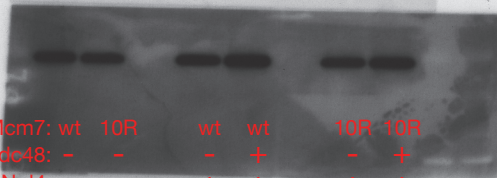

Mcm7: wt 10R wt wt 10R 10R  
 Cdc48: - - - + - +  
 Ufd1-Npl4: - - + + + +

25-  
 (kDa)

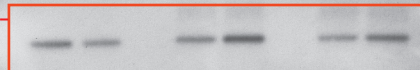

Psf3 immunoblot for Figure 3D

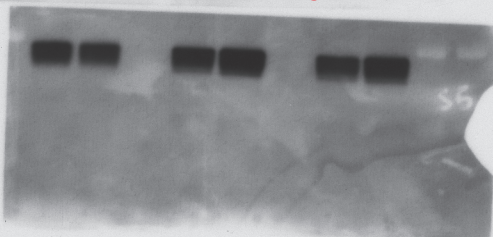

Supplement: Supplementary file 9 — Source data Fig. 3 [file 44318_2024_161_MOESM9_ESM.zip › Source Data_Figure 3/3D/Figure 3D_Blot_Psf3.pdf]

10/03/20

2min

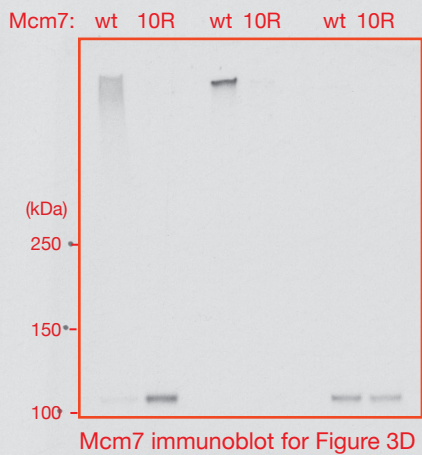

M7

M6

M3

C45

S5

C53

Skp1

Supplement: Supplementary file 9 — Source data Fig. 3 [file 44318_2024_161_MOESM9_ESM.zip › Source Data_Figure 3/3D/Figure 3D_Blot_Mcm7.pdf]

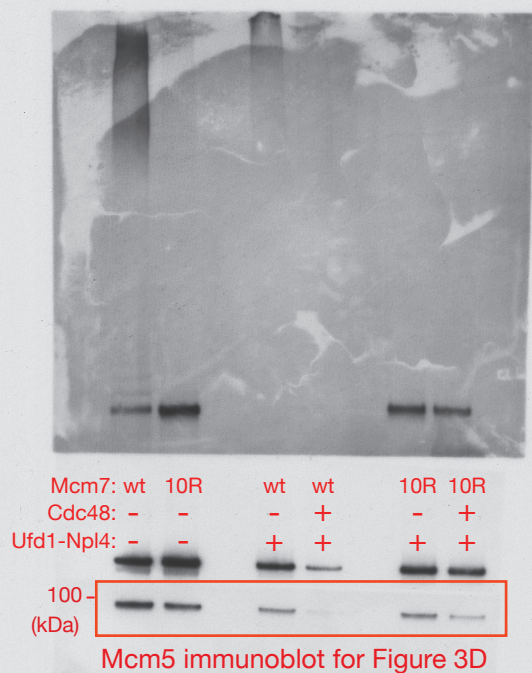

Supplement: Supplementary file 9 — Source data Fig. 3 [file 44318_2024_161_MOESM9_ESM.zip › Source Data_Figure 3/3D/Figure 3D_Blot_Mcm5.pdf]

23/07/21

20 sec

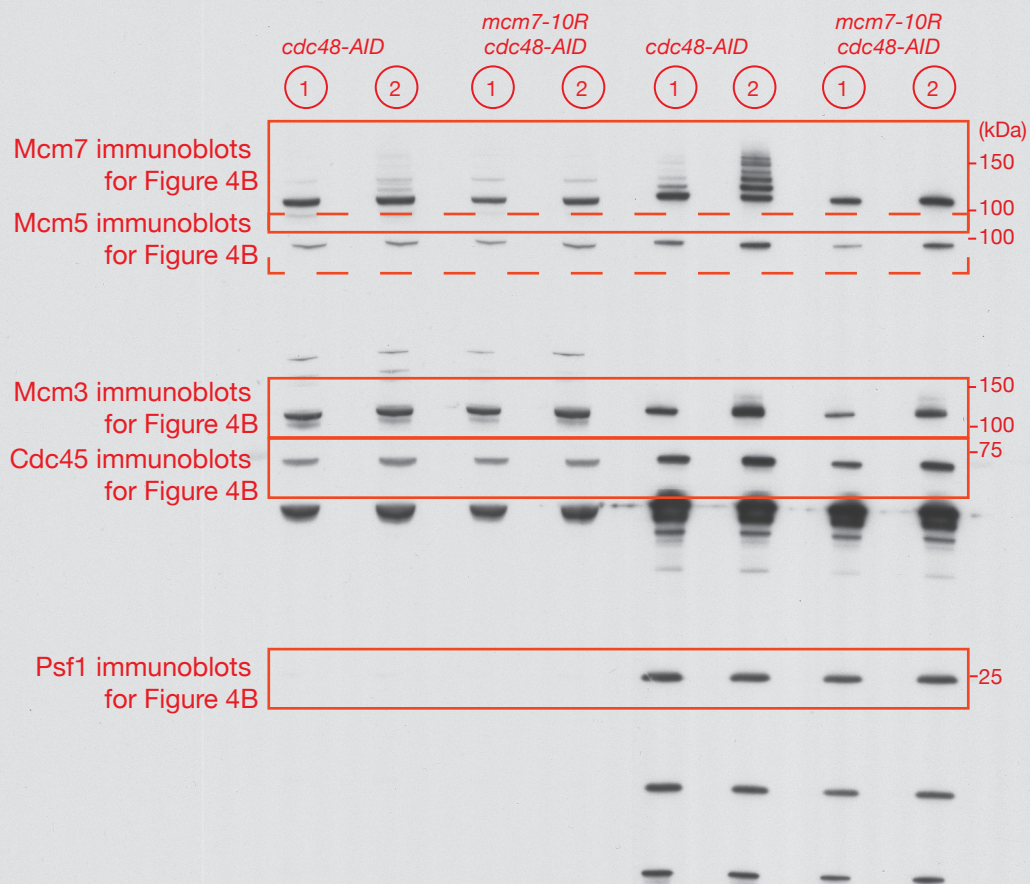

Supplement: Supplementary file 10 — Source data Fig. 4 [file 44318_2024_161_MOESM10_ESM.zip › Source Data_Figure 4/4B/Figure 4B_Blots_Mcm7-Mcm5-Mcm3-Cdc45-Psf1.pdf]

23/07/21

5 sec

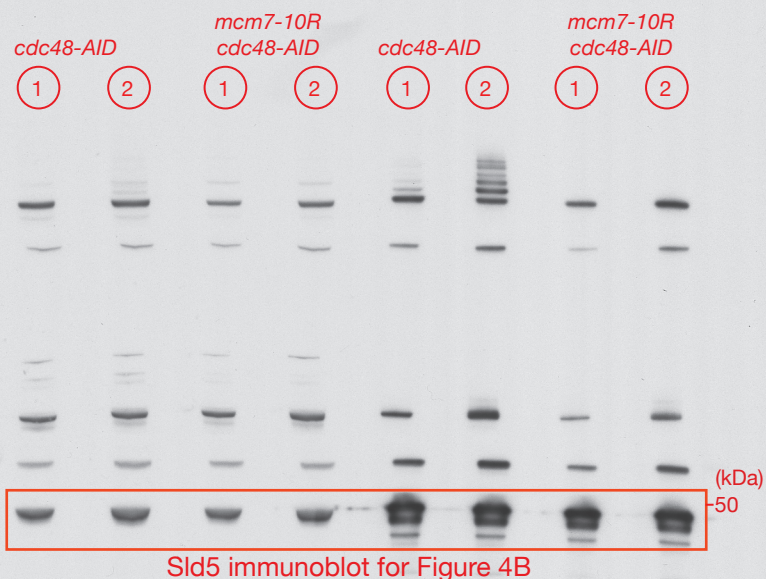

Sld5 immunoblot for Figure 4B

Supplement: Supplementary file 10 — Source data Fig. 4 [file 44318_2024_161_MOESM10_ESM.zip › Source Data_Figure 4/4B/Figure 4B_Blot_Sld5.pdf]

25/11/20  
1min

MCM7: wt      10R      wt      10R

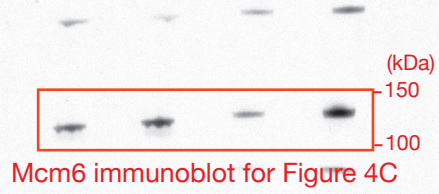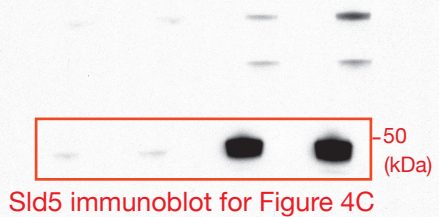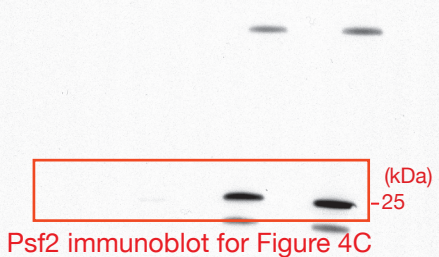

Supplement: Supplementary file 10 — Source data Fig. 4 [file 44318_2024_161_MOESM10_ESM.zip › Source Data_Figure 4/4C/Figure 4C_Blots_Mcm6-Psf2-Sld5.pdf]

25/11/20

5min

MCM7: wt 10R wt 10R

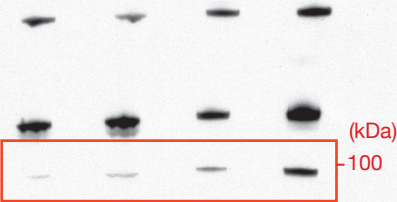

Mcm5 immunoblot for Figure 4C

Supplement: Supplementary file 10 — Source data Fig. 4 [file 44318_2024_161_MOESM10_ESM.zip › Source Data_Figure 4/4C/Figure 4C_Blot_Mcm5.pdf]

05/08/22

8min

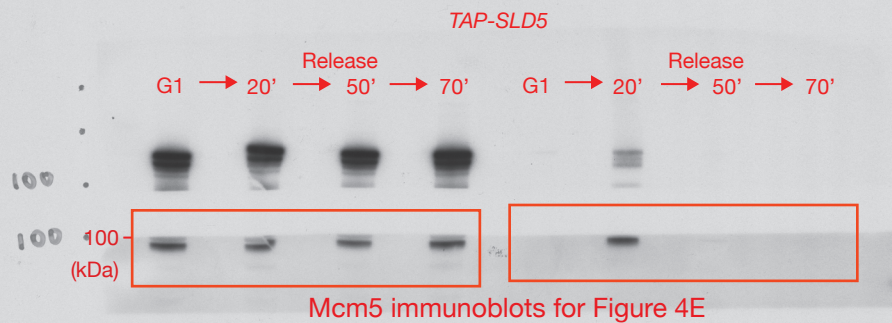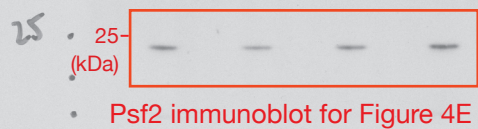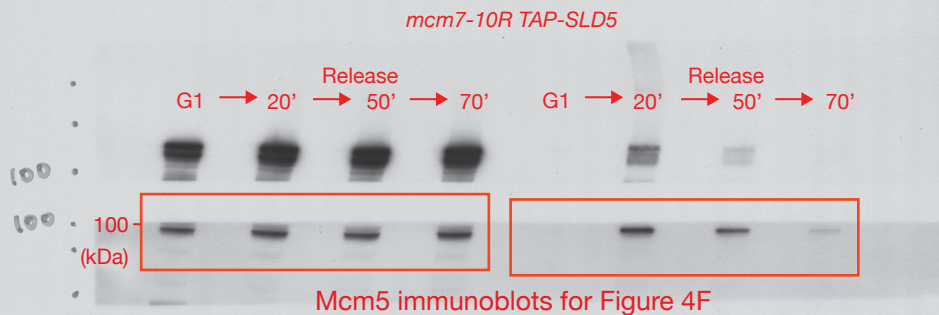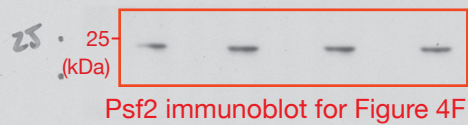

Supplement: Supplementary file 10 — Source data Fig. 4 [file 44318_2024_161_MOESM10_ESM.zip › Source Data_Figure 4/4E-F/Figure 4E-F_Blots_Mcm5-Psf2.pdf]

>1h  
02/08/22

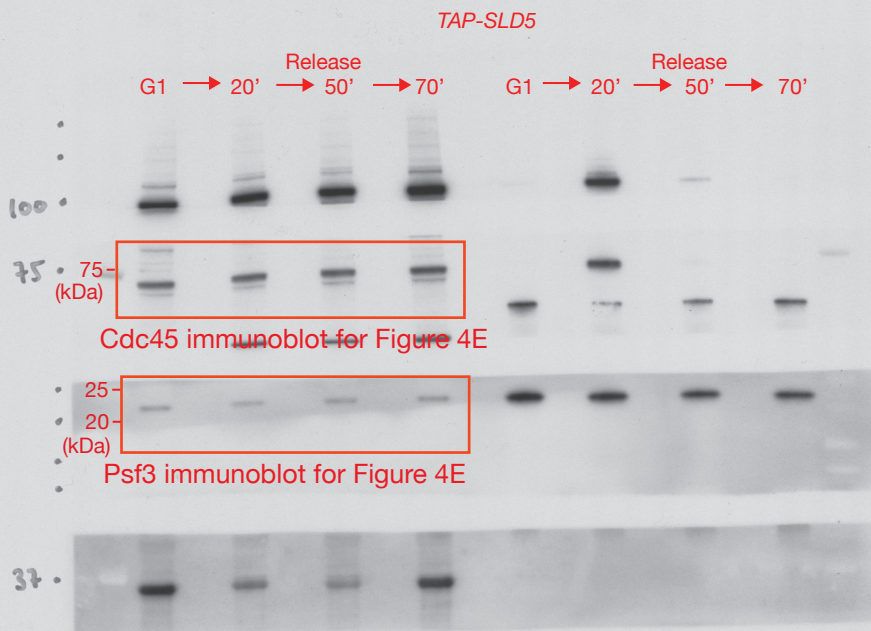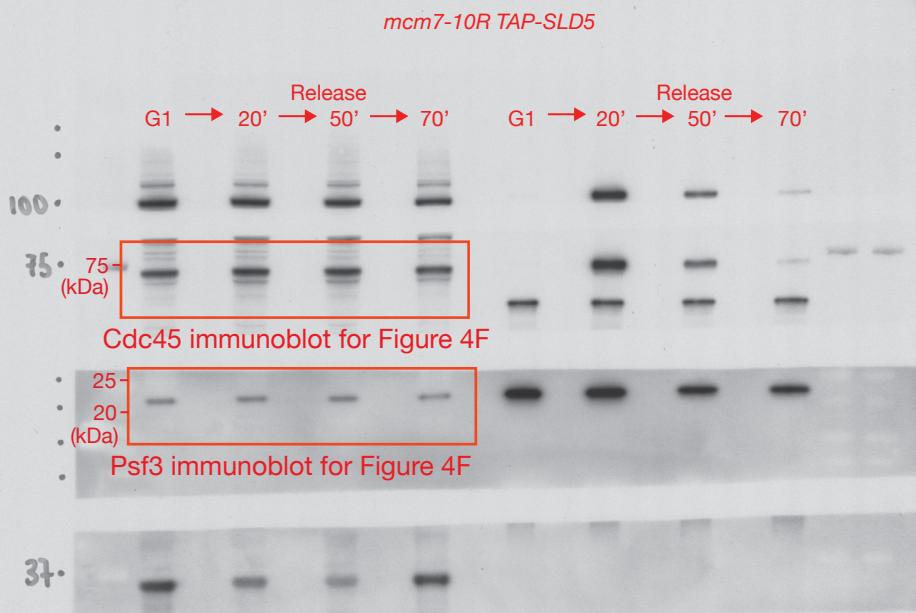

Supplement: Supplementary file 10 — Source data Fig. 4 [file 44318_2024_161_MOESM10_ESM.zip › Source Data_Figure 4/4E-F/Figure 4E-F_Blots_Cdc45-Psf3.pdf]

02/08/22

8min

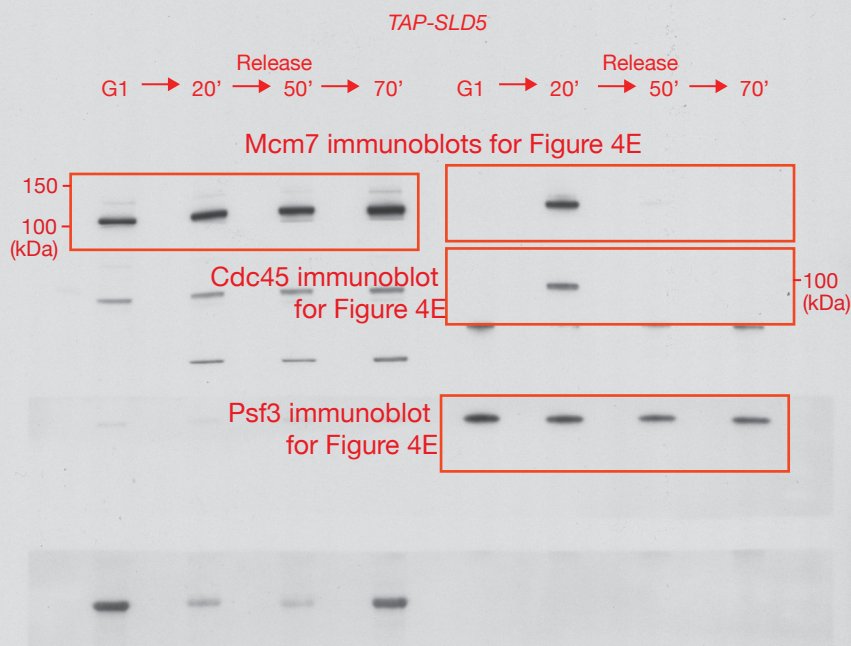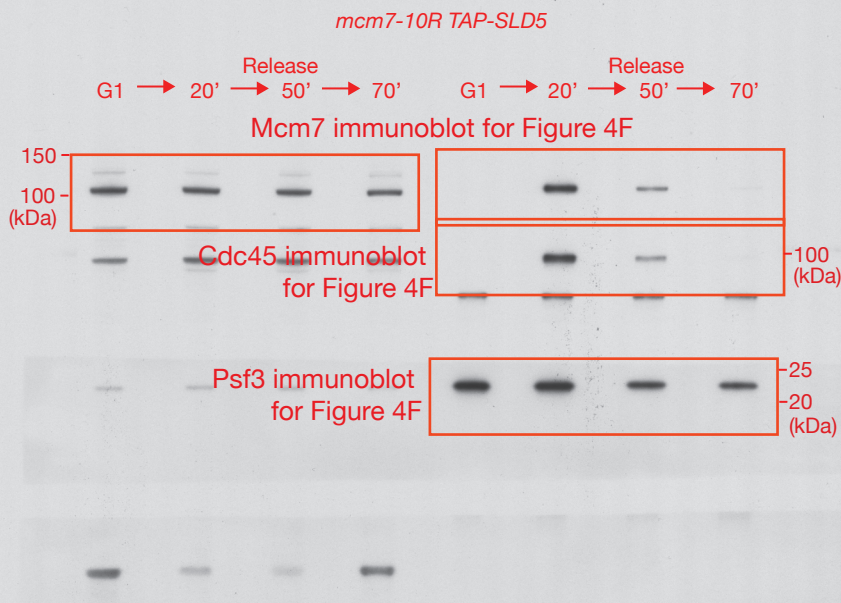

Supplement: Supplementary file 10 — Source data Fig. 4 [file 44318_2024_161_MOESM10_ESM.zip › Source Data_Figure 4/4E-F/Figure 4E-F_Blots_Mcm7-Cdc45-Psf3.pdf]

02/08/22

4 min

### TAP-sld5

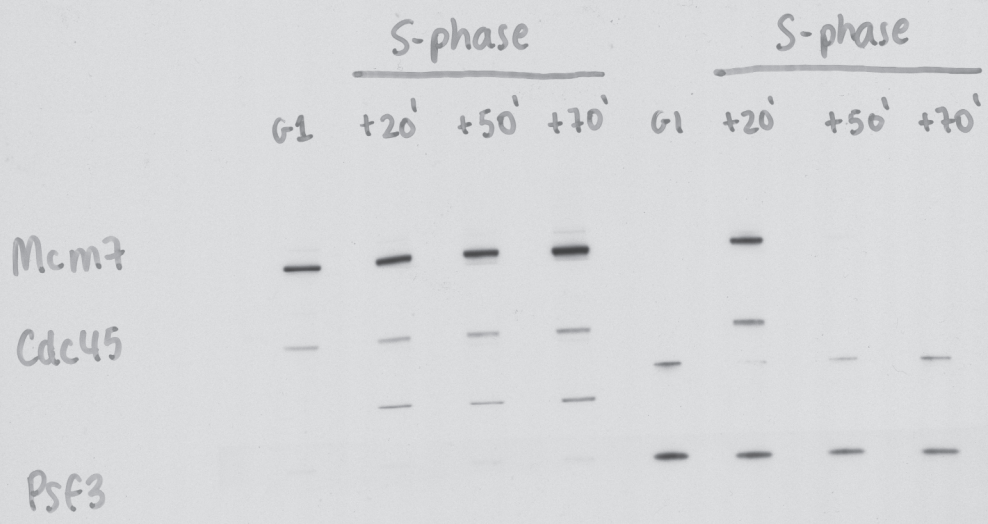

Sic1 immunoblot for Figure 4E

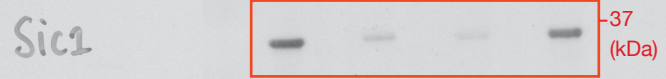

### mcm7-10R

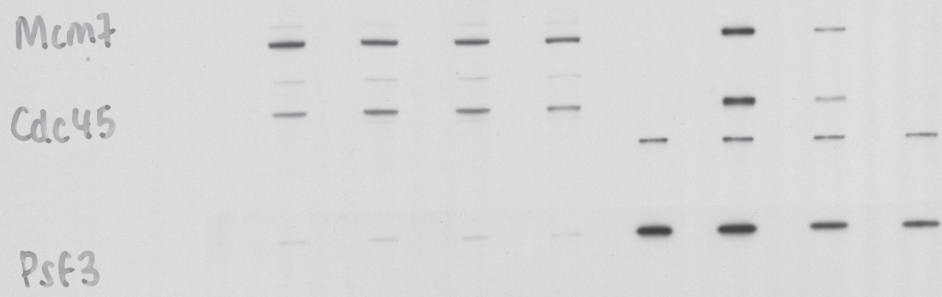

Sic1 immunoblot for Figure 4F

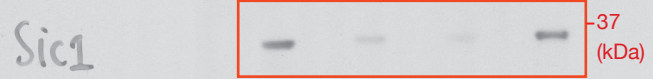

input IP: of TAP-sld5

Supplement: Supplementary file 10 — Source data Fig. 4 [file 44318_2024_161_MOESM10_ESM.zip › Source Data_Figure 4/4E-F/Figure 4E-F_Blot_Sic1.pdf]

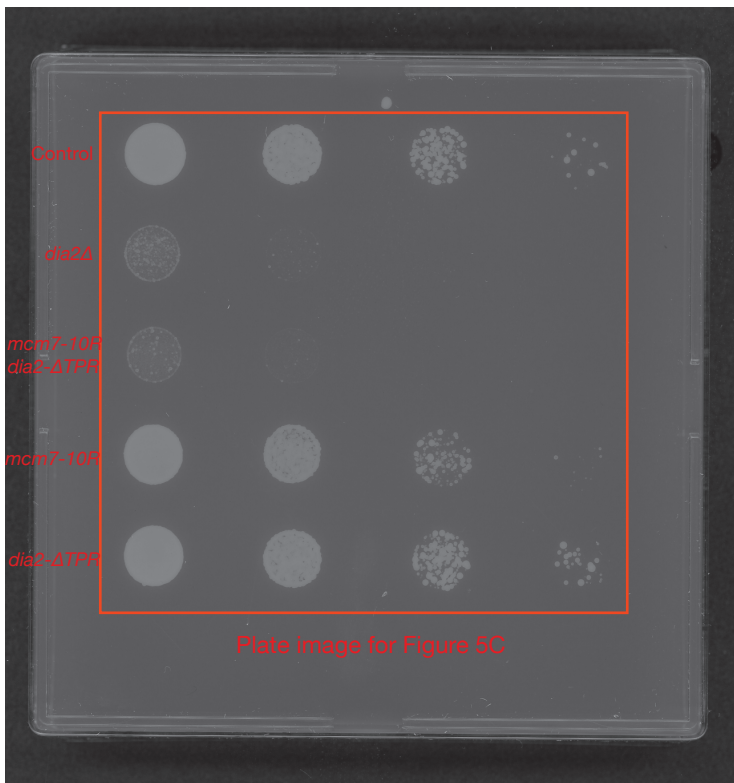

Plate image for Figure 5C

Supplement: Supplementary file 11 — Source data Fig. 5 [file 44318_2024_161_MOESM11_ESM.zip › Source Data_Figure 5/5C/Figure 5C_Plate image_20 degrees.pdf]

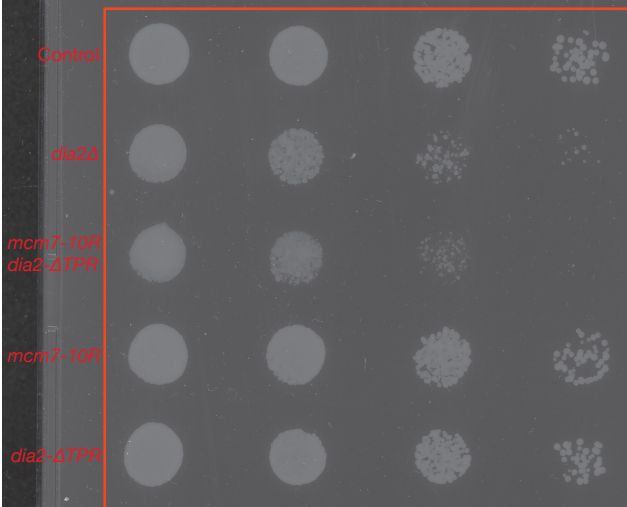

Plate image for Figure 5C

Supplement: Supplementary file 11 — Source data Fig. 5 [file 44318_2024_161_MOESM11_ESM.zip › Source Data_Figure 5/5C/Figure 5C_Plate image_30 degrees.pdf]

Control

*dia2* $\Delta$

*mcm7-10R*

*dia2*- $\Delta$ TPR

*mcm7-10R*  
*dia2*- $\Delta$ TPR

Plate image for Figure 5D

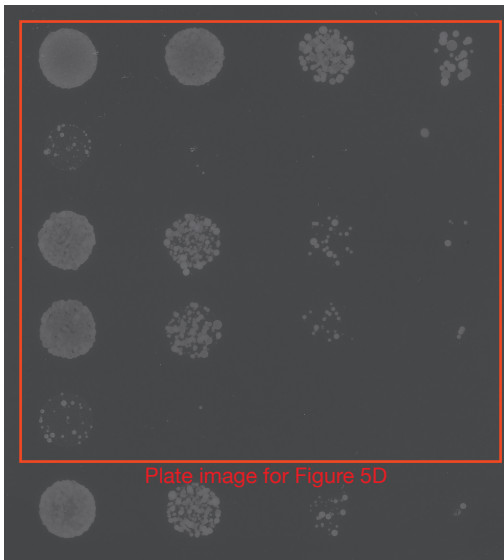

Supplement: Supplementary file 11 — Source data Fig. 5 [file 44318_2024_161_MOESM11_ESM.zip › Source Data_Figure 5/5D/Figure 5D_Plate image_MMS.pdf]

Control

*dia2* $\Delta$

*mcm7-10R*

*dia2- $\Delta$ TPR*

*mcm7-10R*

*dia2- $\Delta$ TPR*

Plate image for Figure 5D

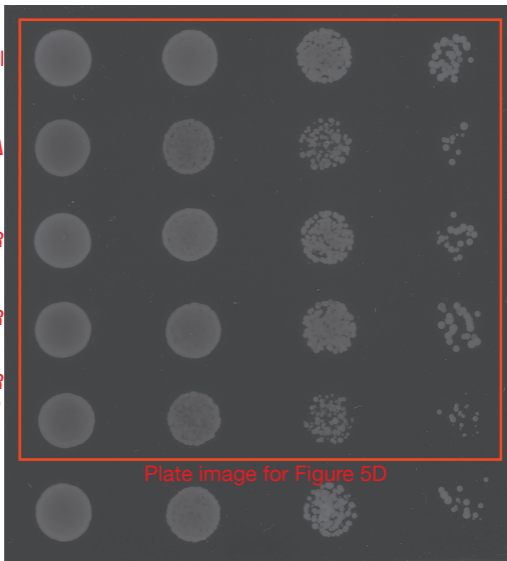

Supplement: Supplementary file 11 — Source data Fig. 5 [file 44318_2024_161_MOESM11_ESM.zip › Source Data_Figure 5/5D/Figure 5D_Plate image_30 degrees.pdf]

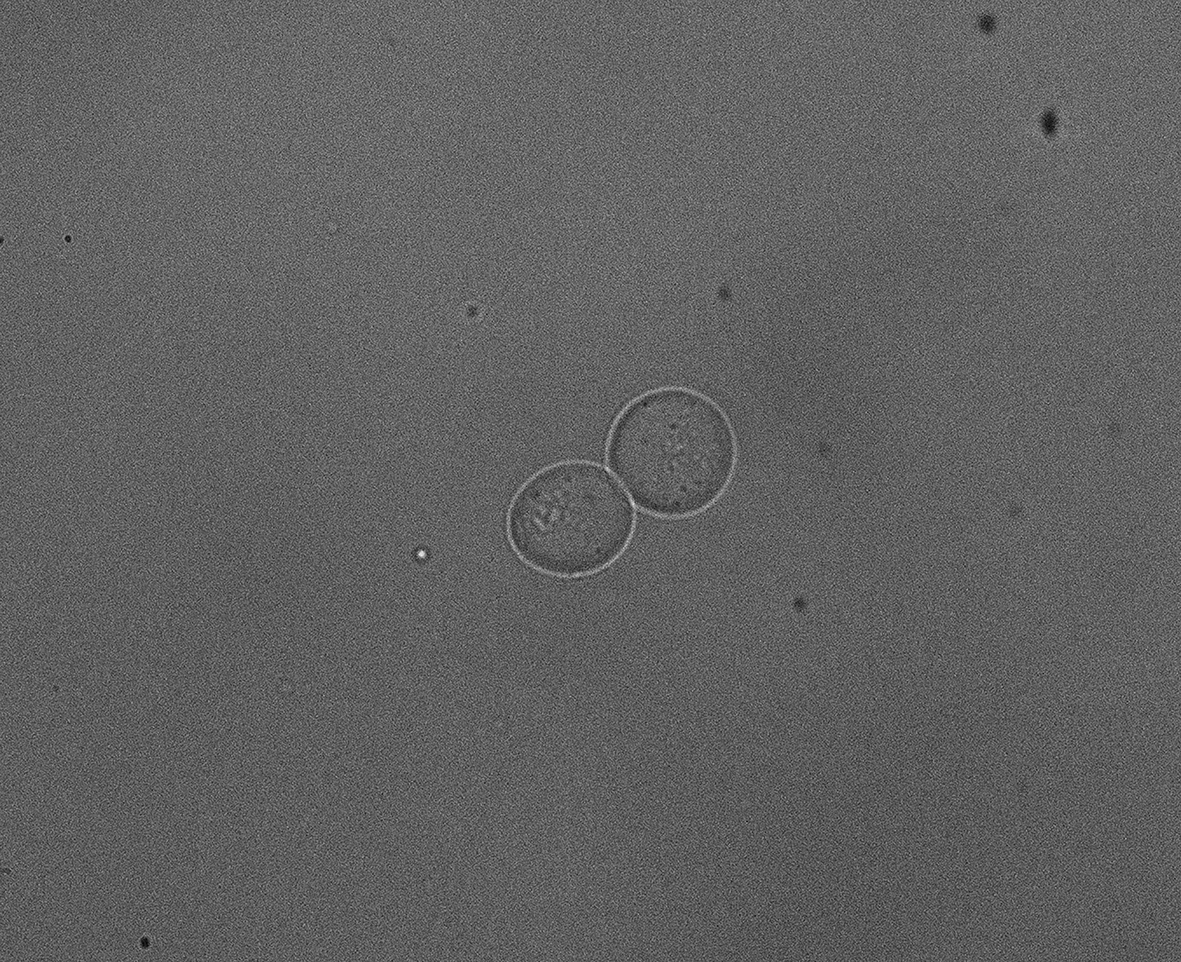

Supplement: Supplementary file 11 — Source data Fig. 5 [file 44318_2024_161_MOESM11_ESM.zip › Source Data_Figure 5/5E/microscopy_bright field.tif]

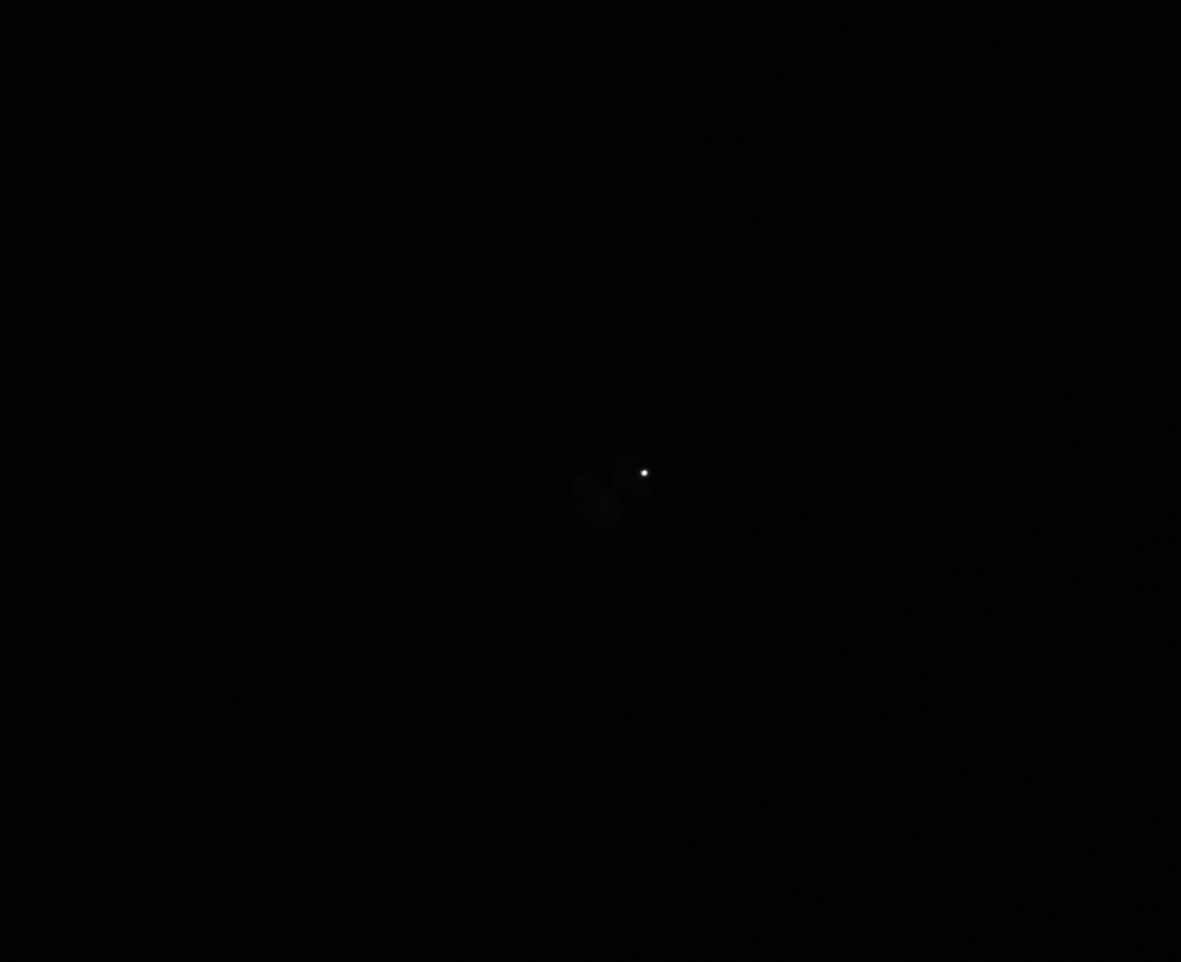

Supplement: Supplementary file 11 — Source data Fig. 5 [file 44318_2024_161_MOESM11_ESM.zip › Source Data_Figure 5/5E/microscopy_Rad52-GFP.tif]

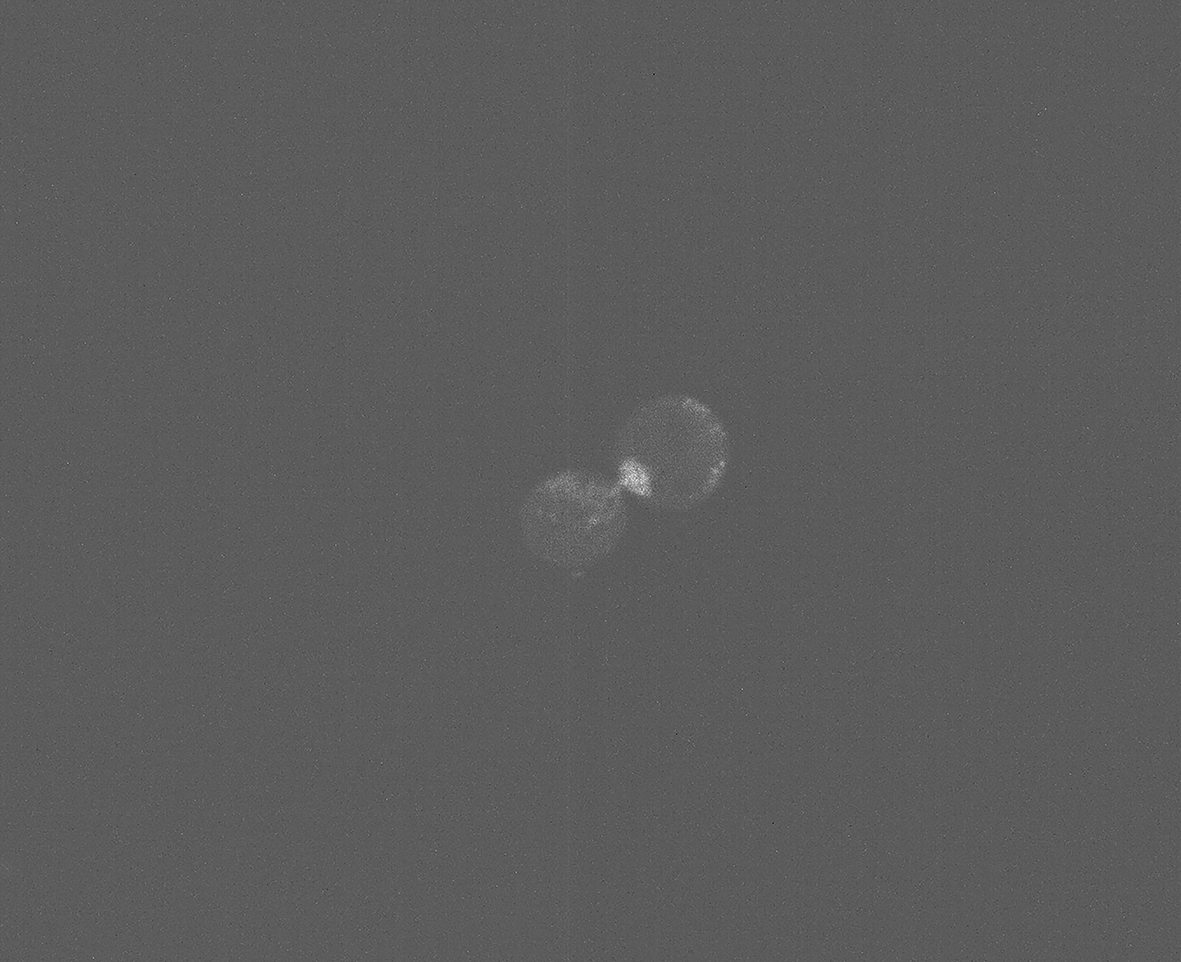

Supplement: Supplementary file 11 — Source data Fig. 5 [file 44318_2024_161_MOESM11_ESM.zip › Source Data_Figure 5/5E/microscopy_DAPI.tif]

02/06/22  
10sec

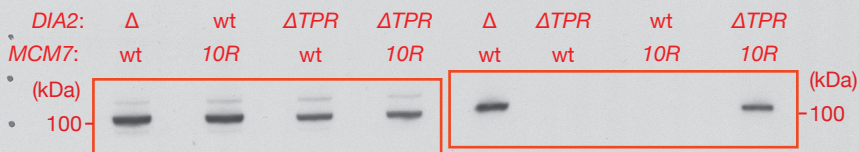

Mcm7 immunoblots for Figure 5B

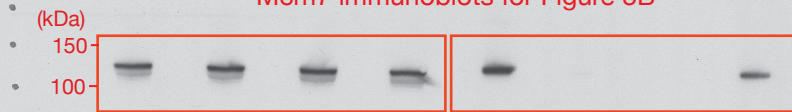

Mcm6 immunoblots for Figure 5B

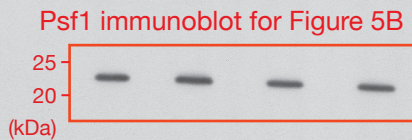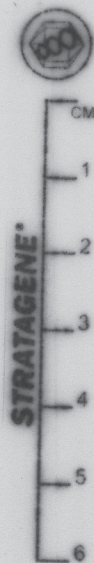

Supplement: Supplementary file 11 — Source data Fig. 5 [file 44318_2024_161_MOESM11_ESM.zip › Source Data_Figure 5/5B/Figure 5B_Blots_Mcm7-Mcm6-Psf1.pdf]

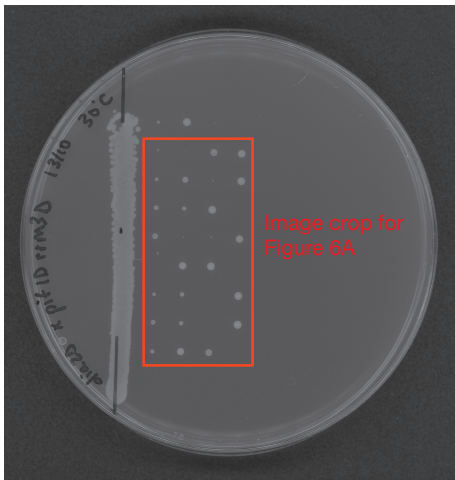

Supplement: Supplementary file 12 — Source data Fig. 6 [file 44318_2024_161_MOESM12_ESM.zip › Source Data_Figure 6/6A/Figure 6A_Plate image.pdf]

19 / 11 / 21

Long

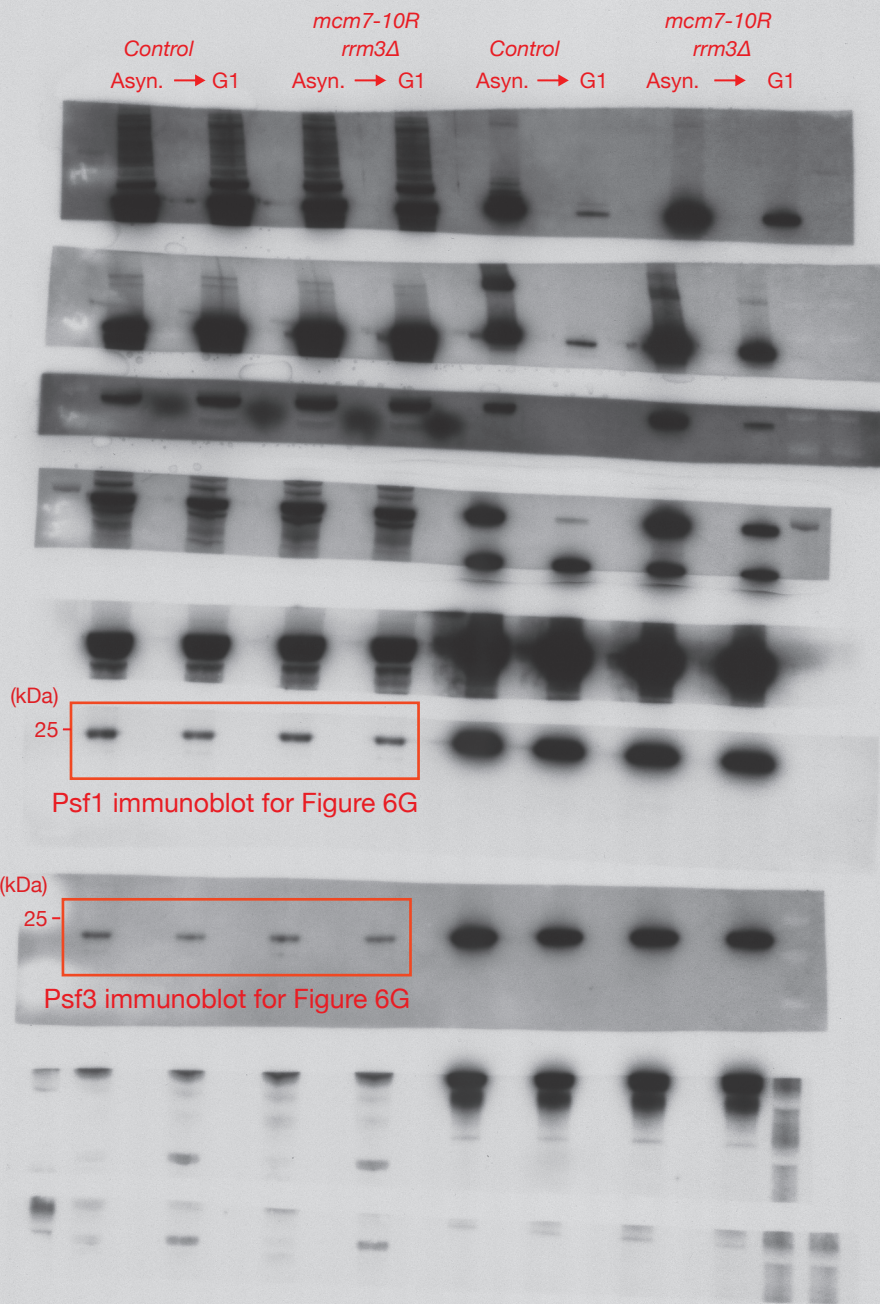

Supplement: Supplementary file 12 — Source data Fig. 6 [file 44318_2024_161_MOESM12_ESM.zip › Source Data_Figure 6/6G/Figure 6G_Blots_Psf1-Psf3.pdf]

19/11/21

20 sec

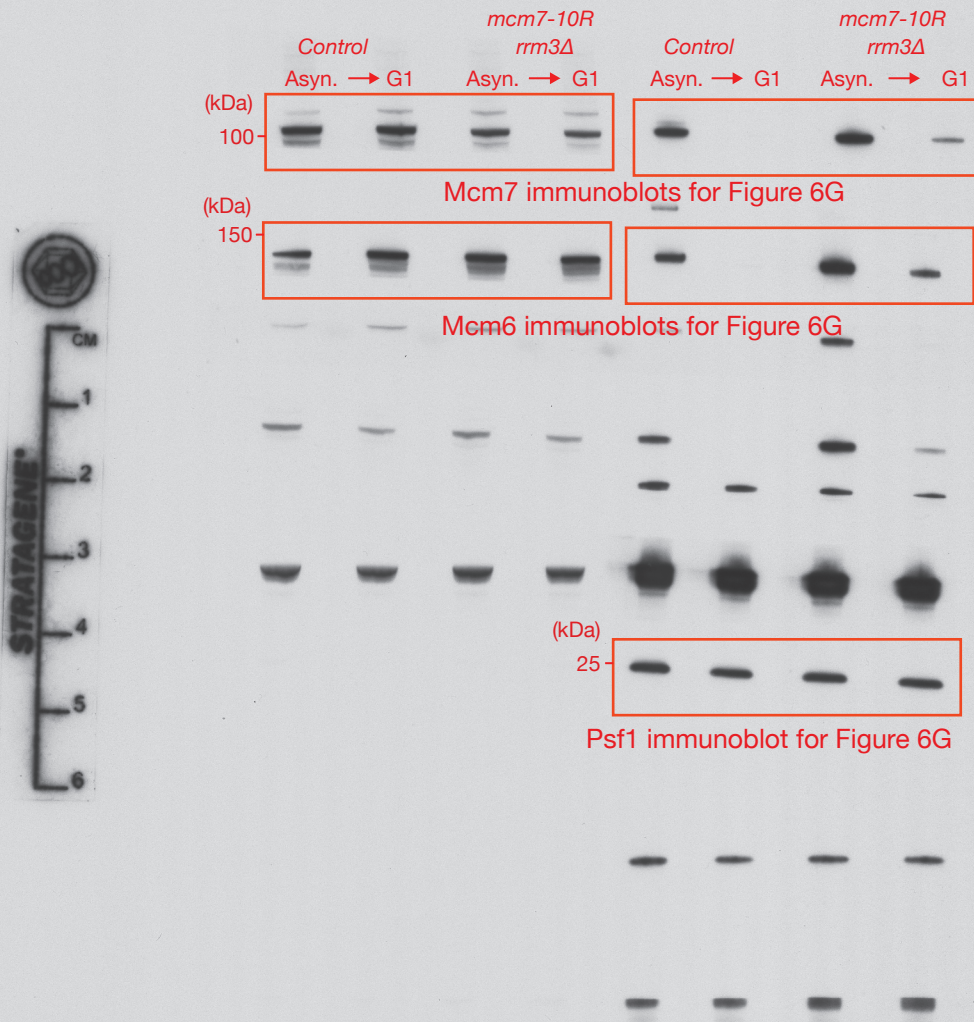

Supplement: Supplementary file 12 — Source data Fig. 6 [file 44318_2024_161_MOESM12_ESM.zip › Source Data_Figure 6/6G/Figure 6G_Blots_Mcm7-Mcm6-Psf1.pdf]

19/11/21

1min

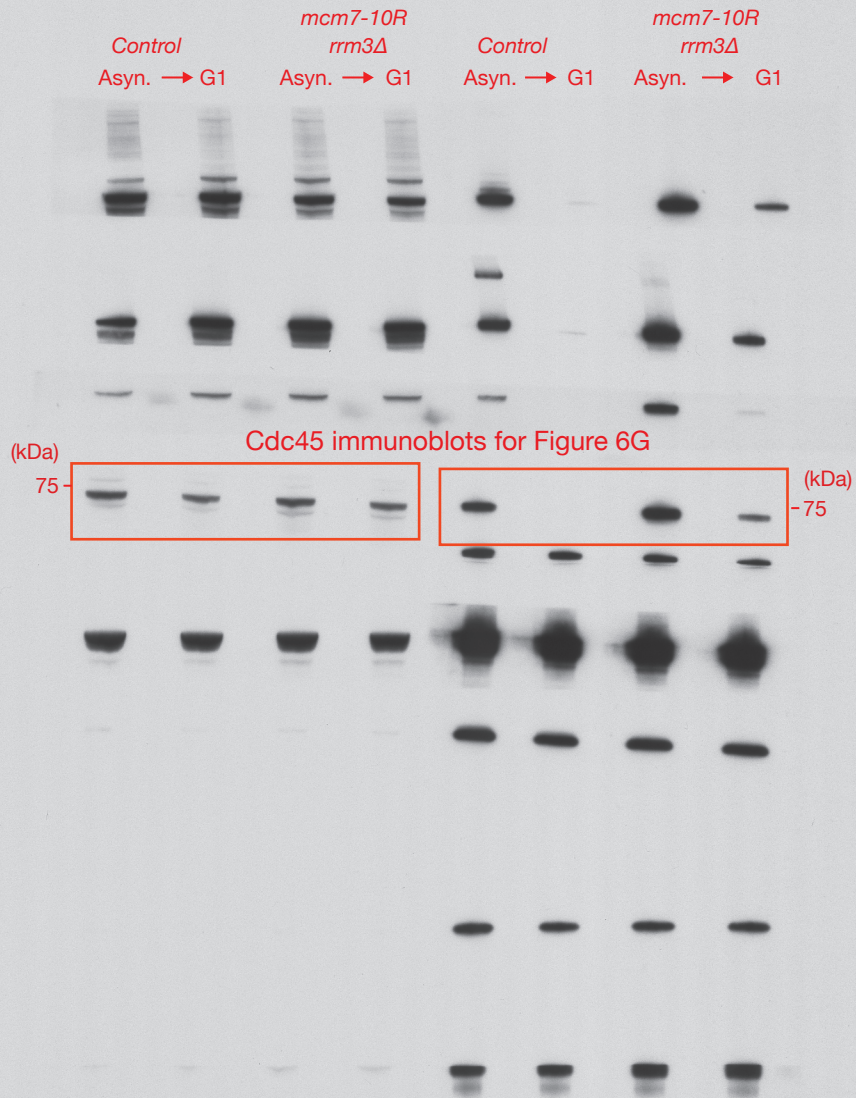

Supplement: Supplementary file 12 — Source data Fig. 6 [file 44318_2024_161_MOESM12_ESM.zip › Source Data_Figure 6/6G/Figure 6G_Blots_Cdc45.pdf]

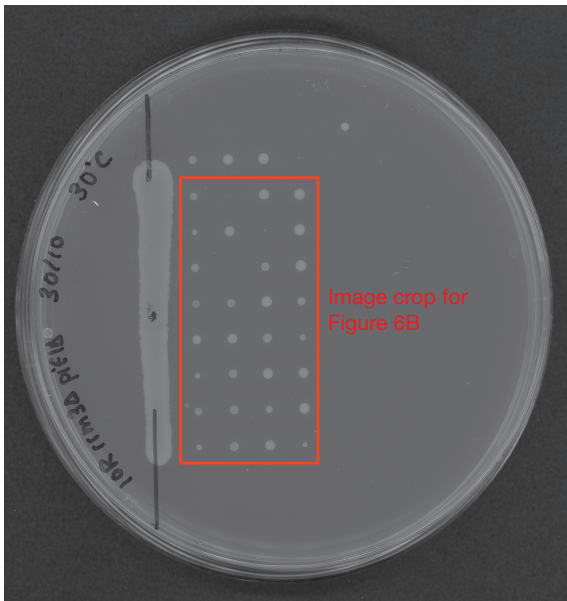

Supplement: Supplementary file 12 — Source data Fig. 6 [file 44318_2024_161_MOESM12_ESM.zip › Source Data_Figure 6/6B/Figure 6B_Plate image.pdf]

10/03/21

1min

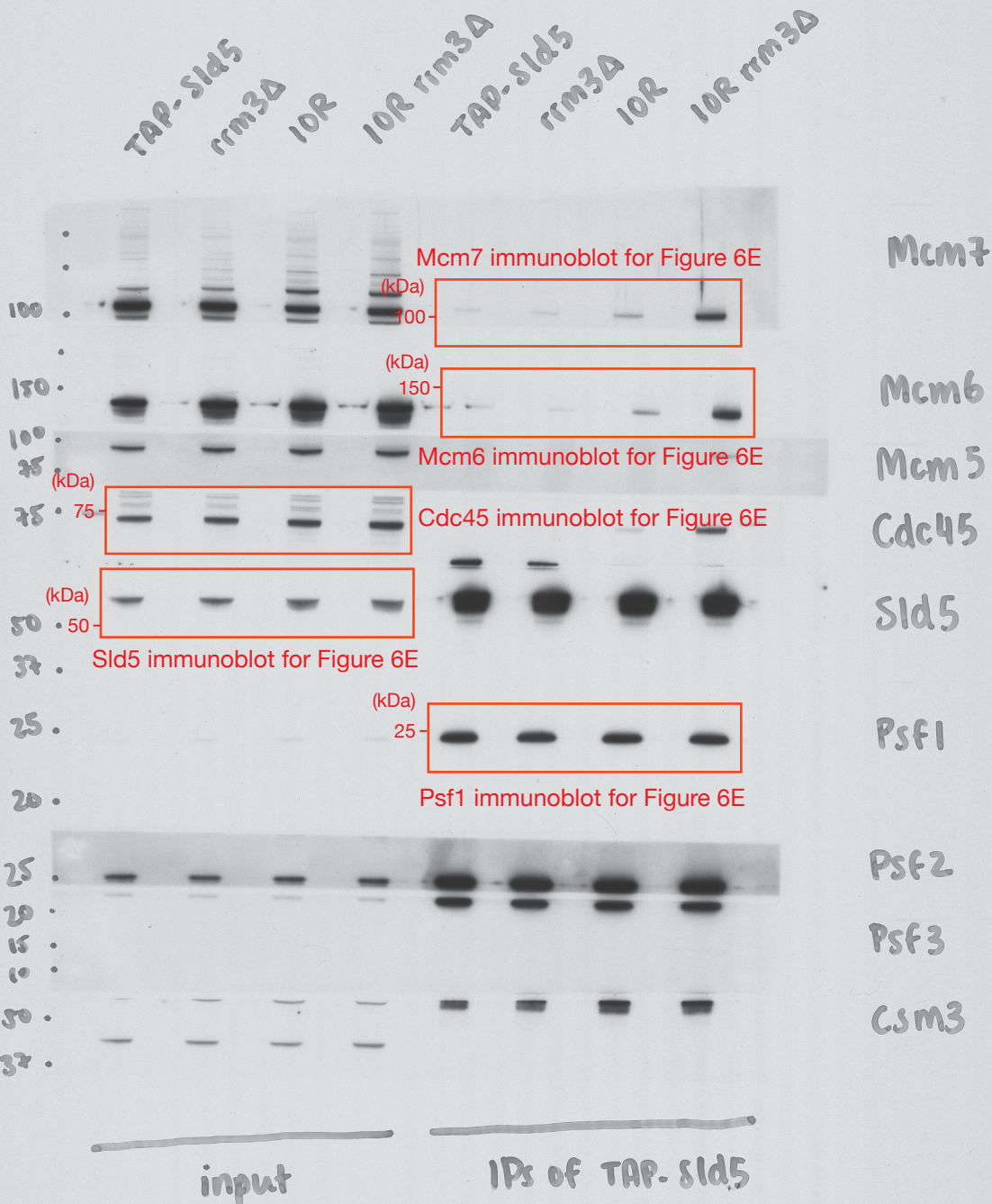

Supplement: Supplementary file 12 — Source data Fig. 6 [file 44318_2024_161_MOESM12_ESM.zip › Source Data_Figure 6/6E/Figure 6E_Blots_Mcm7-Mcm6-Cdc45-Psf1-Sld5.pdf]

10/03/21

5min

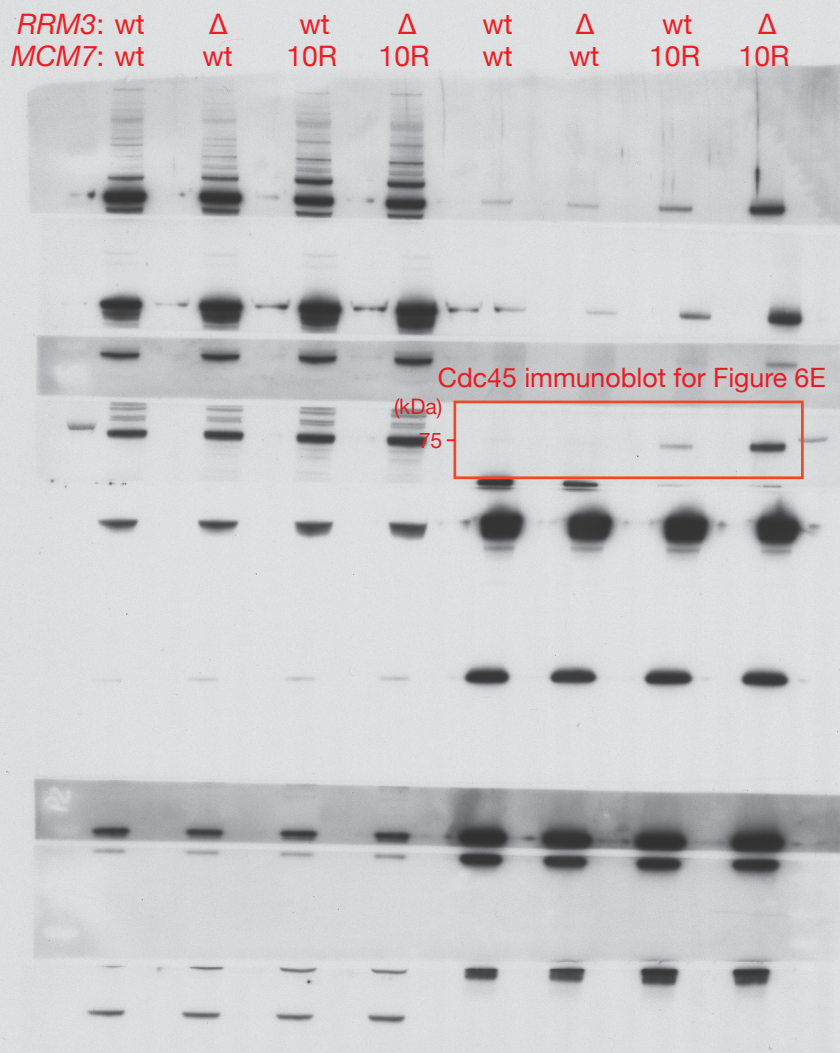

Supplement: Supplementary file 12 — Source data Fig. 6 [file 44318_2024_161_MOESM12_ESM.zip › Source Data_Figure 6/6E/Figure 6E_Blot_Cdc45.pdf]

10/03/21

30 min

RRM3: wt     $\Delta$     wt     $\Delta$     wt     $\Delta$     wt     $\Delta$   
MCM7: wt    wt    10R    10R    wt    wt    10R    10R

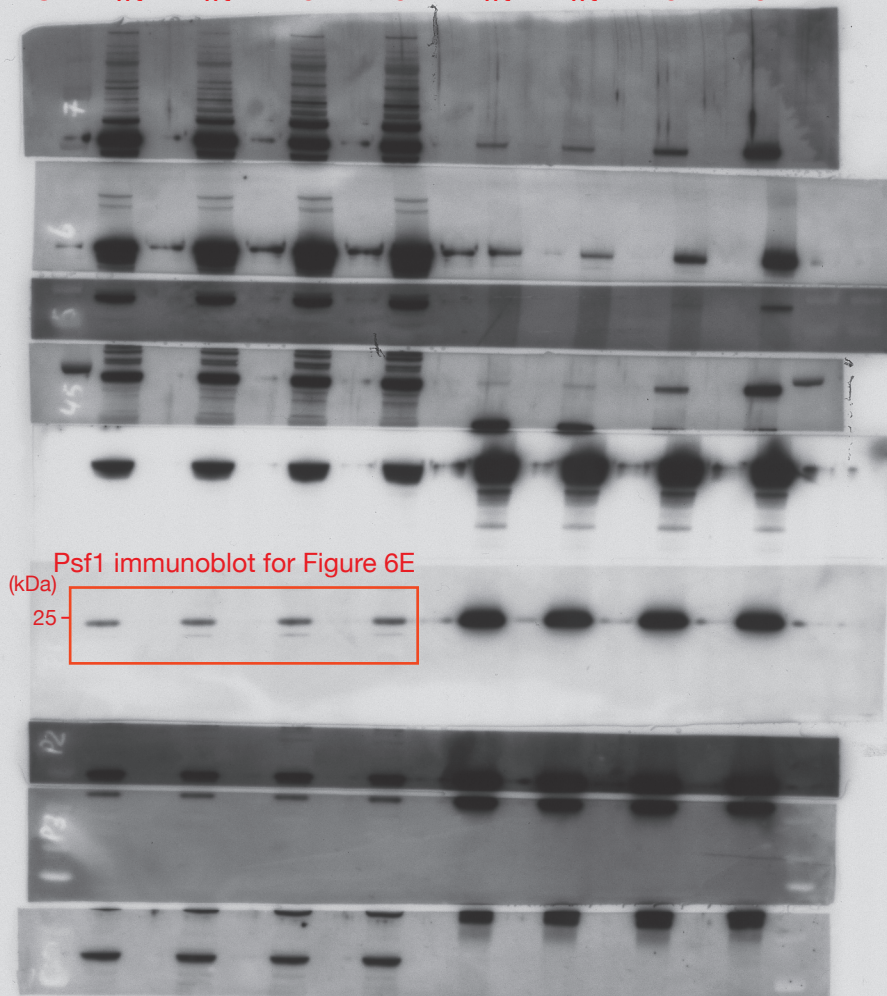

Supplement: Supplementary file 12 — Source data Fig. 6 [file 44318_2024_161_MOESM12_ESM.zip › Source Data_Figure 6/6E/Figure 6E_Blot_Psf1.pdf]

29/03/22  
20sec

| G1-phase: | 1st | 1st | 2nd | 2nd | 1st | 1st | 2nd | 2nd |
|-----------|-----|-----|-----|-----|-----|-----|-----|-----|
| GAL-RRM3: | OFF | ON  | OFF | ON  | OFF | ON  | OFF | ON  |

Mcm6 immunoblot for Figure 7C

150  
(kDa)

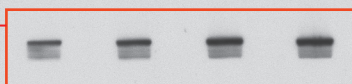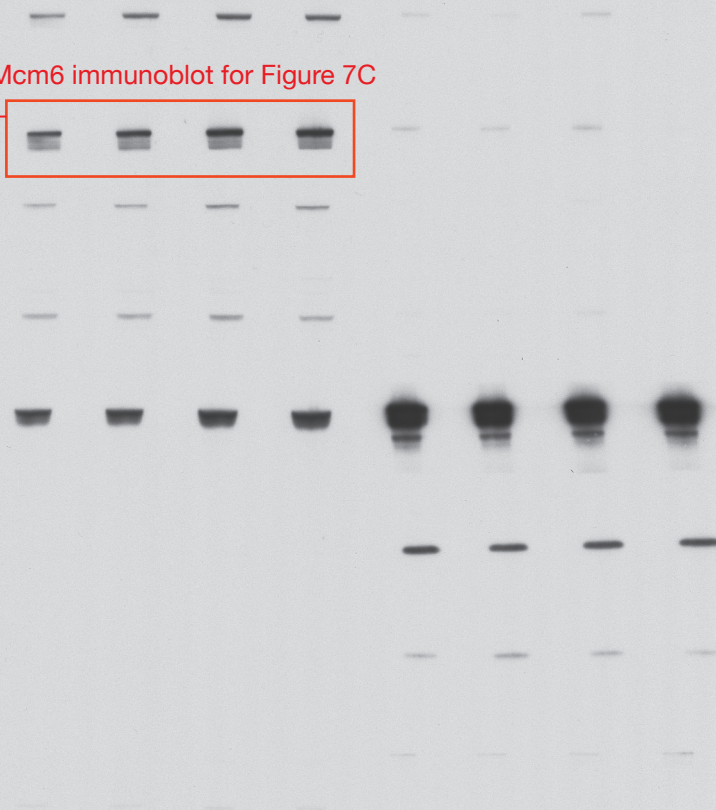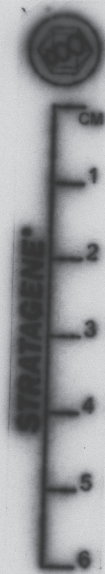

Supplement: Supplementary file 13 — Source data Fig. 7 [file 44318_2024_161_MOESM13_ESM.zip › Source Data_Figure 7/7C/Figure 7C_Blot_Mcm6.pdf]

29/03/22  
18 min

|           |     |     |     |     |     |     |     |     |
|-----------|-----|-----|-----|-----|-----|-----|-----|-----|
| G1-phase: | 1st | 1st | 2nd | 2nd | 1st | 1st | 2nd | 2nd |
| GAL-RRM3: | OFF | ON  | OFF | ON  | OFF | ON  | OFF | ON  |

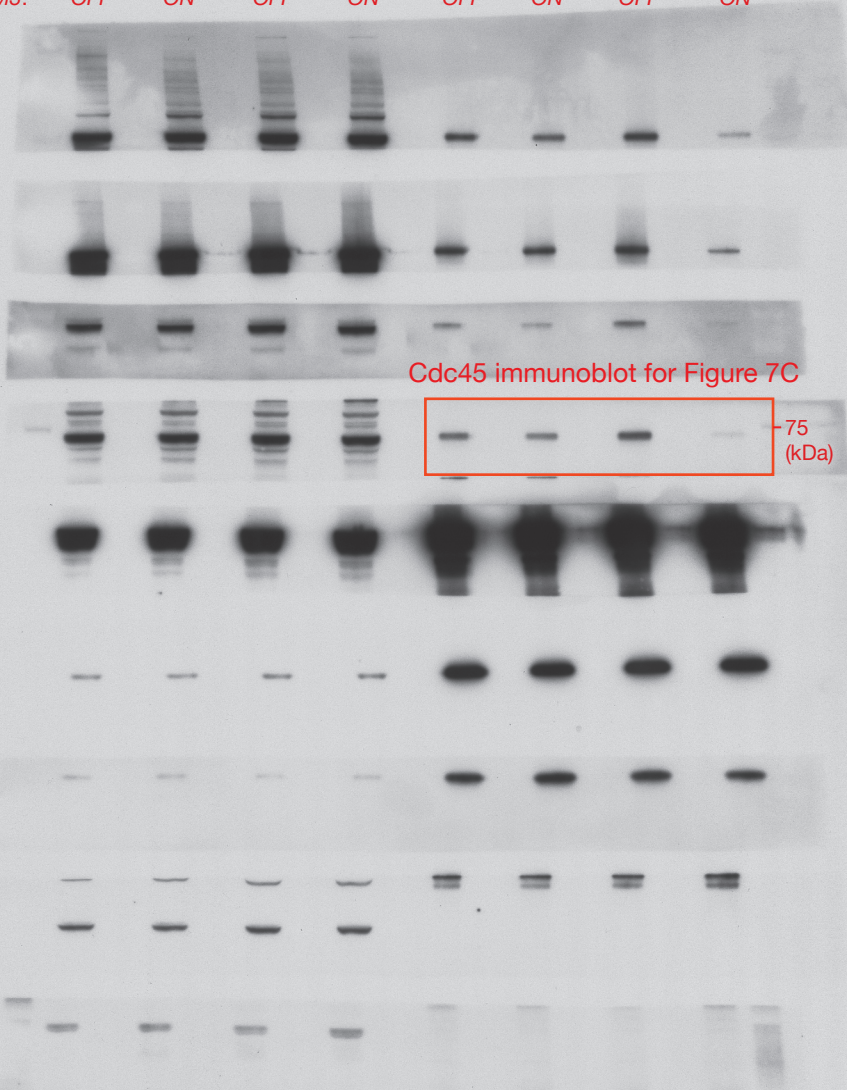

Supplement: Supplementary file 13 — Source data Fig. 7 [file 44318_2024_161_MOESM13_ESM.zip › Source Data_Figure 7/7C/Figure 7C_Blot_Cdc45.pdf]

29/03/22  
1h

| G1-phase: | 1st | 1st | 2nd | 2nd | 1st | 1st | 2nd | 2nd |
|-----------|-----|-----|-----|-----|-----|-----|-----|-----|
| GAL-RRM3: | OFF | ON  | OFF | ON  | OFF | ON  | OFF | ON  |

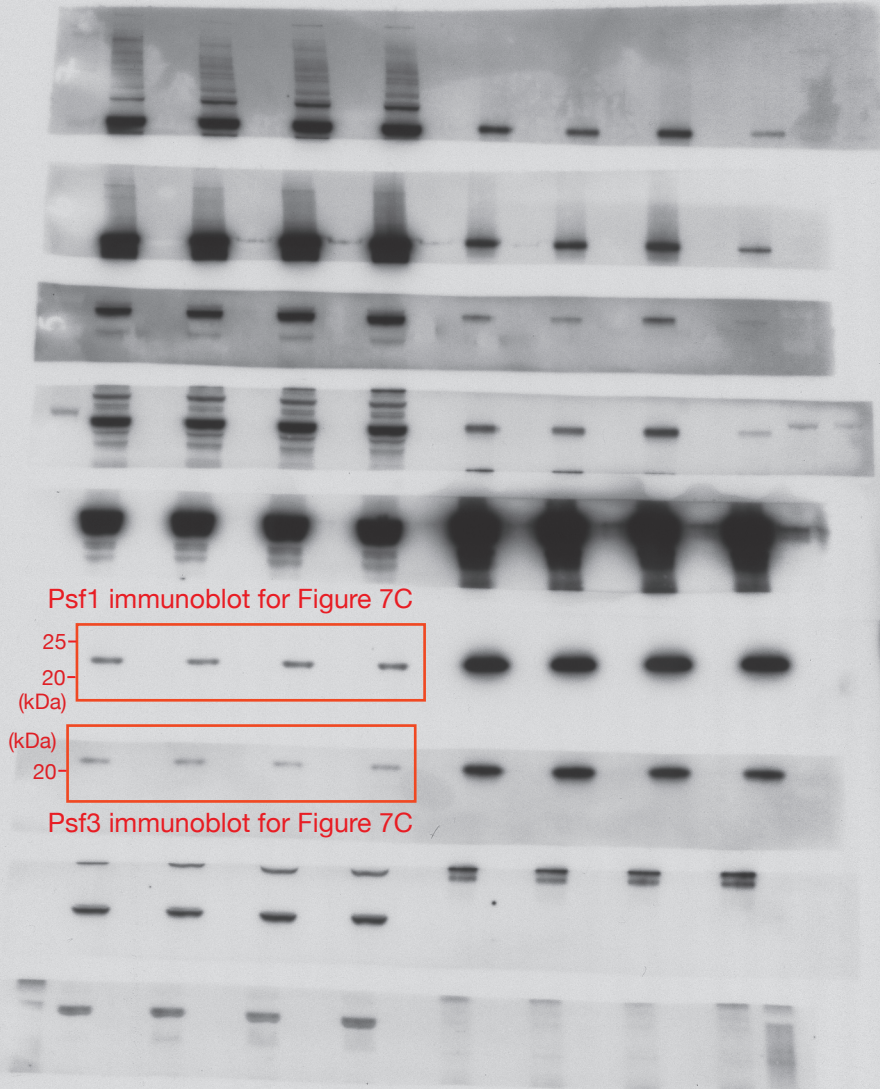

Supplement: Supplementary file 13 — Source data Fig. 7 [file 44318_2024_161_MOESM13_ESM.zip › Source Data_Figure 7/7C/Figure 7C_Blots_Psf1-Psf3.pdf]
