# Supplementary material for: CMG helicase disassembly is essential and driven by two pathways in budding yeast
Source: EMBO J. 2024 Jul 22;43(18):2. doi: 10.1038/s44318-024-00161-x (PMC11405719; doi:10.1038/s44318-024-00161-x)
Supplement: Supplementary file 8 — Source data Fig. 2 [file 44318_2024_161_MOESM8_ESM.zip › Source Data_Figure 2/2C-E/Figures 2 C-E_Blots_Mcm7-Cdc45-Mcm3-Mcm4.pdf]

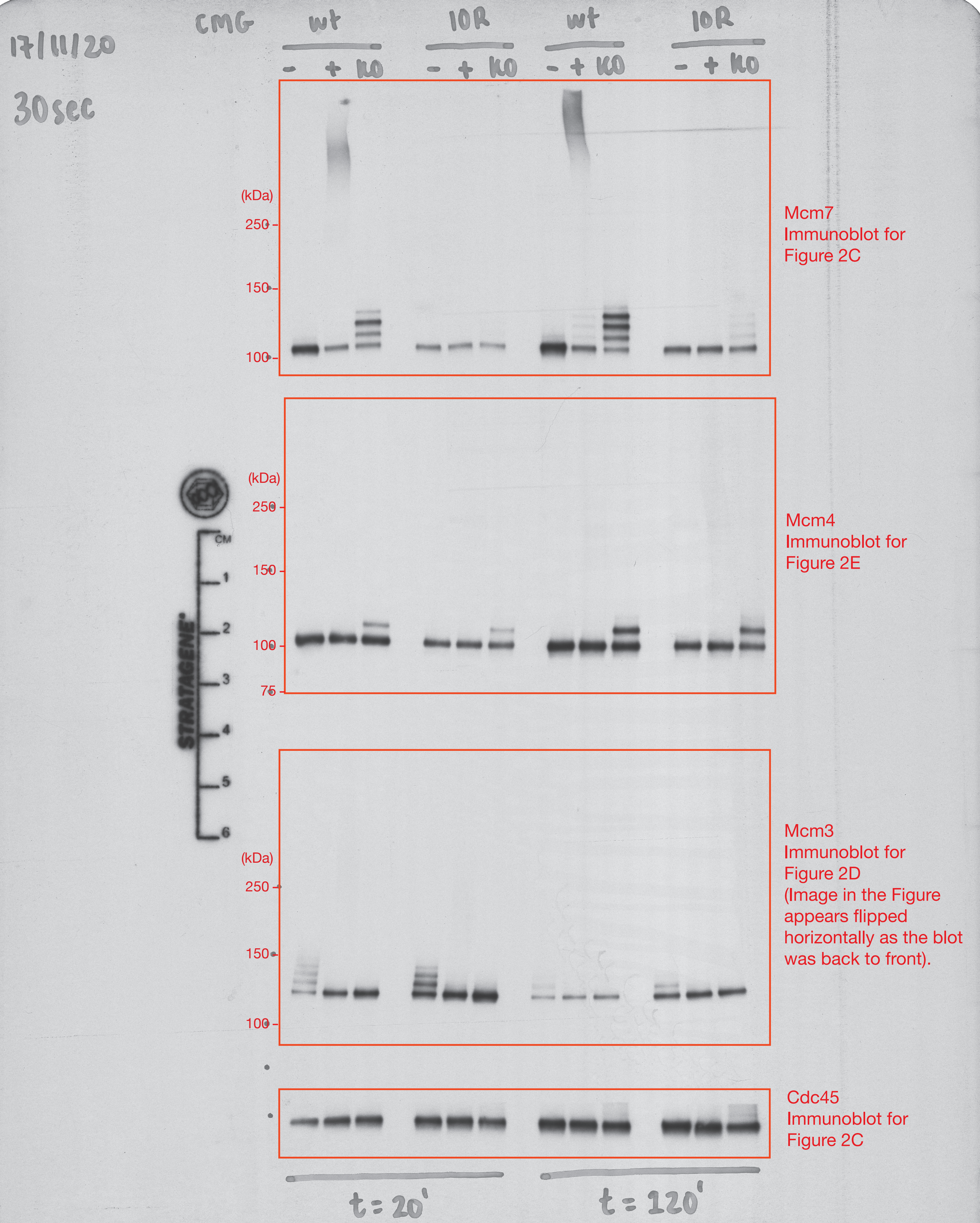
17/11/20
30sec
CMG
wt
10R
wt
10R
- + KO
- + KO
- + KO
- + KO
(kDa)
250
150
100
Mcm7
Immunoblot for
Figure 2C
(kDa)
250
150
100
75
Mcm4
Immunoblot for
Figure 2E
STRATAGENE
CM
1
2
3
4
5
6
(kDa)
250
150
100
Mcm3
Immunoblot for
Figure 2D
(Image in the Figure
appears flipped
horizontally as the blot
was back to front).
Cdc45
Immunoblot for
Figure 2C
t = 20'
t = 120'
