# Supplementary material for: CMG helicase disassembly is essential and driven by two pathways in budding yeast
Source: EMBO J. 2024 Jul 22;43(18):2. doi: 10.1038/s44318-024-00161-x (PMC11405719; doi:10.1038/s44318-024-00161-x)
Supplement: Supplementary file 9 — Source data Fig. 3 [file 44318_2024_161_MOESM9_ESM.zip › Source Data_Figure 3/3B/Figure 3B_Blot_Mcm6-Cdc45- Sld5.pdf]

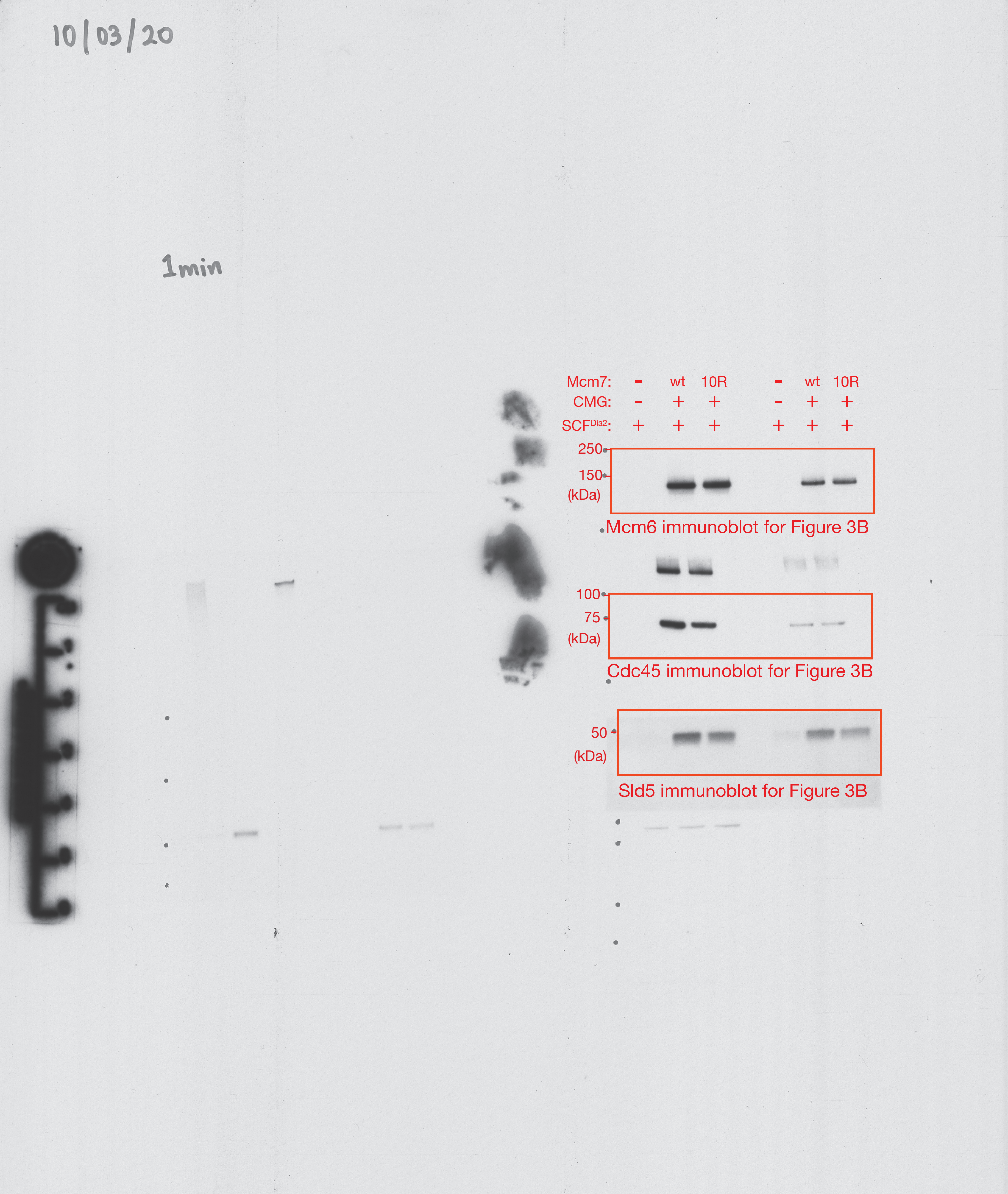
10/03/20
1min
Mcm7: - wt 10R - wt 10R
CMG: - + + - + +
SCFDia2: + + + + + +
250
150
(kDa)
Mcm6 immunoblot for Figure 3B
100
75
(kDa)
Cdc45 immunoblot for Figure 3B
50
(kDa)
Sld5 immunoblot for Figure 3B
