# Supplementary material for: CMG helicase disassembly is essential and driven by two pathways in budding yeast
Source: EMBO J. 2024 Jul 22;43(18):2. doi: 10.1038/s44318-024-00161-x (PMC11405719; doi:10.1038/s44318-024-00161-x)
Supplement: Supplementary file 9 — Source data Fig. 3 [file 44318_2024_161_MOESM9_ESM.zip › Source Data_Figure 3/3D/Figure 3D_Blots_Mcm3-Psf1-Sld5.pdf]

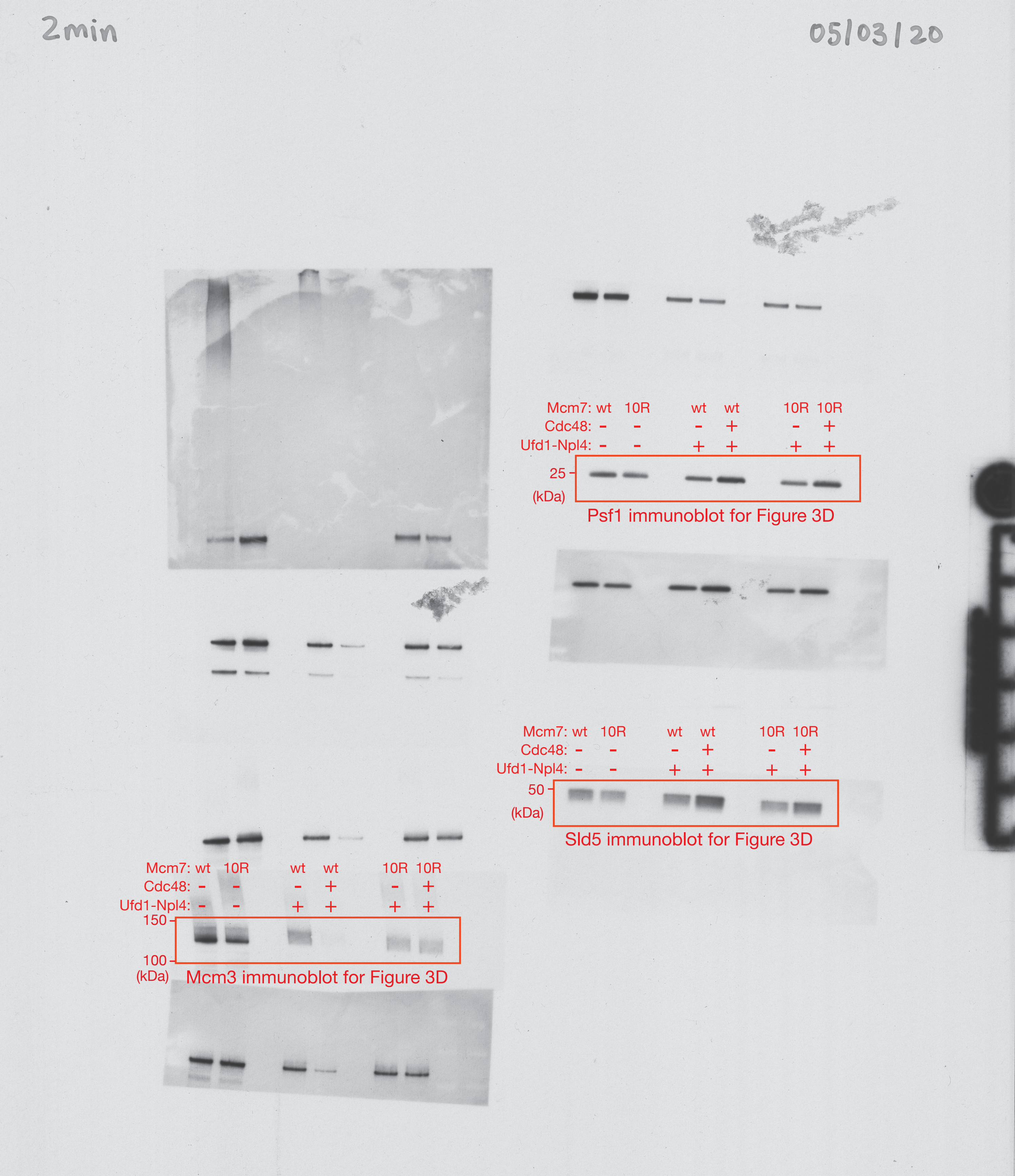
2min
05/03/20
Mcm7: wt 10R wt wt 10R 10R
Cdc48: - - - + - +
Ufd1-Npl4: - - + + + +
25
(kDa)
Psf1 immunoblot for Figure 3D
Mcm7: wt 10R wt wt 10R 10R
Cdc48: - - - + - +
Ufd1-Npl4: - - + + + +
50
(kDa)
Sld5 immunoblot for Figure 3D
Mcm7: wt 10R wt wt 10R 10R
Cdc48: - - - + - +
Ufd1-Npl4: - - + + + +
150
100
(kDa)
Mcm3 immunoblot for Figure 3D
