# Supplementary material for: CMG helicase disassembly is essential and driven by two pathways in budding yeast
Source: EMBO J. 2024 Jul 22;43(18):2. doi: 10.1038/s44318-024-00161-x (PMC11405719; doi:10.1038/s44318-024-00161-x)
Supplement: Supplementary file 10 — Source data Fig. 4 [file 44318_2024_161_MOESM10_ESM.zip › Source Data_Figure 4/4C/Figure 4C_Blots_Mcm7-Cdc45-Psf1-Psf2.pdf]

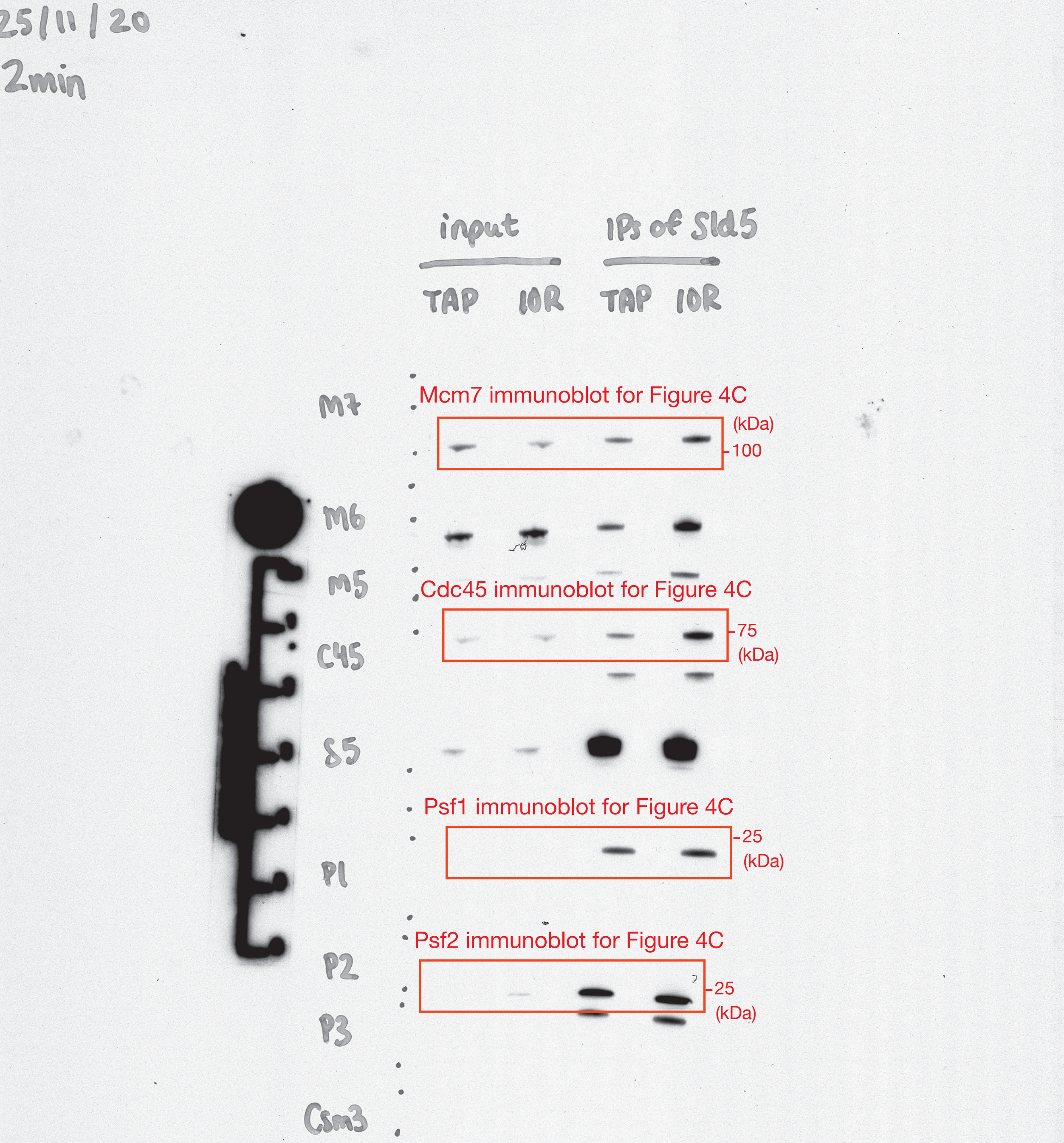
25/11/20
2min
input
IPs of Sld5
TAP
10R
TAP
10R
M7
Mcm7 immunoblot for Figure 4C
(kDa)
100
M6
M5
Cdc45 immunoblot for Figure 4C
75
(kDa)
C45
S5
Psf1 immunoblot for Figure 4C
25
(kDa)
P1
Psf2 immunoblot for Figure 4C
P2
25
(kDa)
P3
Csm3
