# Supplementary material for: CMG helicase disassembly is essential and driven by two pathways in budding yeast
Source: EMBO J. 2024 Jul 22;43(18):2. doi: 10.1038/s44318-024-00161-x (PMC11405719; doi:10.1038/s44318-024-00161-x)
Supplement: Supplementary file 10 — Source data Fig. 4 [file 44318_2024_161_MOESM10_ESM.zip › Source Data_Figure 4/4E-F/Figure 4E-F_Blot_Psf2.pdf]

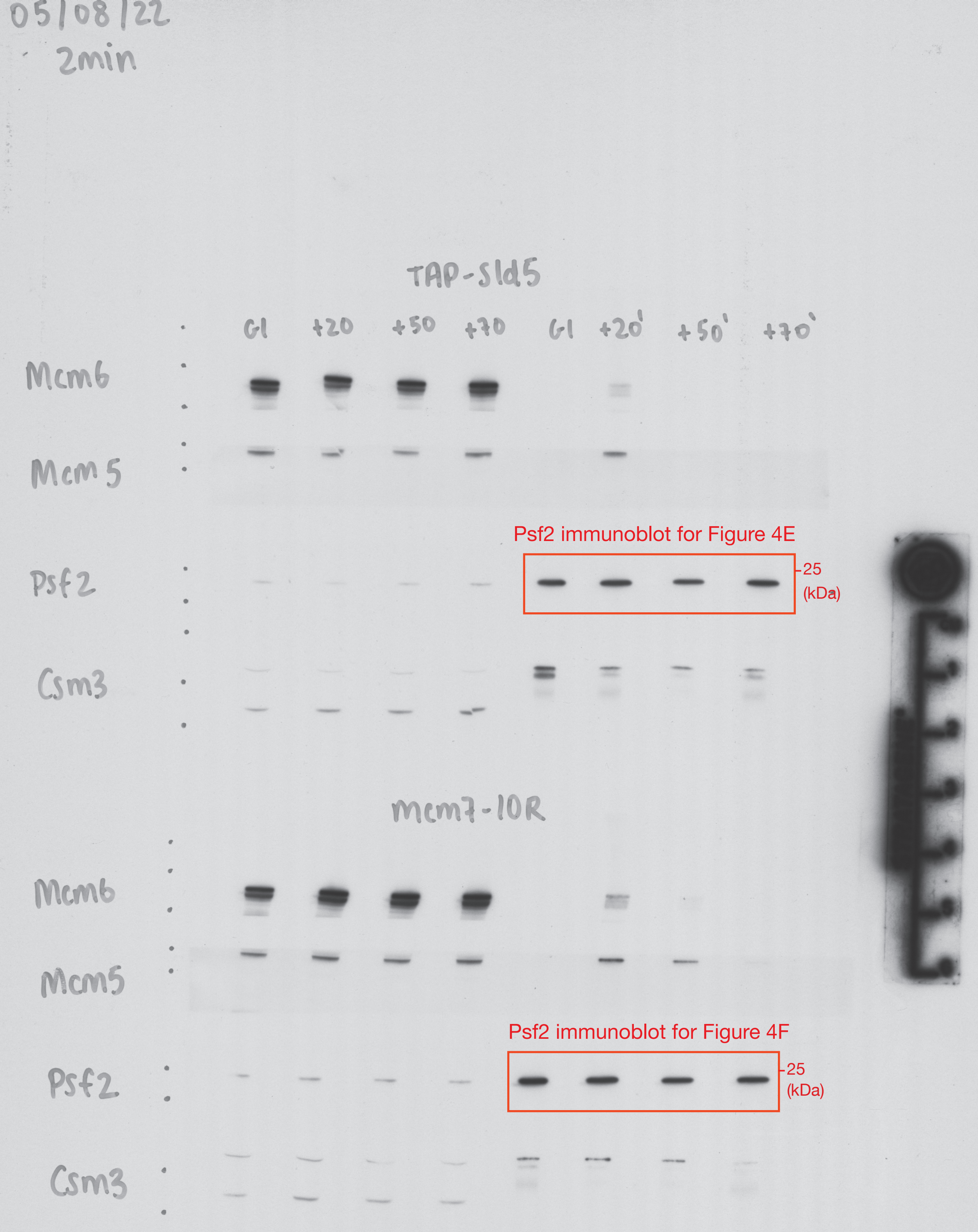
05/08/22
2min
TAP-Sld5
G1 +20 +50 +70 G1 +20' +50' +70'
Mcm6
Mcm5
Psf2 immunoblot for Figure 4E
Psf2
25
(kDa)
Csm3
Mcm7-10R
Mcm6
Mcm5
Psf2 immunoblot for Figure 4F
Psf2
25
(kDa)
Csm3
