# Supplementary material for: CMG helicase disassembly is essential and driven by two pathways in budding yeast
Source: EMBO J. 2024 Jul 22;43(18):2. doi: 10.1038/s44318-024-00161-x (PMC11405719; doi:10.1038/s44318-024-00161-x)
Supplement: Supplementary file 11 — Source data Fig. 5 [file 44318_2024_161_MOESM11_ESM.zip › Source Data_Figure 5/5E/READ ME.pdf]

Bright Field

DAPI (DNA)

Rad52-GFP

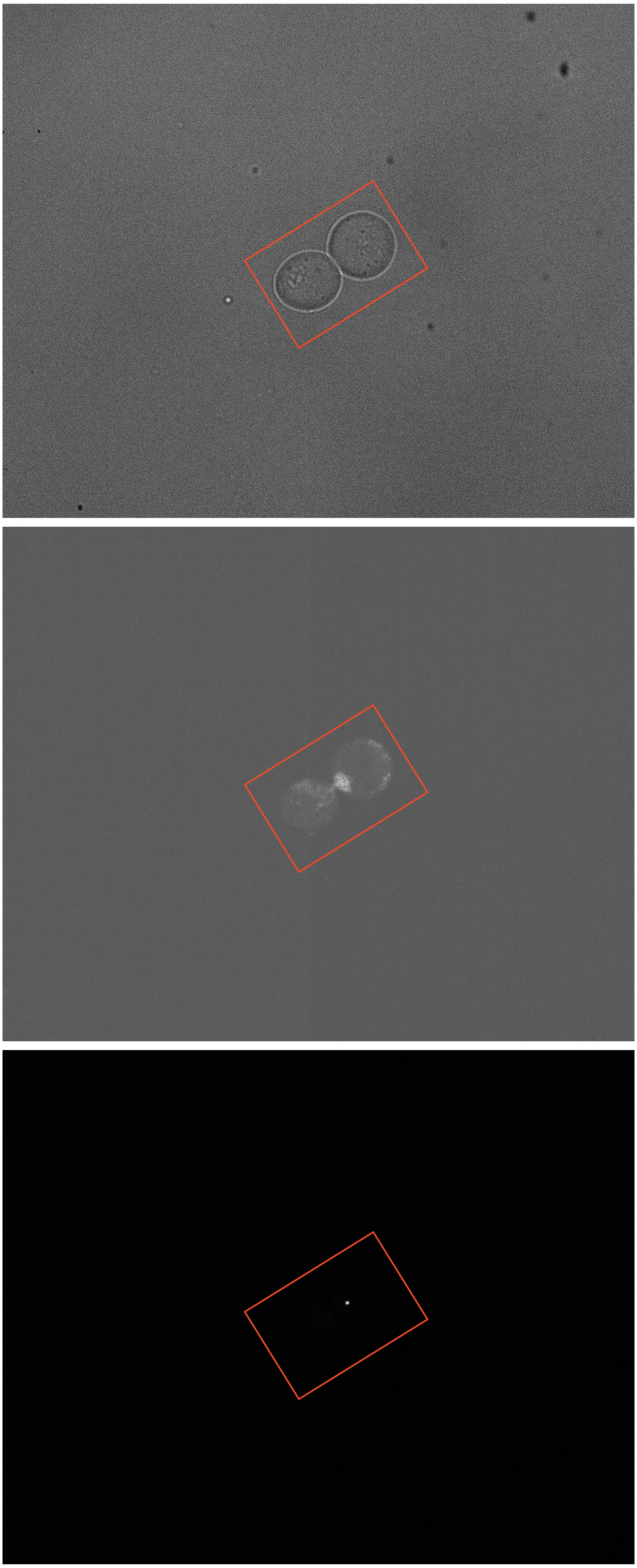

Source data for Figure 5E.
Micrographs with red boxes indicating the areas cropped in the Figure.
