# Supplementary material for: CMG helicase disassembly is essential and driven by two pathways in budding yeast
Source: EMBO J. 2024 Jul 22;43(18):2. doi: 10.1038/s44318-024-00161-x (PMC11405719; doi:10.1038/s44318-024-00161-x)
Supplement: Supplementary file 11 — Source data Fig. 5 [file 44318_2024_161_MOESM11_ESM.zip › Source Data_Figure 5/5B/Figure 5B_Blots Psf1.pdf]

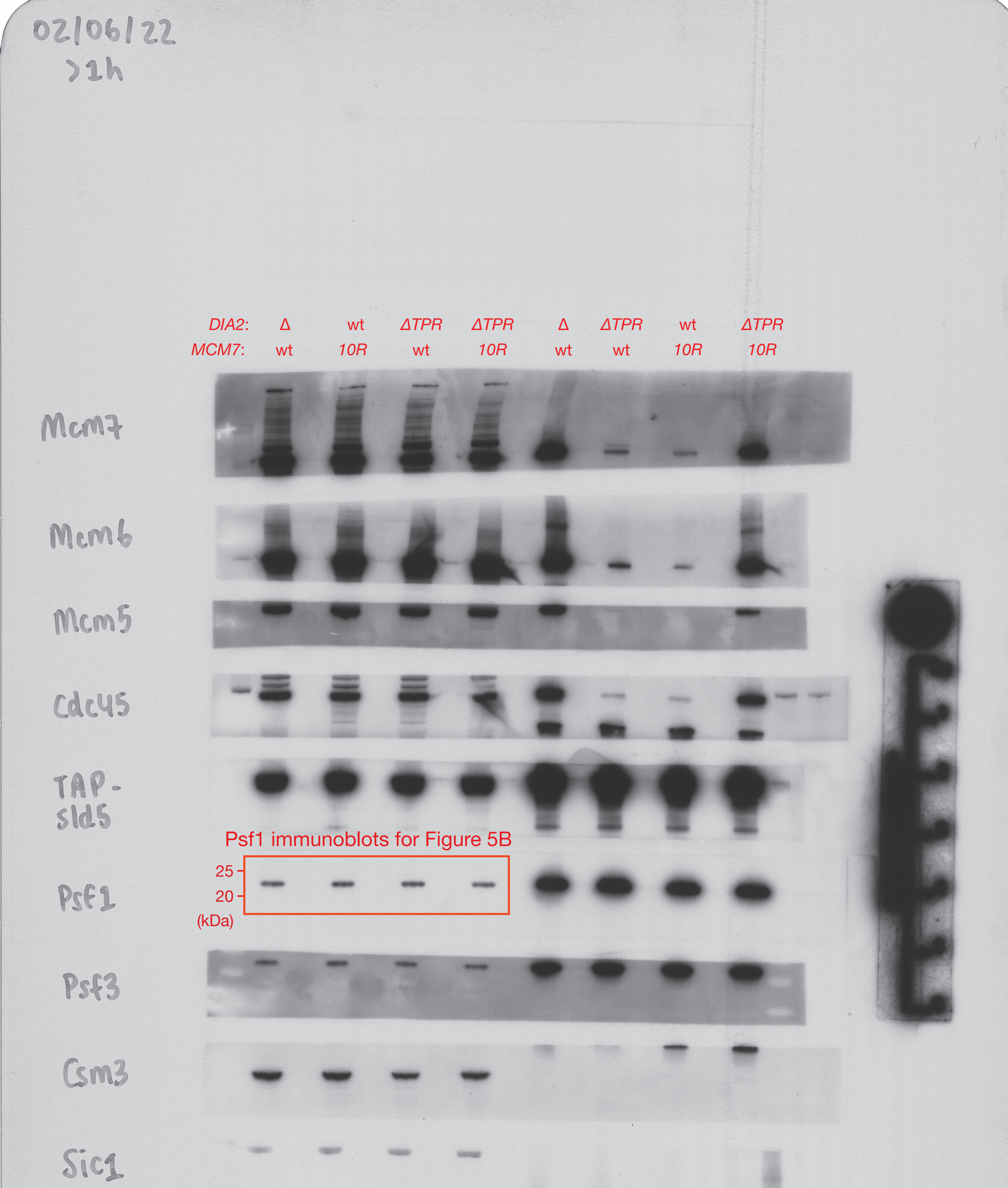
02/06/22
>1h
DIA2: Δ wt ΔTPR ΔTPR Δ ΔTPR wt ΔTPR
MCM7: wt 10R wt 10R wt wt 10R 10R
Mcm7
Mcm6
Mcm5
Cdc45
TAP-Sld5
Psf1 immunoblots for Figure 5B
25
20
(kDa)
Psf1
Psf3
Csm3
Sic1
