# Supplementary material for: CMG helicase disassembly is essential and driven by two pathways in budding yeast
Source: EMBO J. 2024 Jul 22;43(18):2. doi: 10.1038/s44318-024-00161-x (PMC11405719; doi:10.1038/s44318-024-00161-x)
Supplement: Supplementary file 12 — Source data Fig. 6 [file 44318_2024_161_MOESM12_ESM.zip › Source Data_Figure 6/6G/Figure 6G_Blot_Psf3.pdf]

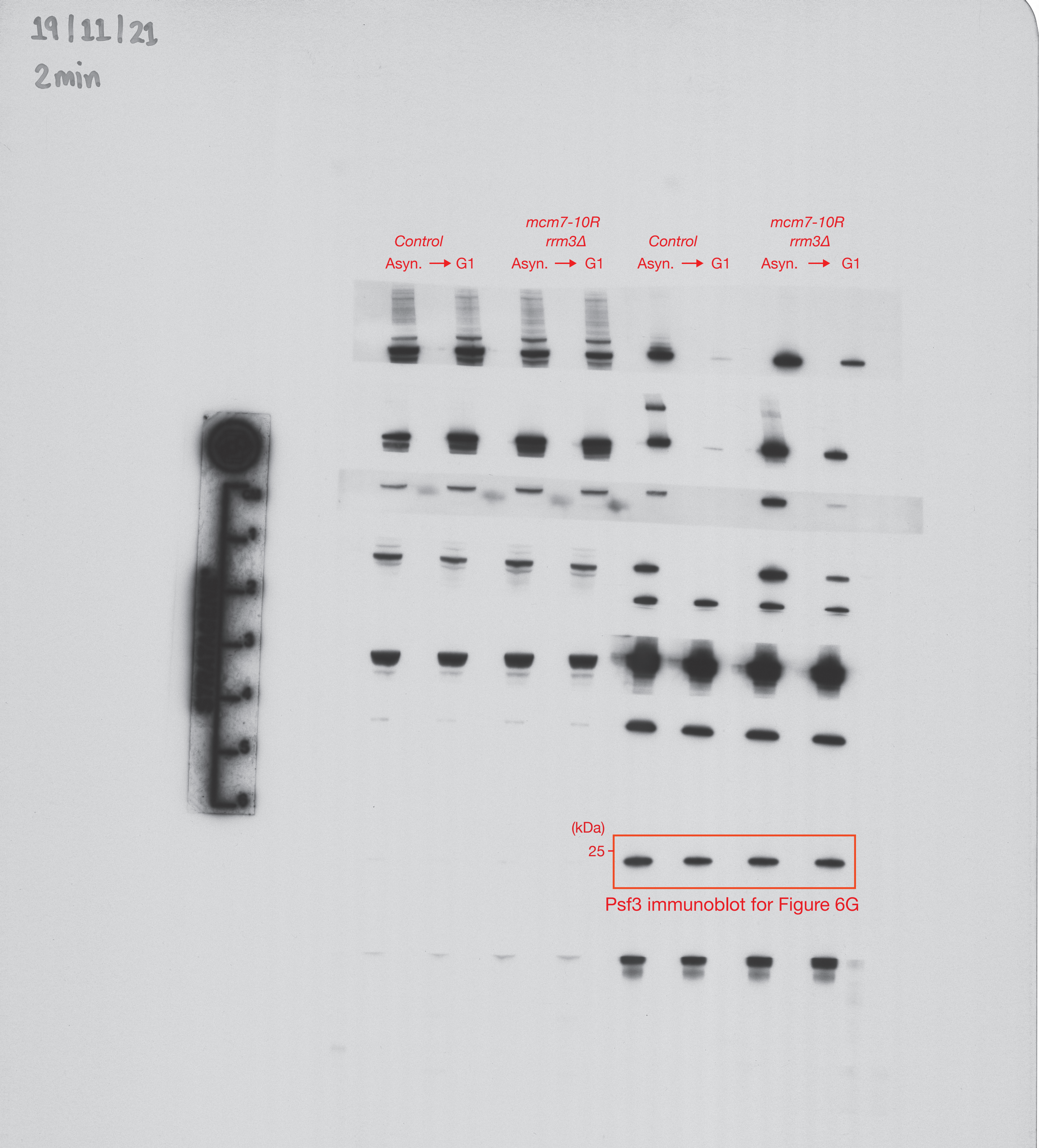
19/11/21
2min
Control
Asyn. → G1
mcm7-10R
rrm3Δ
Asyn. → G1
Control
Asyn. → G1
mcm7-10R
rrm3Δ
Asyn. → G1
(kDa)
25
Psf3 immunoblot for Figure 6G
