# Supplementary material for: CMG helicase disassembly is essential and driven by two pathways in budding yeast
Source: EMBO J. 2024 Jul 22;43(18):2. doi: 10.1038/s44318-024-00161-x (PMC11405719; doi:10.1038/s44318-024-00161-x)
Supplement: Supplementary file 12 — Source data Fig. 6 [file 44318_2024_161_MOESM12_ESM.zip › Source Data_Figure 6/6E/Figure 6E_Blots_Mcm7-Mcm6-Sld5.pdf]

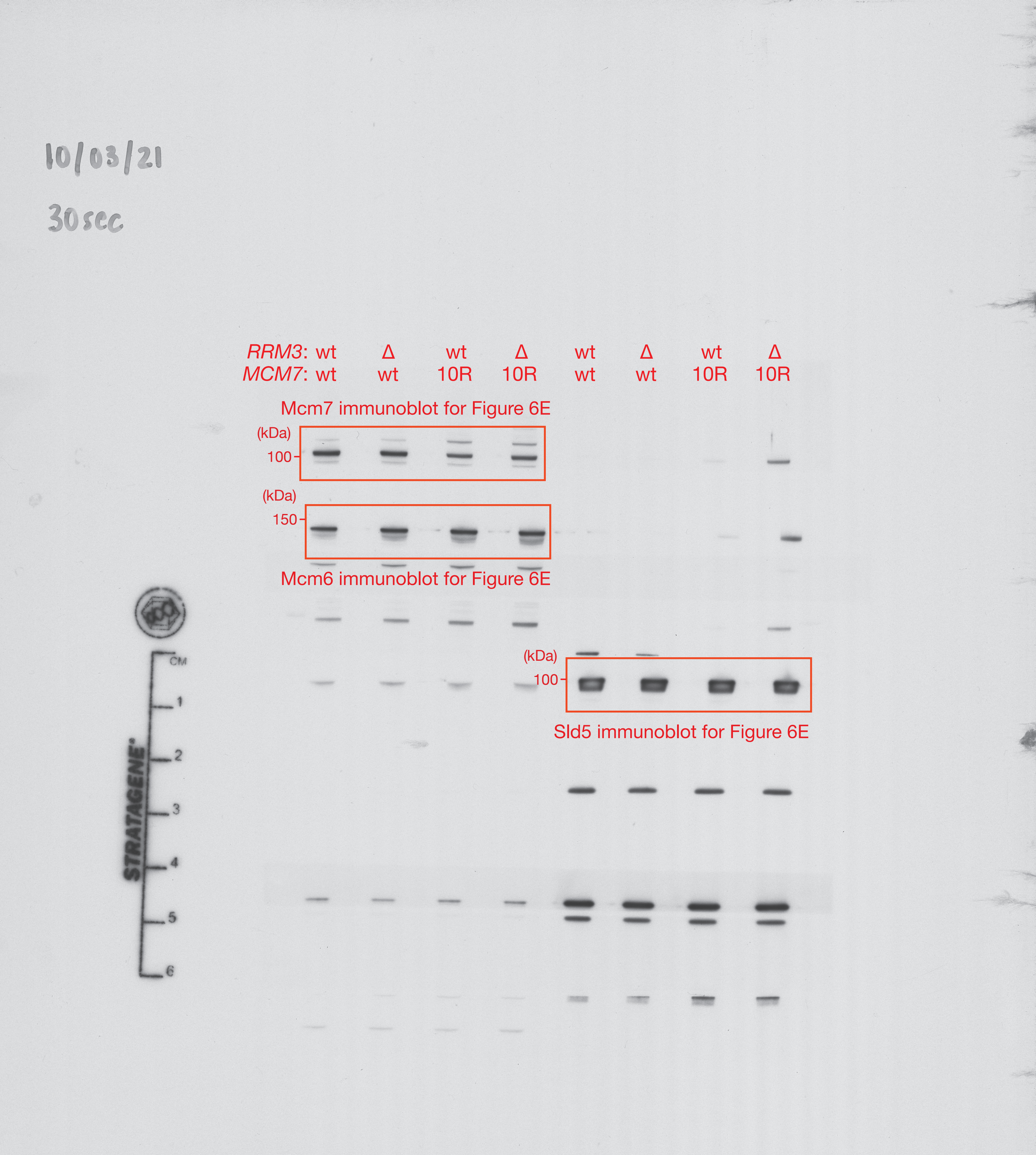
10/03/21
30sec
RRM3: wt Δ wt Δ wt Δ wt Δ
MCM7: wt wt 10R 10R wt wt 10R 10R
Mcm7 immunoblot for Figure 6E
(kDa)
100
(kDa)
150
Mcm6 immunoblot for Figure 6E
(kDa)
100
Sld5 immunoblot for Figure 6E
CM
1
2
3
4
5
6
STRATAGENE
