# Supplementary material for: CMG helicase disassembly is essential and driven by two pathways in budding yeast
Source: EMBO J. 2024 Jul 22;43(18):2. doi: 10.1038/s44318-024-00161-x (PMC11405719; doi:10.1038/s44318-024-00161-x)
Supplement: Supplementary file 13 — Source data Fig. 7 [file 44318_2024_161_MOESM13_ESM.zip › Source Data_Figure 7/7C/Figure 7C_Blots_Mcm7-Psf3.pdf]

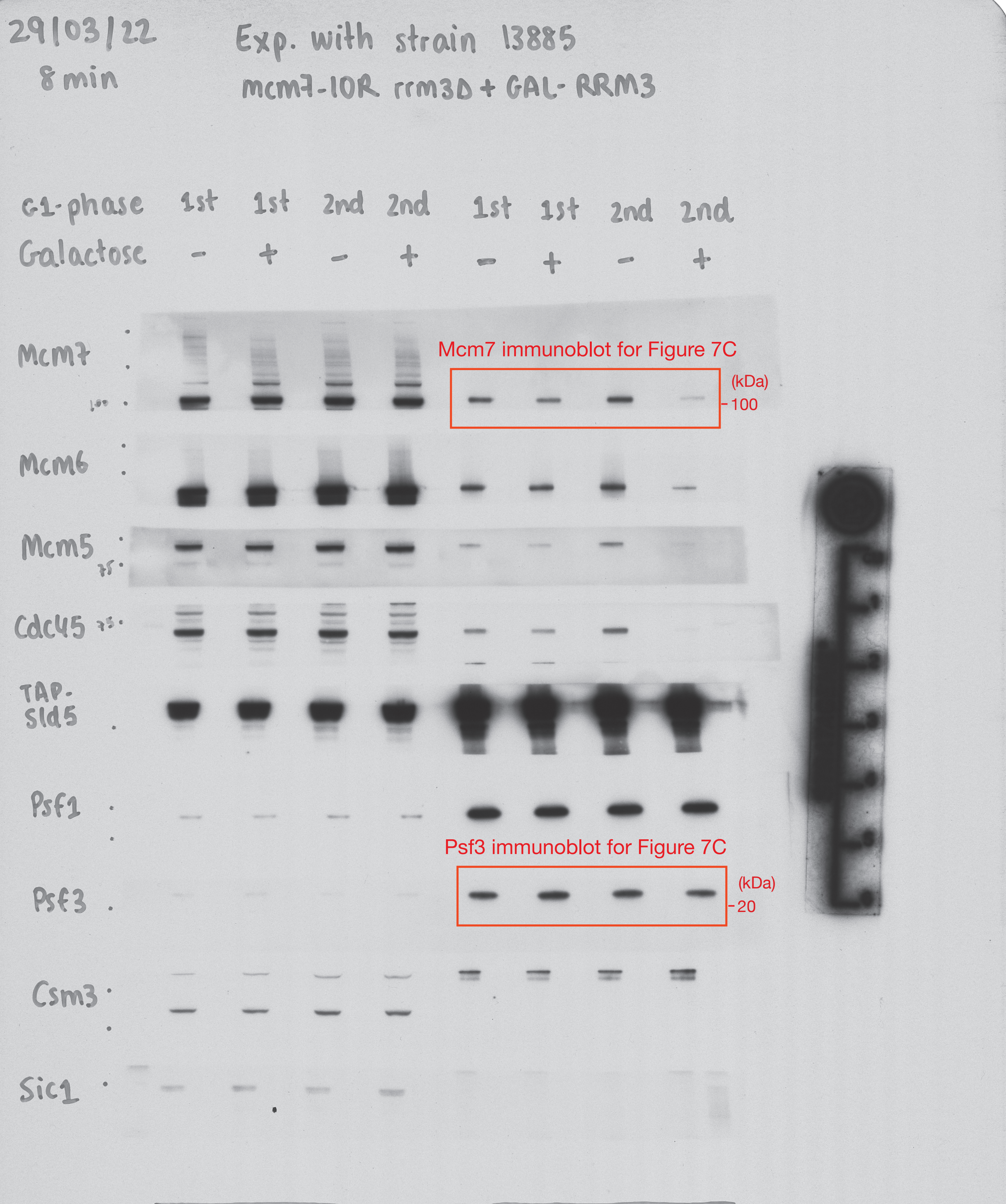
29/03/22
8 min
Exp. with strain 13885
mcm7-10R rrm3Δ + GAL-RRM3
G1-phase 1st 1st 2nd 2nd 1st 1st 2nd 2nd
Galactose - + - + - + - +
Mcm7
Mcm7 immunoblot for Figure 7C
(kDa)
100
Mcm6
Mcm5
Cdc45
TAP-Sld5
Psf1
Psf3 immunoblot for Figure 7C
Psf3
(kDa)
20
Csm3
Sic1
