# Supplementary material for: CMG helicase disassembly is essential and driven by two pathways in budding yeast
Source: EMBO J. 2024 Jul 22;43(18):2. doi: 10.1038/s44318-024-00161-x (PMC11405719; doi:10.1038/s44318-024-00161-x)
Supplement: Supplementary file 13 — Source data Fig. 7 [file 44318_2024_161_MOESM13_ESM.zip › Source Data_Figure 7/7C/Figure 7C_Blots_Mcm7-Psf1.pdf]

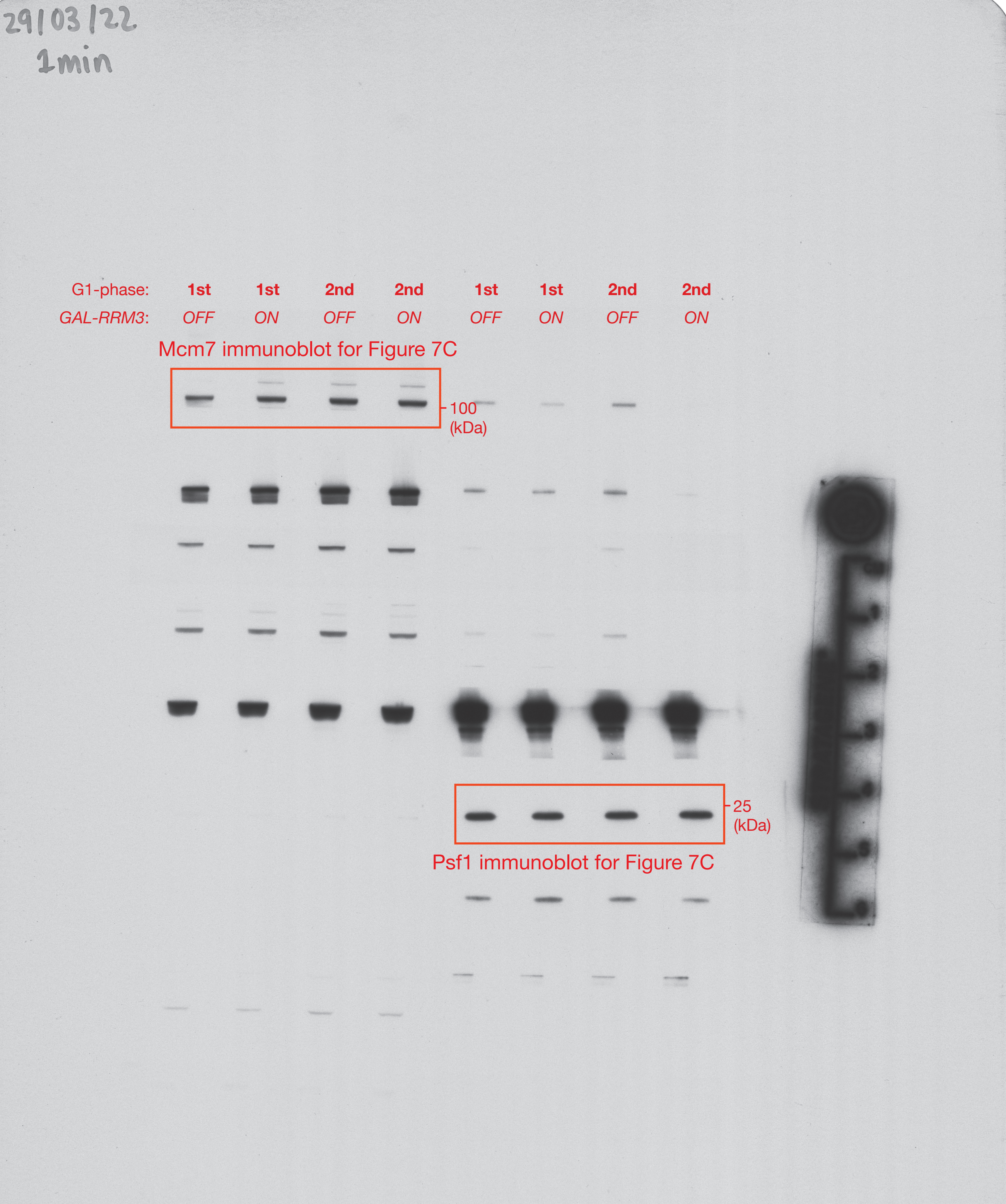
29/03/22
1min
G1-phase:
1st
1st
2nd
2nd
1st
1st
2nd
2nd
GAL-RRM3:
OFF
ON
OFF
ON
OFF
ON
OFF
ON
Mcm7 immunoblot for Figure 7C
100
(kDa)
25
(kDa)
Psf1 immunoblot for Figure 7C
