# Supplementary material for: CMG helicase disassembly is essential and driven by two pathways in budding yeast
Source: EMBO J. 2024 Jul 22;43(18):2. doi: 10.1038/s44318-024-00161-x (PMC11405719; doi:10.1038/s44318-024-00161-x)
Supplement: Supplementary file 13 — Source data Fig. 7 [file 44318_2024_161_MOESM13_ESM.zip › Source Data_Figure 7/7C/Figure 7C_Blots_Mcm6-Cdc45.pdf]

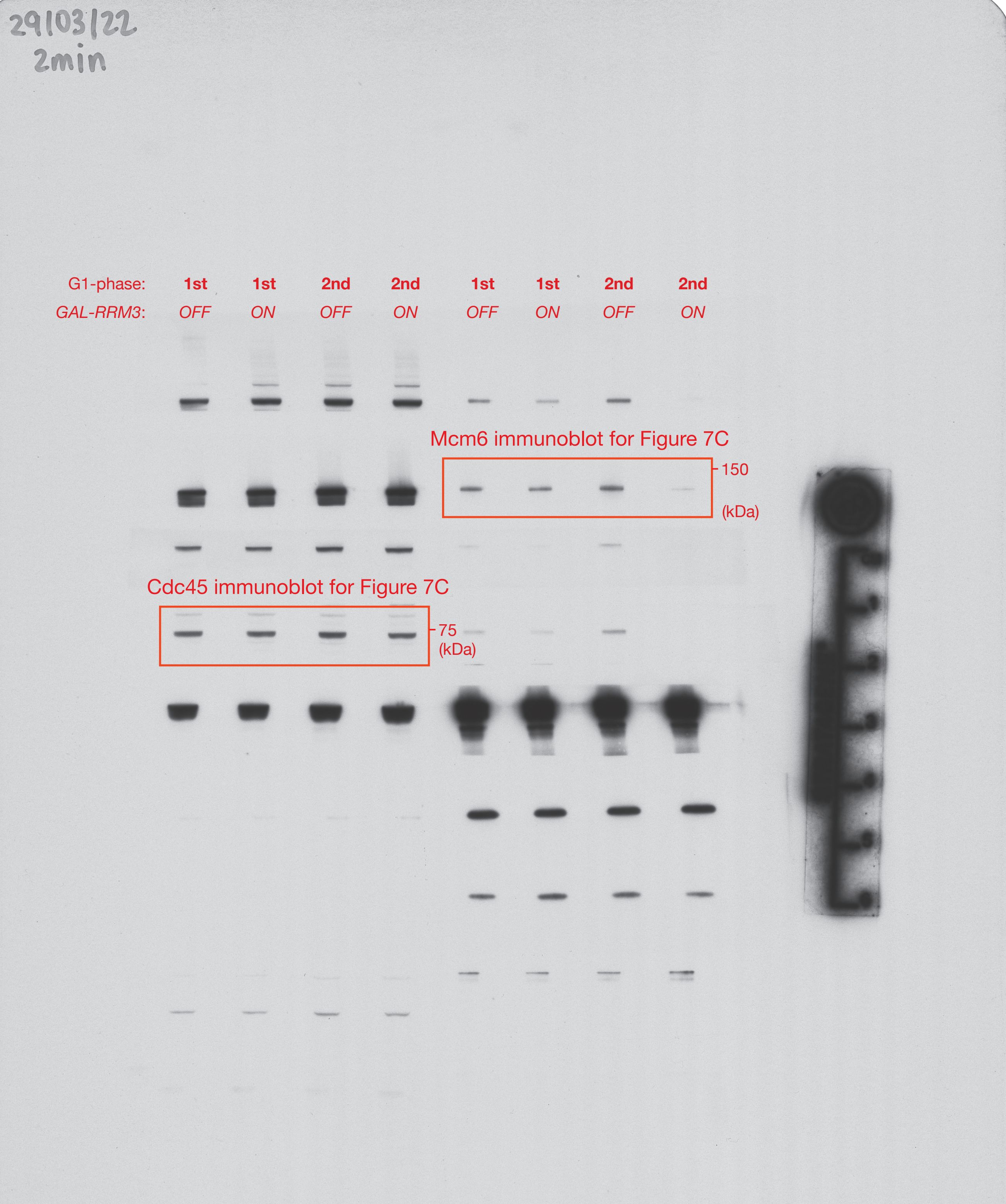
29/03/22
2min
G1-phase: 1st 1st 2nd 2nd 1st 1st 2nd 2nd
GAL-RRM3: OFF ON OFF ON OFF ON OFF ON
Mcm6 immunoblot for Figure 7C
150
(kDa)
Cdc45 immunoblot for Figure 7C
75
(kDa)
