# Supplementary material for: CMG helicase disassembly is essential and driven by two pathways in budding yeast
Source: EMBO J. 2024 Jul 22;43(18):2. doi: 10.1038/s44318-024-00161-x (PMC11405719; doi:10.1038/s44318-024-00161-x)
Supplement: Supplementary file 14 — Source data Fig. 8 [file 44318_2024_161_MOESM14_ESM.zip › Source Data_Figure 8/8C-D/Figure 8C-D_Blot_Mcm6.pdf]

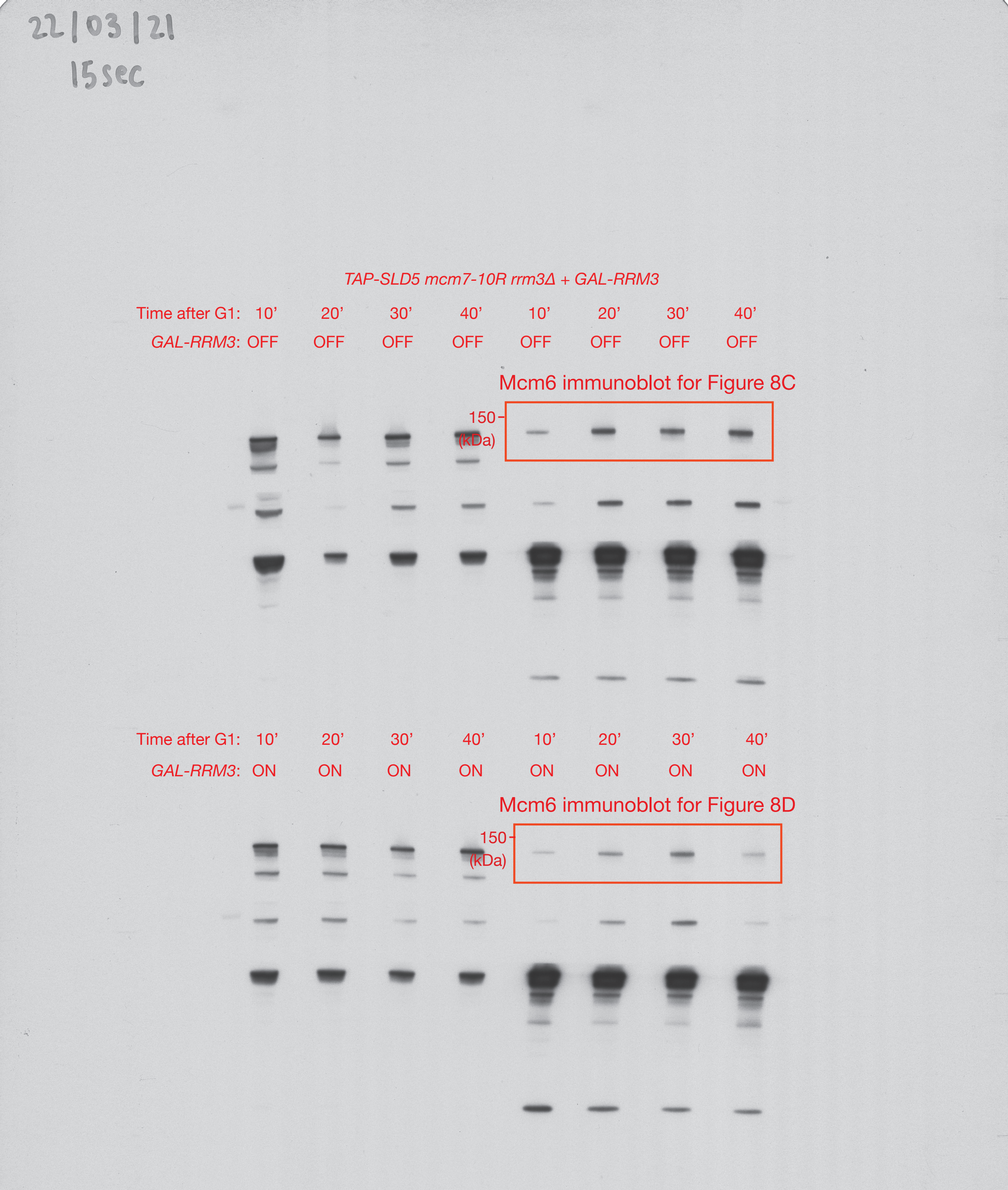
22/03/21
15sec
TAP-SLD5 mcm7-10R rrm3Δ + GAL-RRM3
Time after G1: 10' 20' 30' 40' 10' 20' 30' 40'
GAL-RRM3: OFF OFF OFF OFF OFF OFF OFF OFF
Mcm6 immunoblot for Figure 8C
150
(kDa)
Time after G1: 10' 20' 30' 40' 10' 20' 30' 40'
GAL-RRM3: ON ON ON ON ON ON ON ON
Mcm6 immunoblot for Figure 8D
150
(kDa)
