# Supplementary material for: CMG helicase disassembly is essential and driven by two pathways in budding yeast
Source: EMBO J. 2024 Jul 22;43(18):2. doi: 10.1038/s44318-024-00161-x (PMC11405719; doi:10.1038/s44318-024-00161-x)
Supplement: Supplementary file 16 — Expanded View Figures [file 44318_2024_161_MOESM16_ESM.pdf]

Expanded View Figures

Figure EV1. Model for disassembly of CMG helicase in wild-type cells and *dia2*Δ.

(**A**) In wild-type budding yeast cells, ubiquitylation of CMG by SCF^{Dia2} is sterically impeded at replication forks, by the parental DNA strand that is excluded from the Mcm2–7 ring of the helicase. This inhibition is released during DNA replication termination, when a pair of forks converge and the two CMG helicases bypass each other, thereby breaking the association between CMG and the excluded parental DNA strand. (**B**) In *dia2Δ* cells, CMG cannot be ubiquitylated during DNA replication termination and persists on chromatin until the next cell cycle. However, once *dia2Δ* enter S-phase, the old CMG complexes are disassembled by a previously unknown pathway, likely coupled to the encounter between new replication forks and old CMG.

 © The Author(s)

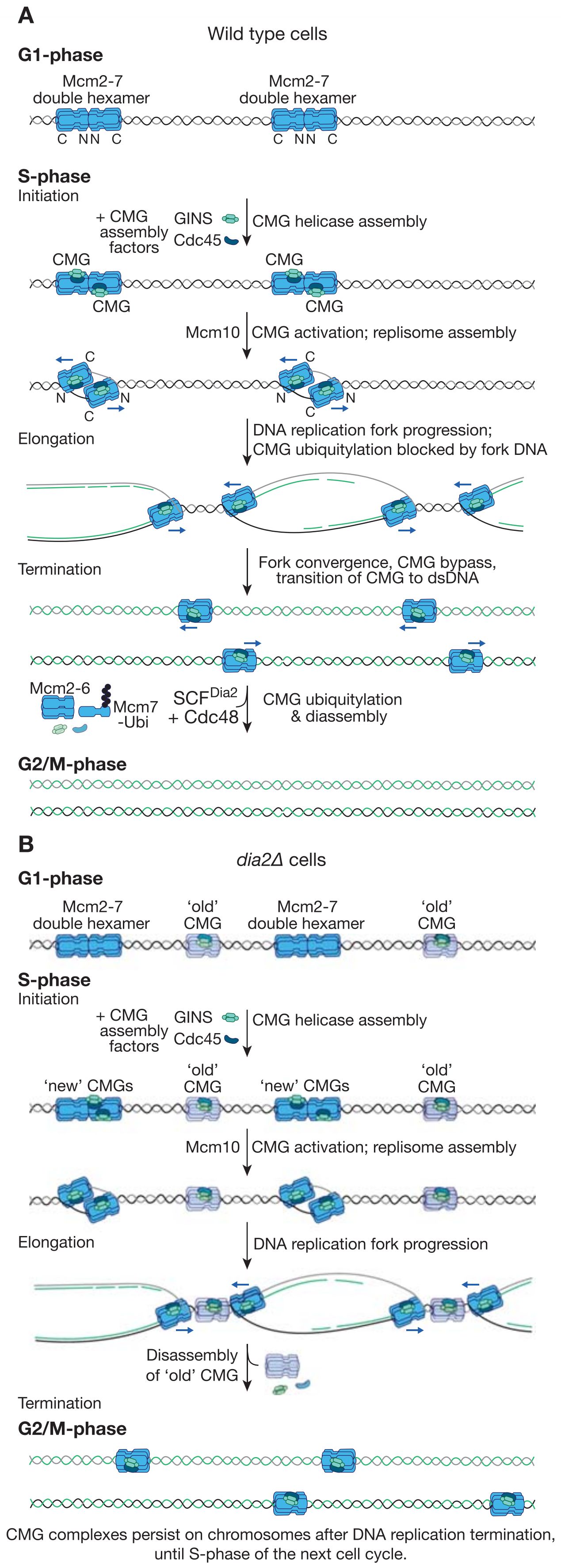
A
Wild type cells
G1-phase
Mcm2-7
double hexamer
C NN C
Mcm2-7
double hexamer
C NN C
S-phase
Initiation
+ CMG
assembly
factors
GINS
Cdc45
CMG helicase assembly
CMG
CMG
CMG
CMG
Mcm10
CMG activation; replisome assembly
Elongation
DNA replication fork progression;
CMG ubiquitylation blocked by fork DNA
Termination
Fork convergence, CMG bypass,
transition of CMG to dsDNA
Mcm2-6
Mcm7
-Ubi
SCFDia2
+ Cdc48
CMG ubiquitylation
& diassembly
G2/M-phase
B
dia2Δ cells
G1-phase
Mcm2-7
double hexamer
'old'
CMG
Mcm2-7
double hexamer
'old'
CMG
S-phase
Initiation
+ CMG
assembly
factors
GINS
Cdc45
CMG helicase assembly
'new' CMGs
'old'
CMG
'new' CMGs
'old'
CMG
Mcm10
CMG activation; replisome assembly
Elongation
DNA replication fork progression
Disassembly
of 'old' CMG
Termination
G2/M-phase
CMG complexes persist on chromosomes after DNA replication termination,
until S-phase of the next cell cycle.

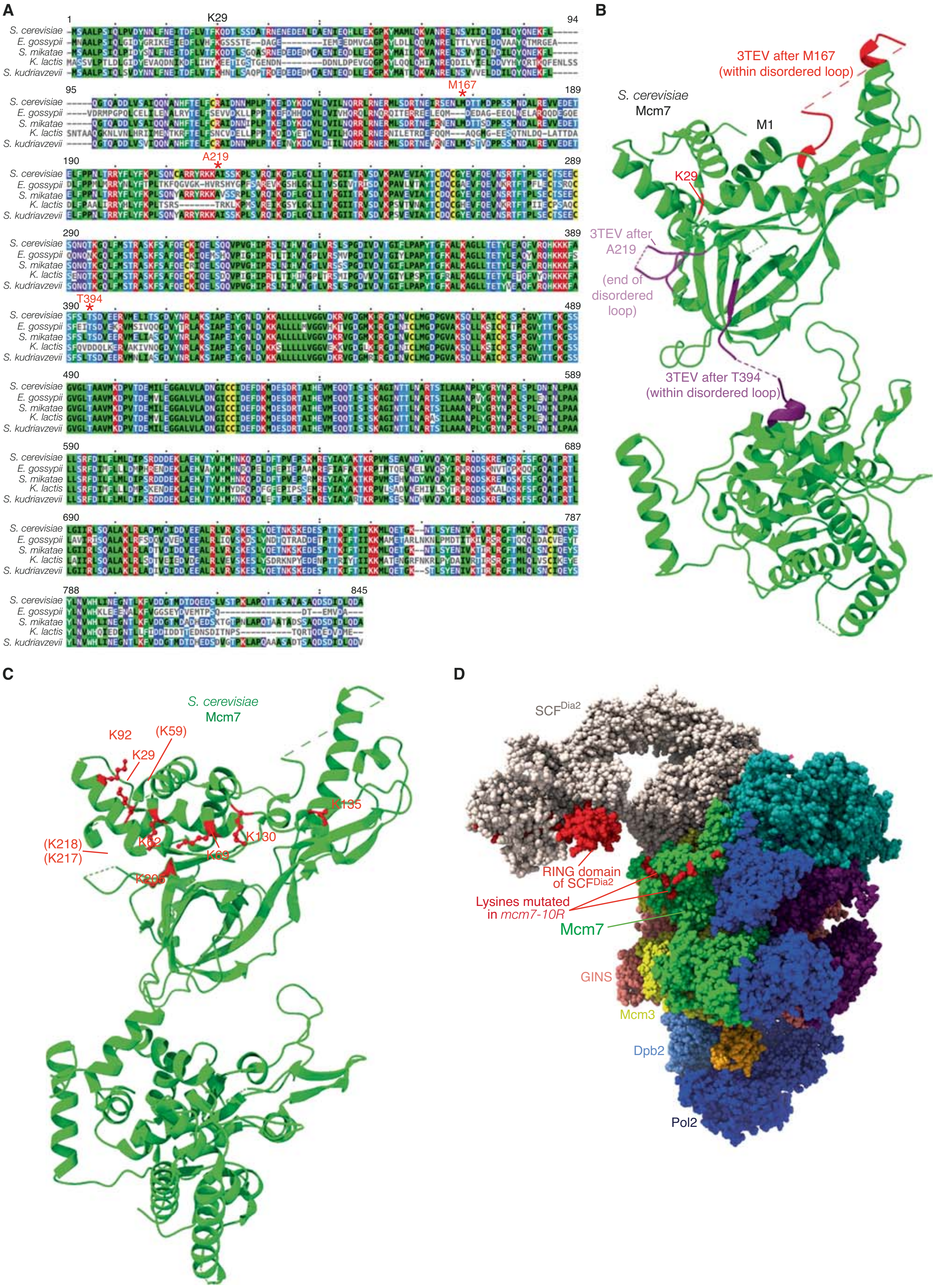
A
S. cerevisiae
E. gossypii
S. mikatae
K. lactis
S. kudriavzevii
1
K29
94
95
M167
189
190
A219
289
290
389
390
T394
489
490
589
590
689
690
787
788
845
B
S. cerevisiae
Mcm7
M1
K29
3TEV after M167
(within disordered loop)
3TEV after
A219
(end of
disordered
loop)
3TEV after T394
(within disordered loop)
C
S. cerevisiae
Mcm7
K92
(K59)
K29
K135
(K218)
(K217)
K62
K69
K130
K205
D
SCF^Dia2
RING domain
of SCF^Dia2
Lysines mutated
in mcm7-10R
Mcm7
GINS
Mcm3
Dpb2
Pol2

© The Author(s)

Figure EV2. Location of TEV cleavage sites and SCF^{Dia2} ubiquitylation sites in Mcm7.

(**A**) Mcm7 from multiple yeast species (*S. cerevisiae* = *Saccharomyces cerevisiae*; *E. gossypii* = *Eremothecium gossypii*; *S. mikatae* = *Saccharomyces mikatae*; *K. lactis* = *Kluyveromyces lactis*; *S. kudriavzevii* = *Saccharomyces kudriavzevii*) were aligned using Clustal Omega software. Mcm7-K29 is marked in black, whereas the three sites used for insertion of TEV cleavage sites (M167, A219 and T394) are shown in red with an asterisk. (**B**) The structure of *S. cerevisiae* Mcm7 (from PDB file 7PMK), illustrating the location of K29 and the three sites within disordered loops that were used to insert three consecutive TEV cleavage sites (after M167, A219 or T394 of Mcm7). Also see Movie EV1. (**C**) Structure of yeast Mcm7 (from PDB file 7PMK) and location in red of the 10 lysines mutated in Mcm7-10R. Note that K217 and K218 are in a disordered loop not visible in the cryoEM structure of the yeast replisome. (**D**) The structure of SCF^{Dia2} was predicted with AlphaFold2-Multimer and then docked onto the cryoEM structure of the yeast replisome (adapted from PDB file 7PMK) with UCSF Chimera software. The RING domain of Dia2, and the 10 lysines mutated in Mcm7-10R, are shown in red. Also see Movie EV2.

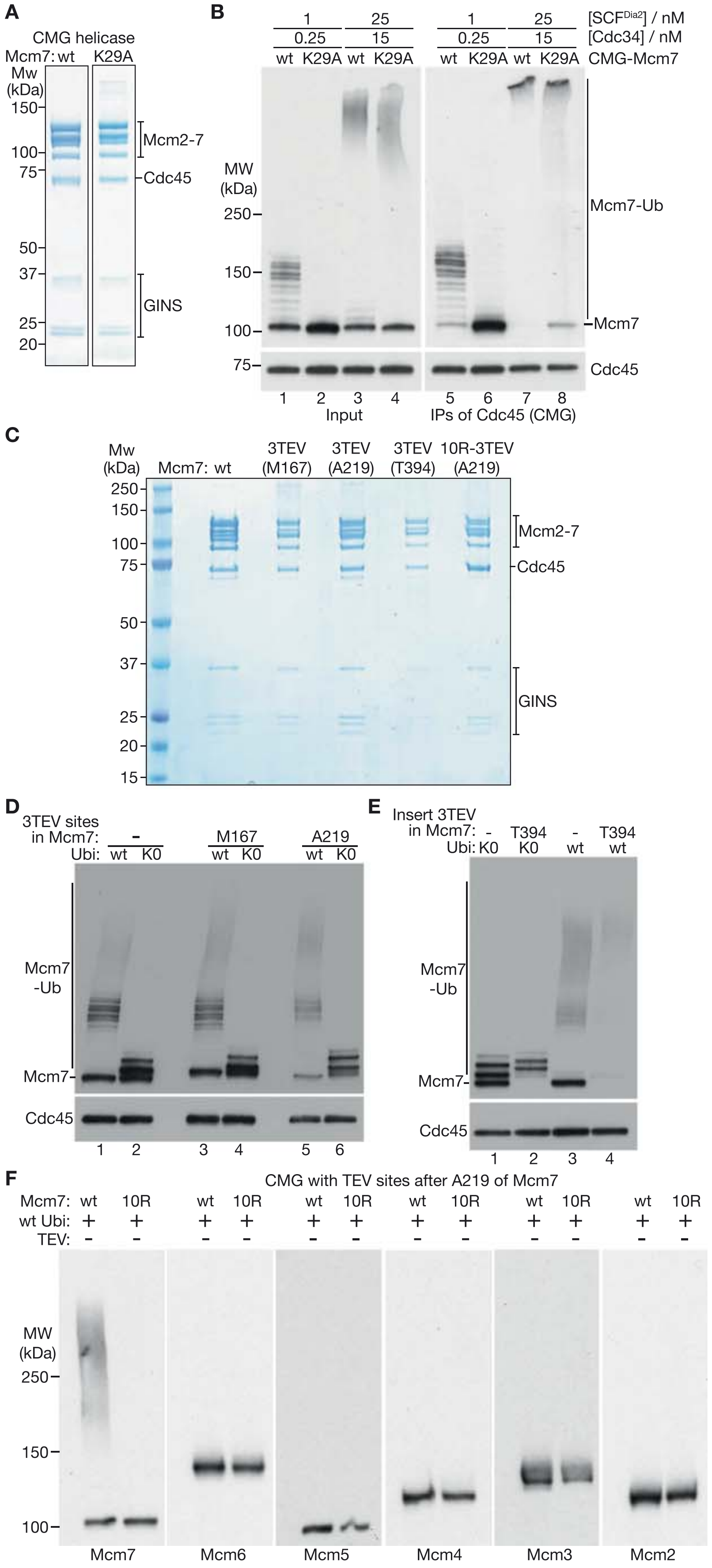
A
CMG helicase
Mcm7: wt K29A
Mw (kDa)
150
100
75
50
37
25
20
Mcm2-7
Cdc45
GINS
B
[SCF^{Dia2}] / nM
[Cdc34] / nM
CMG-Mcm7
1 25 1 25
0.25 15 0.25 15
wt K29A wt K29A wt K29A wt K29A
MW (kDa)
250
150
100
75
Mcm7-Ub
Mcm7
Cdc45
1 2 3 4 5 6 7 8
Input
IPs of Cdc45 (CMG)
C
Mw (kDa)
Mcm7: wt
3TEV (M167)
3TEV (A219)
3TEV (T394)
10R-3TEV (A219)
250
150
100
75
50
37
25
20
15
Mcm2-7
Cdc45
GINS
D
3TEV sites in Mcm7:
-
M167
A219
Ubi: wt K0 wt K0 wt K0
Mcm7 -Ub
Mcm7
Cdc45
1 2 3 4 5 6
E
Insert 3TEV in Mcm7: - T394 - T394
Ubi: K0 K0 wt wt
Mcm7 -Ub
Mcm7
Cdc45
1 2 3 4
F
CMG with TEV sites after A219 of Mcm7
Mcm7: wt 10R wt 10R wt 10R wt 10R wt 10R wt 10R
wt Ubi: + + + + + + + + + + + +
TEV: - - - - - - - - - - - -
MW (kDa)
250
150
100
Mcm7
Mcm6
Mcm5
Mcm4
Mcm3
Mcm2

 © The Author(s)

Figure EV3. Generation and characterisation of recombinant CMG complexes with mutated alleles of Mcm7.

(**A**) Purified recombinant budding yeast CMG helicase, with wild-type Mcm7 or Mcm7-K29A, were resolved by SDS-PAGE, before staining of the gel with Coomassie blue. (**B**) CMG containing wild-type (wt) Mcm7 or Mcm7-K29A was ubiquitylated in the presence of the indicated concentrations of SCF^{Dia2} and Cdc34. The reactions were analysed by immunoblotting. (**C**) Coomassie-stained gel with purified recombinant CMG containing wild-type Mcm7 or the indicated variants. (**D**, **E**) Recombinant CMG with the indicated versions of Mcm7 were ubiquitylated in vitro by SCFDia2 and Cdc34, using either wild-type ubiquitin (wt Ubi) or lysine-free ubiquitin (K0 Ubi). Reactions were then analysed by immunoblotting. Note that the ubiquitylated forms in lanes 3–6 of (**D**), and lanes 2 and 4 of (**E**), are shifted by comparison with the control, due to the insertion into Mcm7 of peptide sequences containing TEV cleavage sites (the sites were not cleaved in these reactions). (**F**) Analogous reactions to those in D–E were performed with wild-type ubiquitin. The Mcm2–7 subunits of CMG were monitored by immunoblotting. Source data are available online for this figure.

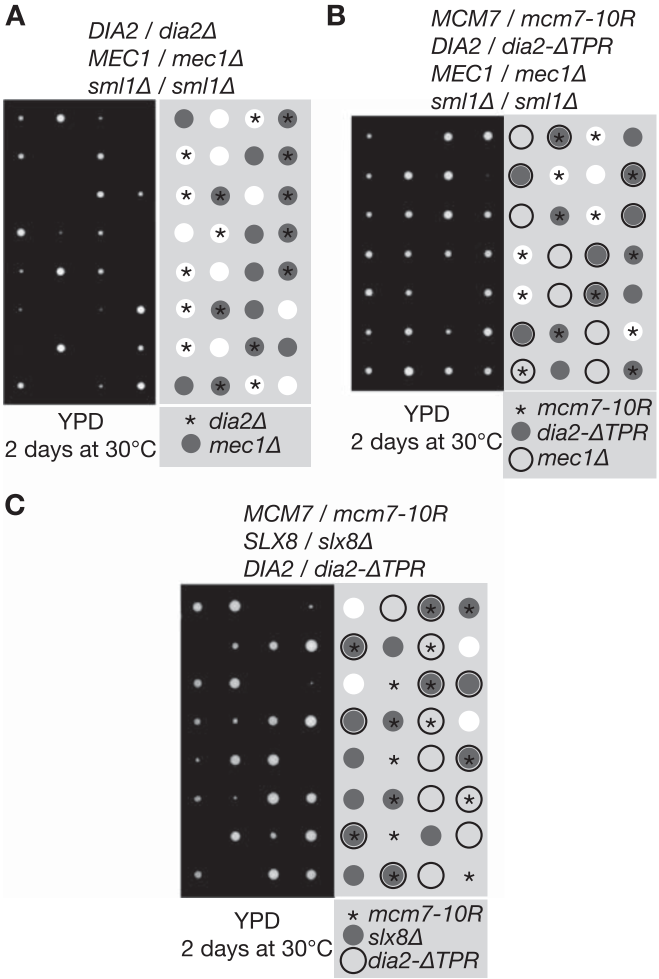

Figure EV4. The S-phase checkpoint and Slx8 become essential when the ubiquitylation of CMG-Mcm7 is blocked.

(A–C) Tetrad analysis of diploid budding yeast cells of the indicated genotypes (YCPR486, YCPR500, YCPR222). YPD = medium comprising Yeast Extract, Peptone, Dextrose. Source data are available online for this figure.

 © The Author(s)

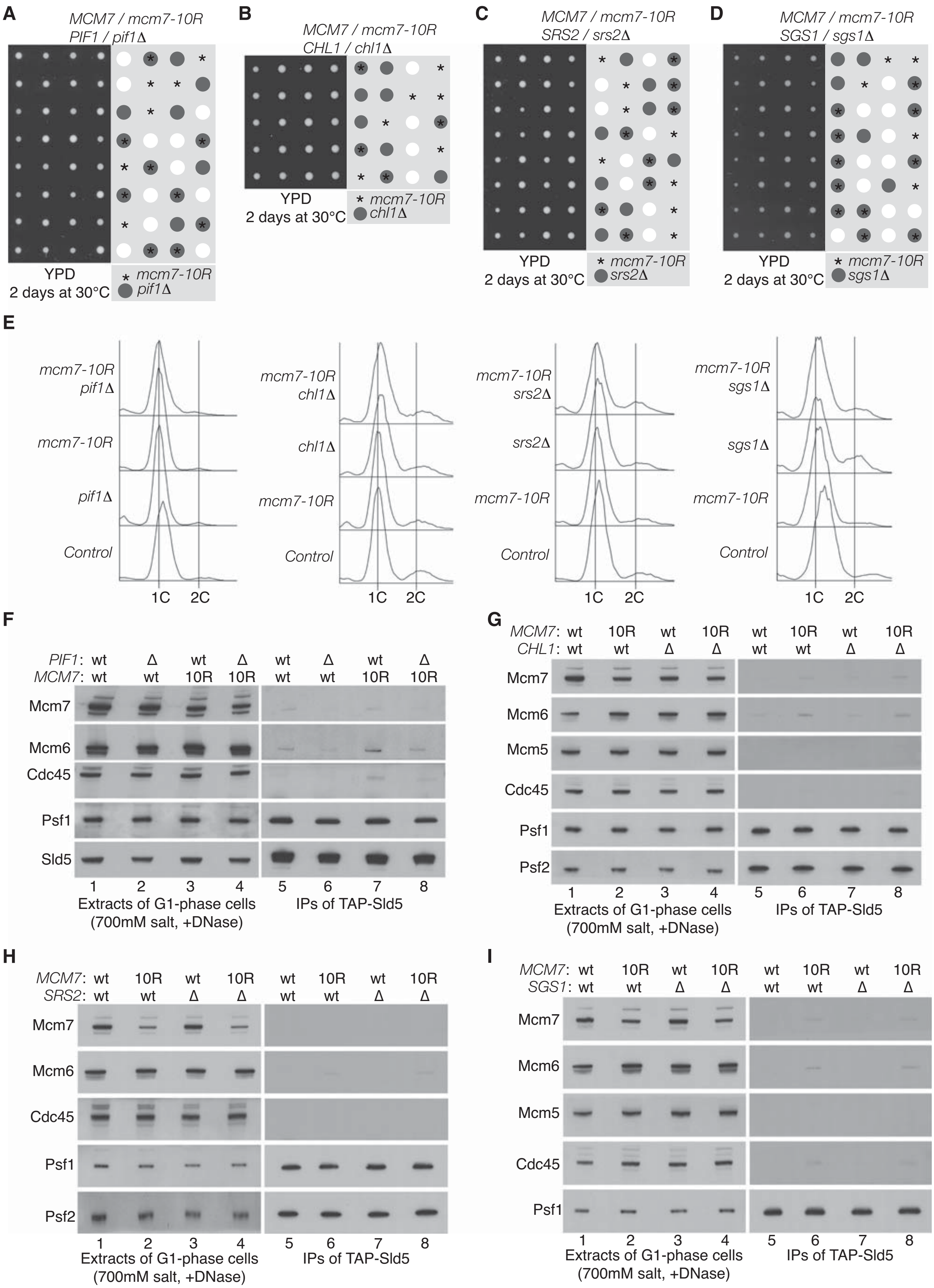
A
MCM7 / mcm7-10R
PIF1 / pif1Δ
YPD
2 days at 30°C
* mcm7-10R
pif1Δ
B
MCM7 / mcm7-10R
CHL1 / chl1Δ
YPD
2 days at 30°C
* mcm7-10R
chl1Δ
C
MCM7 / mcm7-10R
SRS2 / srs2Δ
YPD
2 days at 30°C
* mcm7-10R
srs2Δ
D
MCM7 / mcm7-10R
SGS1 / sgs1Δ
YPD
2 days at 30°C
* mcm7-10R
sgs1Δ
E
mcm7-10R pif1Δ
mcm7-10R
pif1Δ
Control
mcm7-10R chl1Δ
chl1Δ
mcm7-10R
Control
mcm7-10R srs2Δ
srs2Δ
mcm7-10R
Control
mcm7-10R sgs1Δ
sgs1Δ
mcm7-10R
Control
1C 2C
F
PIF1: wt Δ wt Δ wt Δ wt Δ
MCM7: wt wt 10R 10R wt wt 10R 10R
Mcm7
Mcm6
Cdc45
Psf1
Sld5
1 2 3 4 5 6 7 8
Extracts of G1-phase cells (700mM salt, +DNase)
IPs of TAP-Sld5
G
MCM7: wt 10R wt 10R wt 10R wt 10R
CHL1: wt wt Δ Δ wt wt Δ Δ
Mcm7
Mcm6
Mcm5
Cdc45
Psf1
Psf2
1 2 3 4 5 6 7 8
Extracts of G1-phase cells (700mM salt, +DNase)
IPs of TAP-Sld5
H
MCM7: wt 10R wt 10R wt 10R wt 10R
SRS2: wt wt Δ Δ wt wt Δ Δ
Mcm7
Mcm6
Cdc45
Psf1
Psf2
1 2 3 4 5 6 7 8
Extracts of G1-phase cells (700mM salt, +DNase)
IPs of TAP-Sld5
I
MCM7: wt 10R wt 10R wt 10R wt 10R
SGS1: wt wt Δ Δ wt wt Δ Δ
Mcm7
Mcm6
Mcm5
Cdc45
Psf1
1 2 3 4 5 6 7 8
Extracts of G1-phase cells (700mM salt, +DNase)
IPs of TAP-Sld5

© The Author(s)

Figure EV5. Combination of *mcm7-10R* with *pif1*Δ, *chl1*Δ, *srs2*Δ or *sgs1*Δ does not cause synthetic lethality or accumulation of CMG during G1 phase.

(**A–D**) Tetrad analysis of diploid budding yeast cells of the indicated genotypes (YCPR75, YCPR435, YCPR434, YCPR171). YPD = Yeast Extract, Peptone, Dextrose. (**E**) The indicated strains (YCPR141, YCPR406, YCPR412, YCPR428) were arrested in G1 phase at 30 °C by addition of mating pheromone. DNA content was monitored by flow cytometry. (**F–I**) Cell extracts were prepared from the samples in (**E**) and used to isolate GINS and the CMG helicase by immunoprecipitation of TAP-Sld5. The indicated factors were monitored by immunoblotting. Source data are available online for this figure.

 © The Author(s)
