# Supplementary material for: CMG helicase disassembly is essential and driven by two pathways in budding yeast
Source: EMBO J. 2024 Jul 22;43(18):2. doi: 10.1038/s44318-024-00161-x (PMC11405719; doi:10.1038/s44318-024-00161-x)
Supplement: Supplementary file 15 — EV Figure Source Data [file 44318_2024_161_MOESM15_ESM.zip › Source_Data_Figure_EV3.zip!/Source Data_Figure EV3/EV3F/Figure EV3F_Blots_Mcm4-Mcm3-Mcm2.pdf]

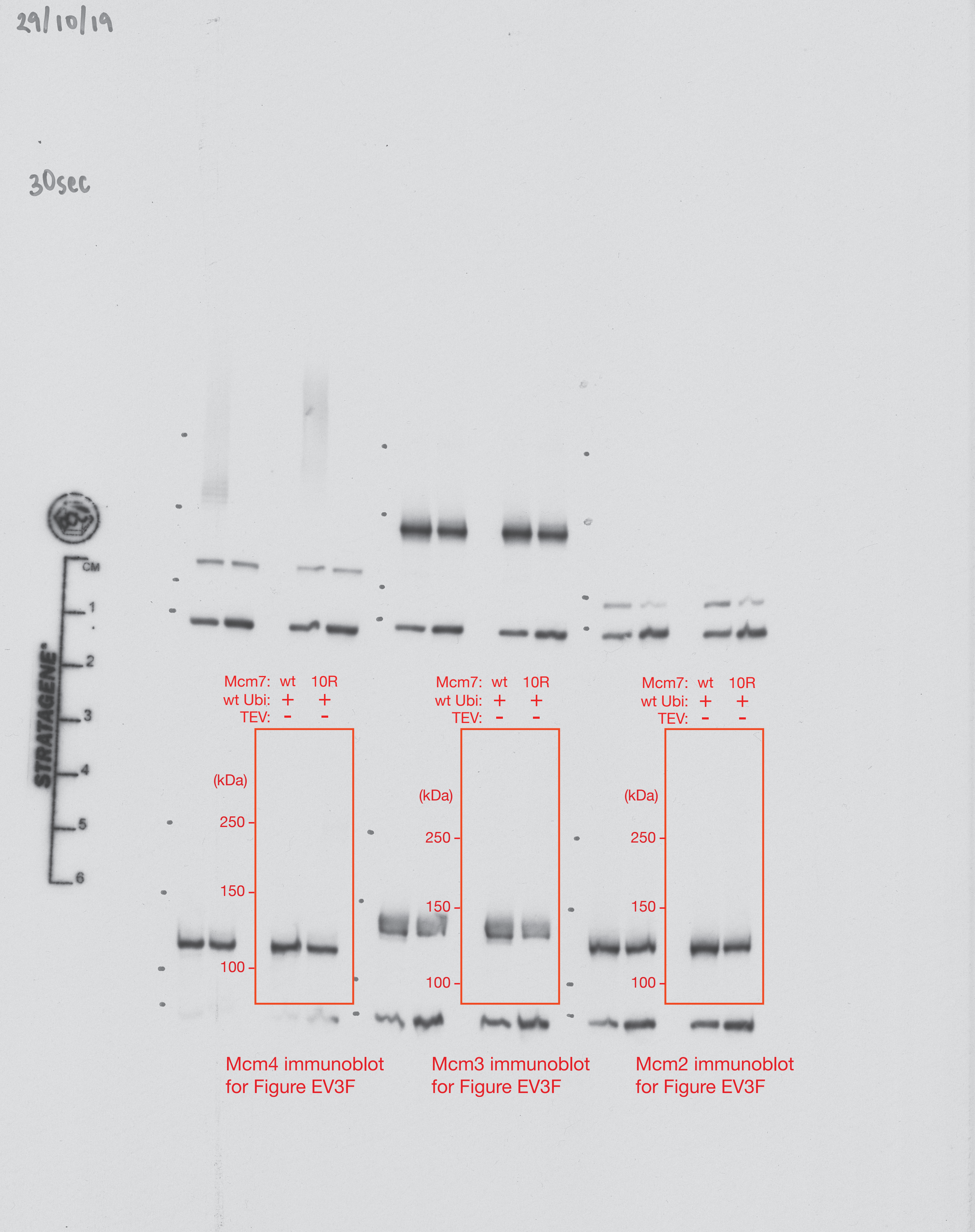
29/10/19
30sec
STRATAGENE
CM
1
2
3
4
5
6
Mcm7: wt 10R
wt Ubi: + +
TEV: - -
(kDa)
250
150
100
Mcm4 immunoblot for Figure EV3F
Mcm7: wt 10R
wt Ubi: + +
TEV: - -
(kDa)
250
150
100
Mcm3 immunoblot for Figure EV3F
Mcm7: wt 10R
wt Ubi: + +
TEV: - -
(kDa)
250
150
100
Mcm2 immunoblot for Figure EV3F
