# Supplementary material for: CMG helicase disassembly is essential and driven by two pathways in budding yeast
Source: EMBO J. 2024 Jul 22;43(18):2. doi: 10.1038/s44318-024-00161-x (PMC11405719; doi:10.1038/s44318-024-00161-x)
Supplement: Supplementary file 15 — EV Figure Source Data [file 44318_2024_161_MOESM15_ESM.zip › Source_Data_Figure_EV3.zip!/Source Data_Figure EV3/EV3F/Figure EV3F_Blots_Mcm7-Mcm5.pdf]

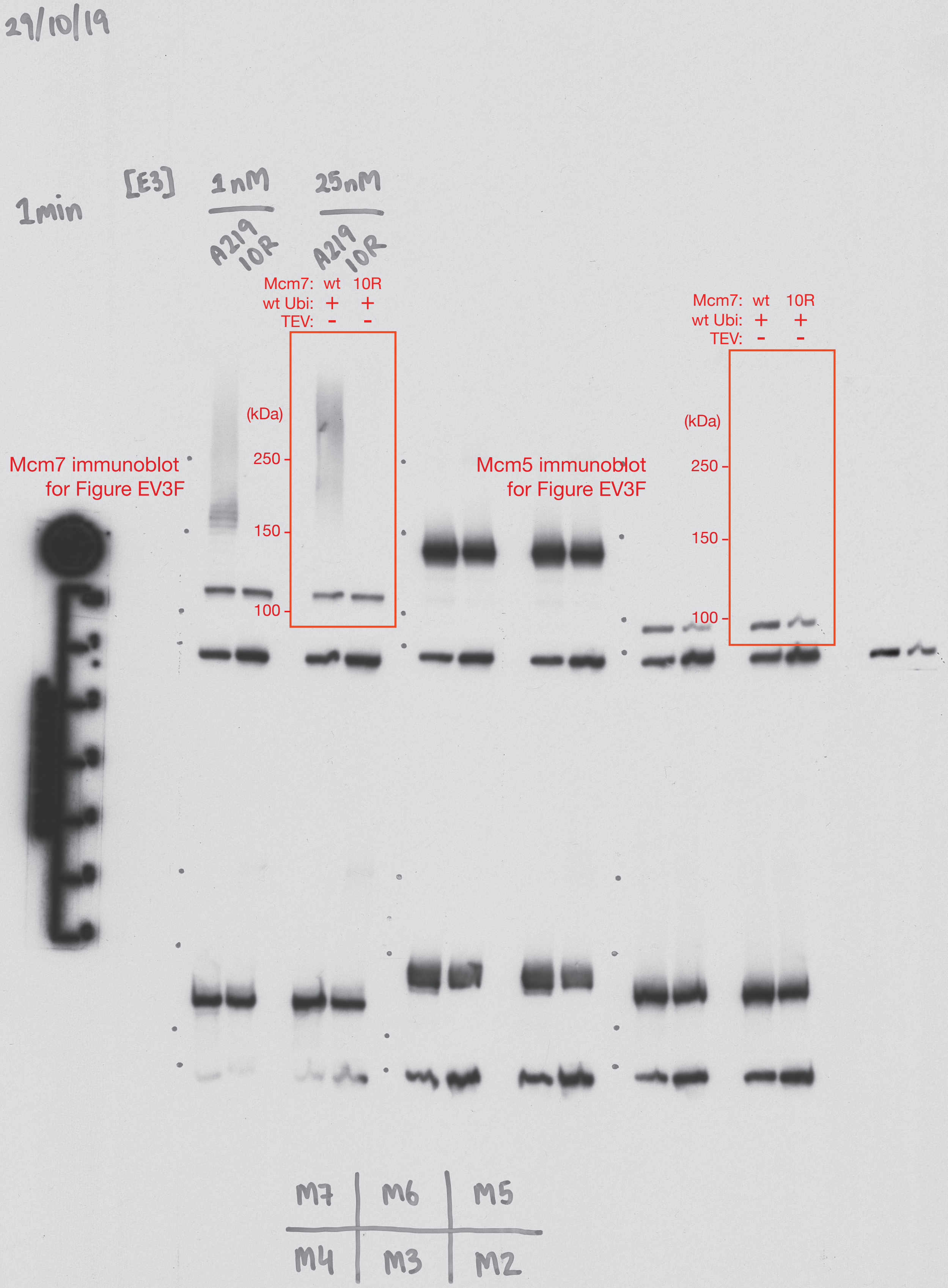
29/10/19
1min
[E3]
1nM
25nM
A219
10R
A219
10R
Mcm7: wt 10R
wt Ubi: + +
TEV: - -
(kDa)
250
150
100
Mcm7 immunoblot for Figure EV3F
Mcm5 immunoblot for Figure EV3F
Mcm7: wt 10R
wt Ubi: + +
TEV: - -
(kDa)
250
150
100
M7
M6
M5
M4
M3
M2
