# Supplementary material for: CMG helicase disassembly is essential and driven by two pathways in budding yeast
Source: EMBO J. 2024 Jul 22;43(18):2. doi: 10.1038/s44318-024-00161-x (PMC11405719; doi:10.1038/s44318-024-00161-x)
Supplement: Supplementary file 15 — EV Figure Source Data [file 44318_2024_161_MOESM15_ESM.zip › Source_Data_Figure_EV3.zip!/Source Data_Figure EV3/EV3A/Figure EV3A_Coomassie Gel_Mcm7-K29A.pdf]

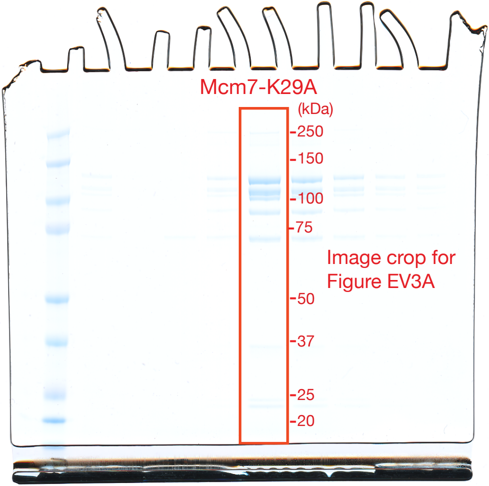

Mcm7-K29A
(kDa)
-250
-150
-100
-75
Image crop for
Figure EV3A
-50
-37
-25
-20
