# Supplementary material for: CMG helicase disassembly is essential and driven by two pathways in budding yeast
Source: EMBO J. 2024 Jul 22;43(18):2. doi: 10.1038/s44318-024-00161-x (PMC11405719; doi:10.1038/s44318-024-00161-x)
Supplement: Supplementary file 15 — EV Figure Source Data [file 44318_2024_161_MOESM15_ESM.zip › Source_Data_Figure_EV3.zip!/Source Data_Figure EV3/EV3D/Figure EV3D_Mcm7-Cdc45.pdf]

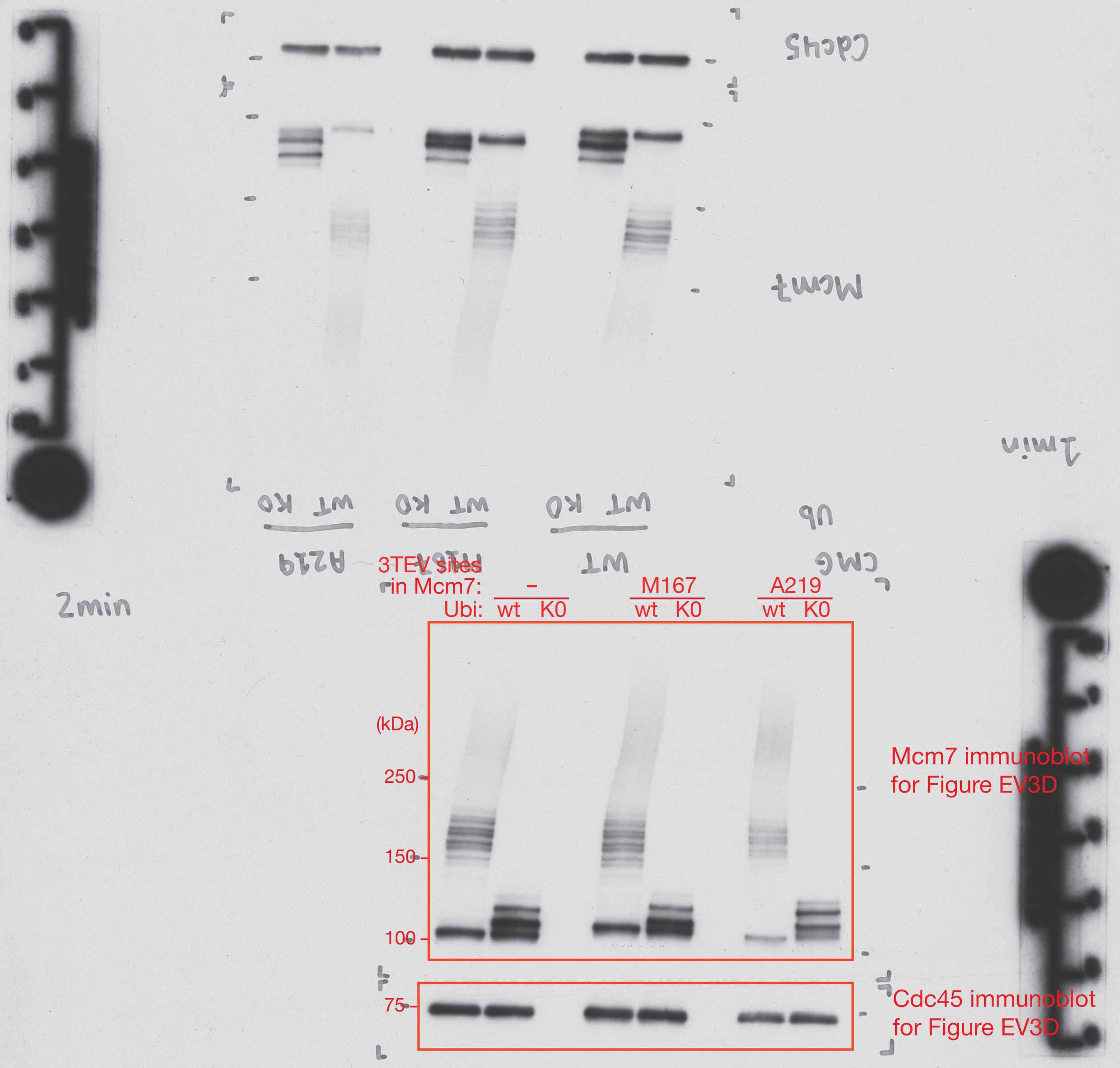
3TEV sites
in Mcm7:
–
M167
A219
Ubi:
wt
K0
wt
K0
wt
K0
(kDa)
250
150
100
75
Mcm7 immunoblot
for Figure EV3D
Cdc45 immunoblot
for Figure EV3D
2min
