# Supplementary material for: CMG helicase disassembly is essential and driven by two pathways in budding yeast
Source: EMBO J. 2024 Jul 22;43(18):2. doi: 10.1038/s44318-024-00161-x (PMC11405719; doi:10.1038/s44318-024-00161-x)
Supplement: Supplementary file 15 — EV Figure Source Data [file 44318_2024_161_MOESM15_ESM.zip › Source_Data_Figure_EV3.zip!/Source Data_Figure EV3/EV3C/Figure EV3C_Coomassie gel.pdf]

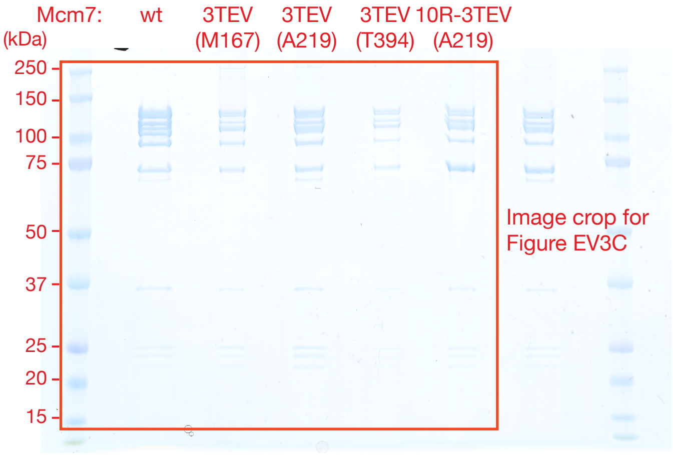
Mcm7:
(kDa)
wt
3TEV (M167)
3TEV (A219)
3TEV (T394)
10R-3TEV (A219)
250
150
100
75
50
37
25
20
15
Image crop for Figure EV3C
