# Supplementary material for: CMG helicase disassembly is essential and driven by two pathways in budding yeast
Source: EMBO J. 2024 Jul 22;43(18):2. doi: 10.1038/s44318-024-00161-x (PMC11405719; doi:10.1038/s44318-024-00161-x)
Supplement: Supplementary file 15 — EV Figure Source Data [file 44318_2024_161_MOESM15_ESM.zip › Source_Data_Figure_EV3.zip!/Source Data_Figure EV3/EV3B/Figure EV3B_Blots_Mcm7-Cdc45.pdf]

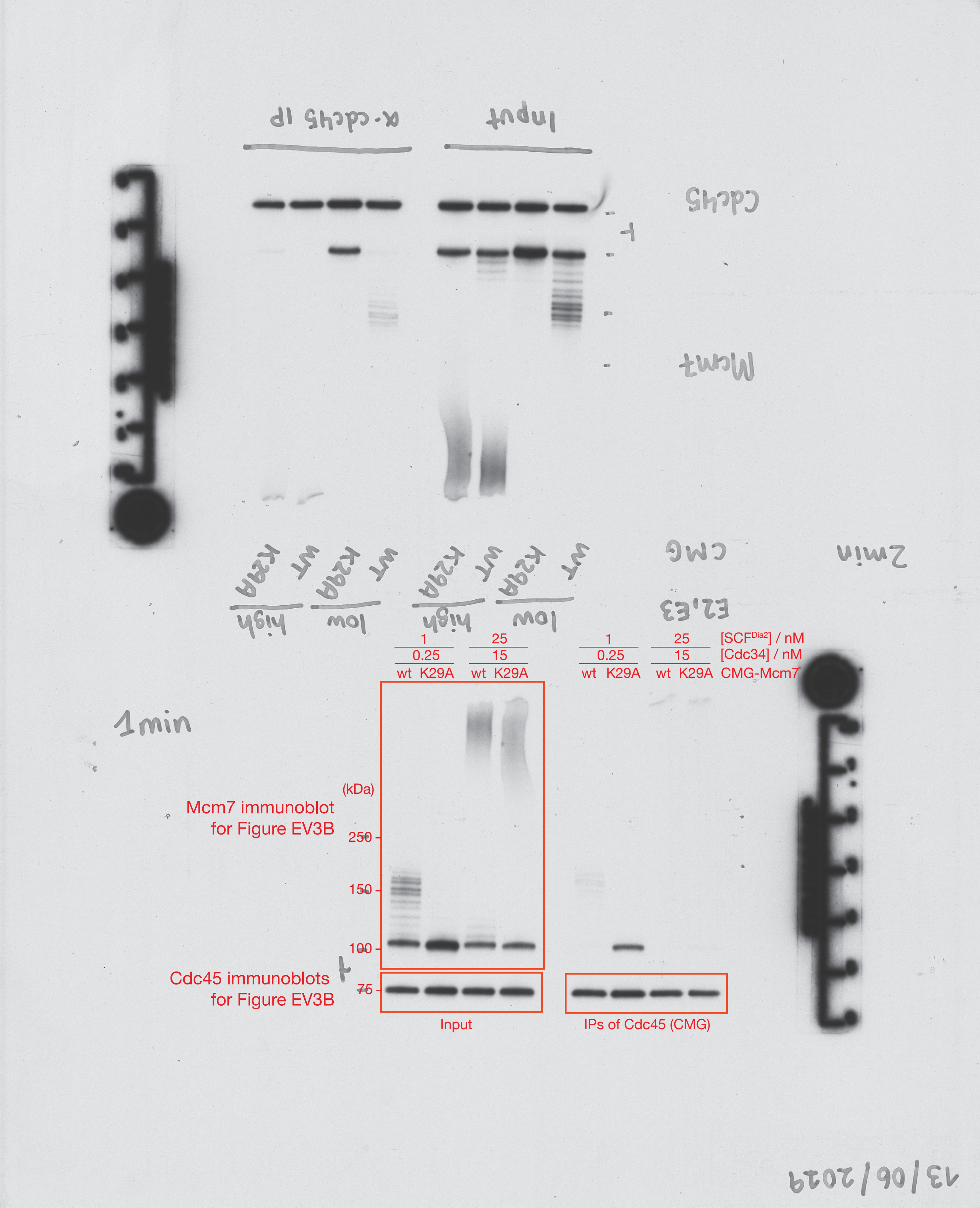

1
0.25
wt K29A
25
15
wt K29A
1
0.25
wt K29A
25
15
wt K29A
[SCFDia2] / nM
[Cdc34] / nM
CMG-Mcm7
1min
(kDa)
Mcm7 immunoblot
for Figure EV3B
250
150
100
75
Cdc45 immunoblots
for Figure EV3B
Input
IPs of Cdc45 (CMG)
