# Supplementary material for: CMG helicase disassembly is essential and driven by two pathways in budding yeast
Source: EMBO J. 2024 Jul 22;43(18):2. doi: 10.1038/s44318-024-00161-x (PMC11405719; doi:10.1038/s44318-024-00161-x)
Supplement: Supplementary file 15 — EV Figure Source Data [file 44318_2024_161_MOESM15_ESM.zip › Source_Data_Figure_EV4.zip!/Source Data_Figure EV4/EV4B/Figure EV4B_Plate image.pdf]

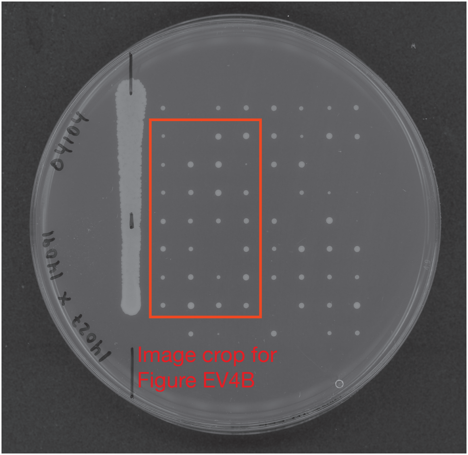
Image crop for
Figure EV4B
