# Supplementary material for: CMG helicase disassembly is essential and driven by two pathways in budding yeast
Source: EMBO J. 2024 Jul 22;43(18):2. doi: 10.1038/s44318-024-00161-x (PMC11405719; doi:10.1038/s44318-024-00161-x)
Supplement: Supplementary file 15 — EV Figure Source Data [file 44318_2024_161_MOESM15_ESM.zip › Source_Data_Figure_EV5.zip!/Source Data_Figure EV5/EV5H/Figure EV5H_Blots_Cdc45-Psf1-Psf2.pdf]

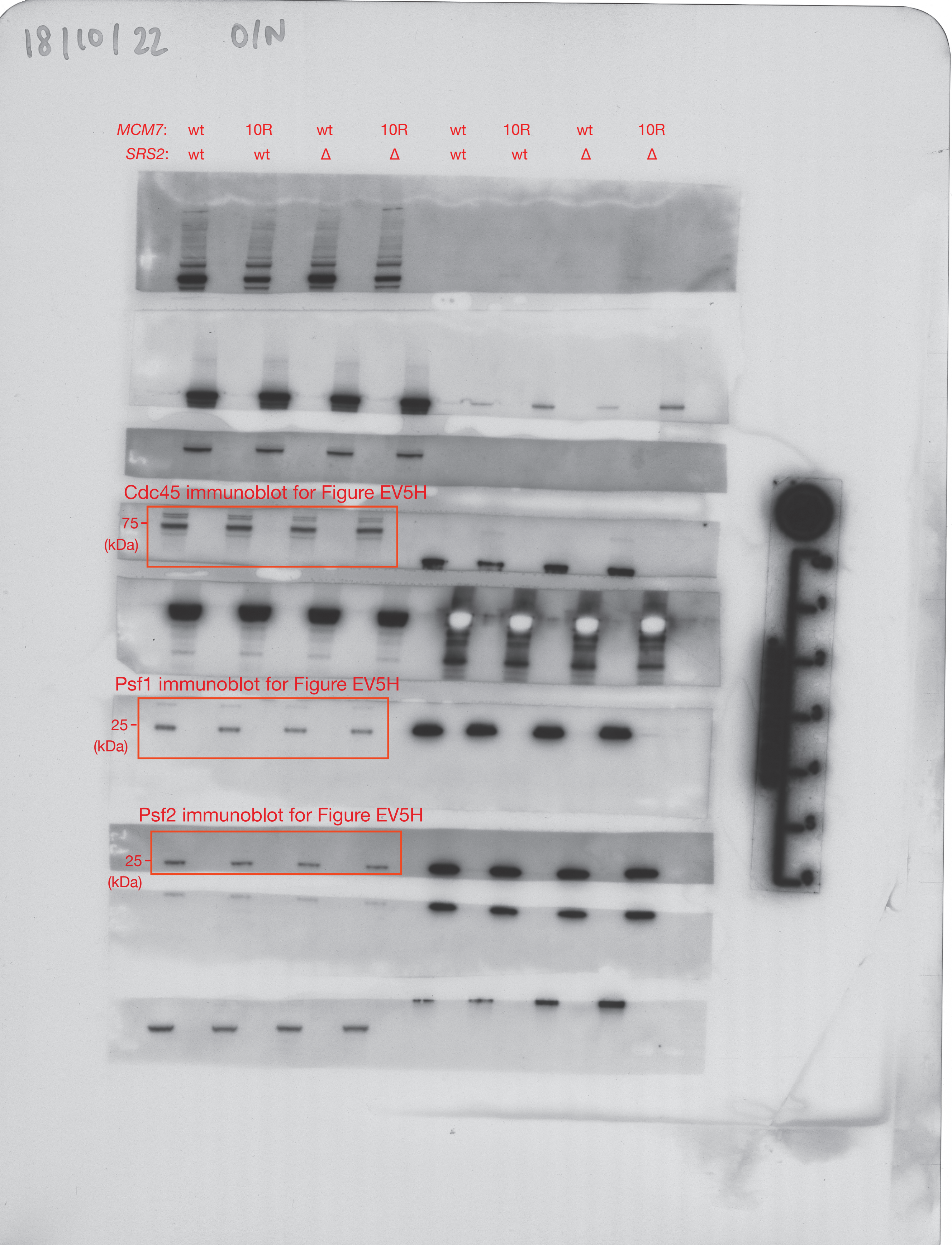
18/10/22 O/N
MCM7: wt 10R wt 10R wt 10R wt 10R
SRS2: wt wt Δ Δ wt wt Δ Δ
Cdc45 immunoblot for Figure EV5H
75
(kDa)
Psf1 immunoblot for Figure EV5H
25
(kDa)
Psf2 immunoblot for Figure EV5H
25
(kDa)
