# Supplementary material for: CMG helicase disassembly is essential and driven by two pathways in budding yeast
Source: EMBO J. 2024 Jul 22;43(18):2. doi: 10.1038/s44318-024-00161-x (PMC11405719; doi:10.1038/s44318-024-00161-x)
Supplement: Supplementary file 15 — EV Figure Source Data [file 44318_2024_161_MOESM15_ESM.zip › Source_Data_Figure_EV5.zip!/Source Data_Figure EV5/EV5H/Figure EV5H_Blots_Mcm7-Mcm6-Cdc45-Psf1-Psf2.pdf]

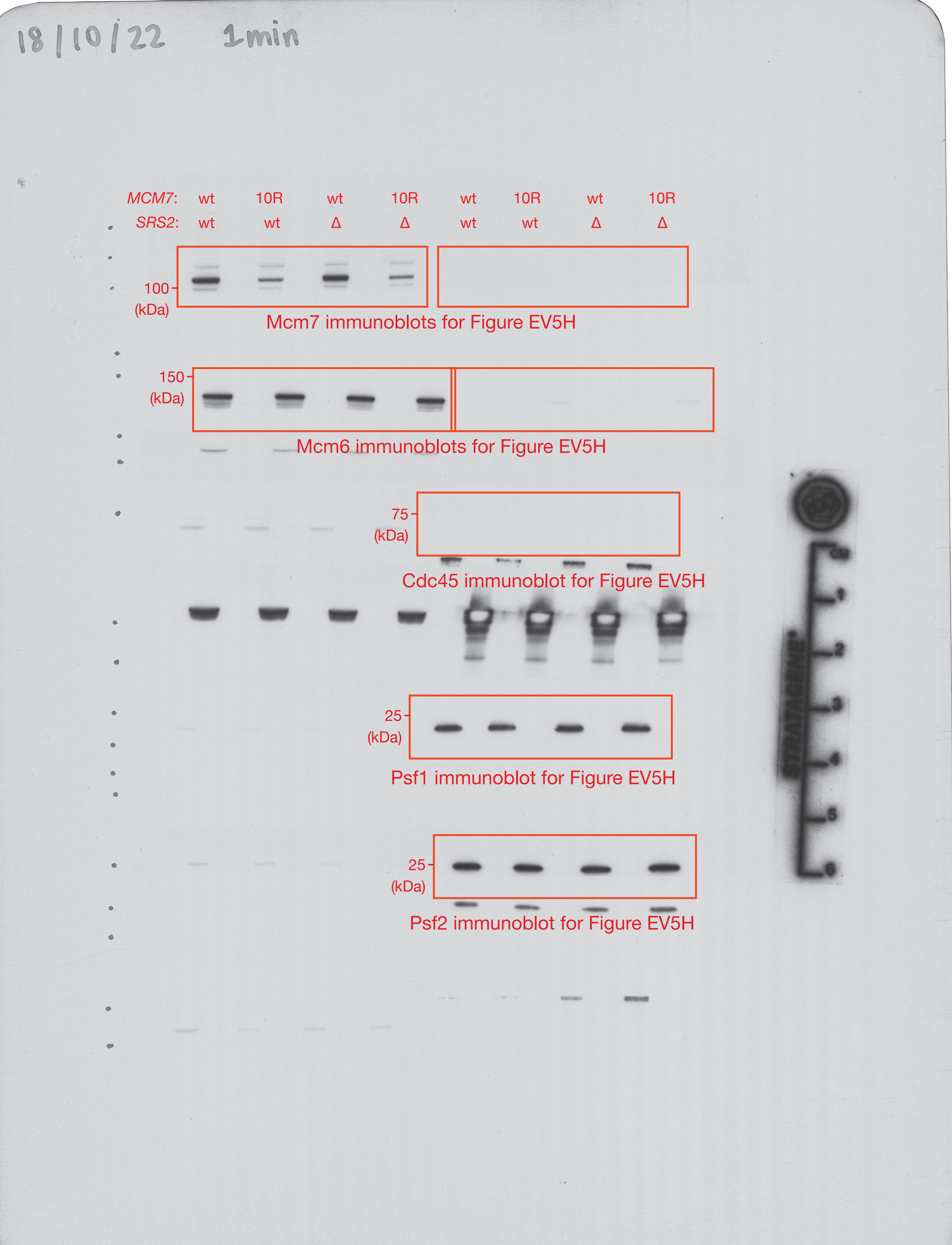
18/10/22
1min
MCM7: wt 10R wt 10R wt 10R wt 10R
SRS2: wt wt Δ Δ wt wt Δ Δ
100 (kDa)
Mcm7 immunoblots for Figure EV5H
150 (kDa)
Mcm6 immunoblots for Figure EV5H
75 (kDa)
Cdc45 immunoblot for Figure EV5H
25 (kDa)
Psf1 immunoblot for Figure EV5H
25 (kDa)
Psf2 immunoblot for Figure EV5H
STRATAGENE
CM
1
2
3
4
5
6
