# Supplementary material for: CMG helicase disassembly is essential and driven by two pathways in budding yeast
Source: EMBO J. 2024 Jul 22;43(18):2. doi: 10.1038/s44318-024-00161-x (PMC11405719; doi:10.1038/s44318-024-00161-x)
Supplement: Supplementary file 15 — EV Figure Source Data [file 44318_2024_161_MOESM15_ESM.zip › Source_Data_Figure_EV5.zip!/Source Data_Figure EV5/EV5F/Figure EV5F_Blot_Psf1.pdf]

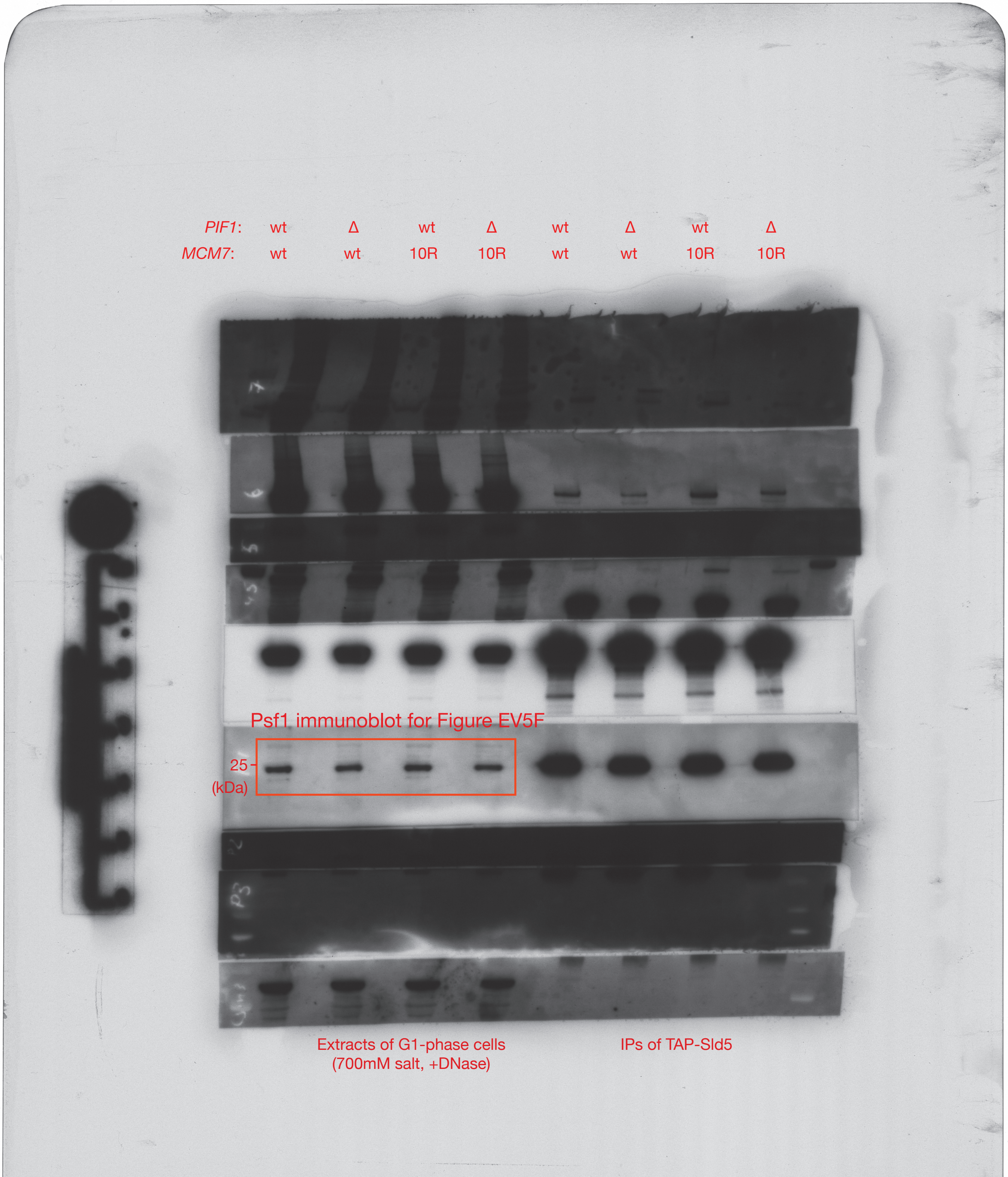
PIF1: wt Δ wt Δ wt Δ wt Δ
MCM7: wt wt 10R 10R wt wt 10R 10R
Psf1 immunoblot for Figure EV5F
25
(kDa)
Extracts of G1-phase cells
(700mM salt, +DNase)
IPs of TAP-Sld5
