# Supplementary material for: CMG helicase disassembly is essential and driven by two pathways in budding yeast
Source: EMBO J. 2024 Jul 22;43(18):2. doi: 10.1038/s44318-024-00161-x (PMC11405719; doi:10.1038/s44318-024-00161-x)
Supplement: Supplementary file 15 — EV Figure Source Data [file 44318_2024_161_MOESM15_ESM.zip › Source_Data_Figure_EV5.zip!/Source Data_Figure EV5/EV5F/Figure EV5F_Blots_Mcm7-Mcm6-Sld5-Psf1.pdf]

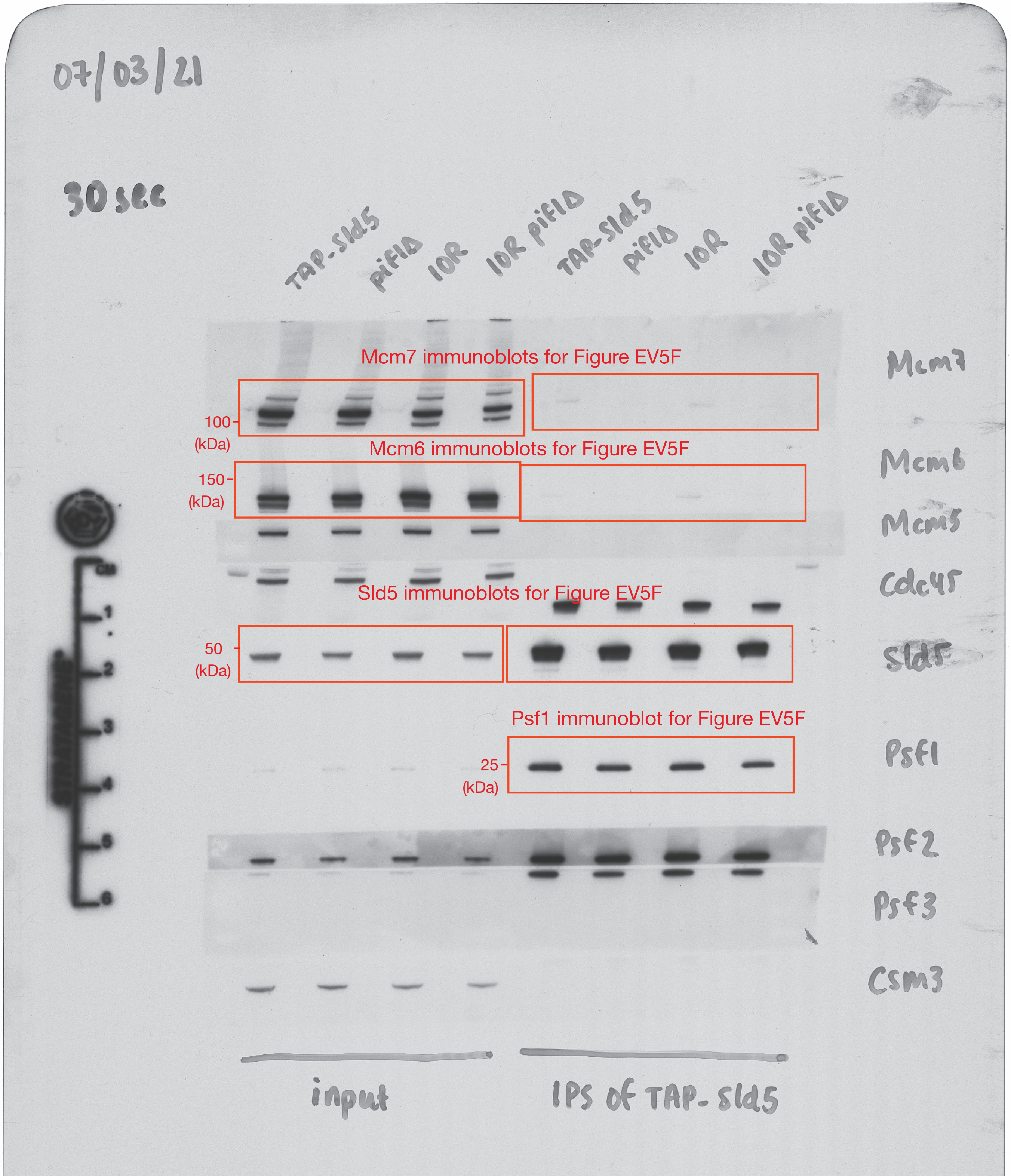
07/03/21
30 sec
TAP-Sld5
pif1Δ
10R
10R pif1Δ
TAP-Sld5
pif1Δ
10R
10R pif1Δ
Mcm7 immunoblots for Figure EV5F
100 (kDa)
Mcm6 immunoblots for Figure EV5F
150 (kDa)
Sld5 immunoblots for Figure EV5F
50 (kDa)
Psf1 immunoblot for Figure EV5F
25 (kDa)
Mcm7
Mcm6
Mcm5
Cdc45
Sld5
Psf1
Psf2
Psf3
Csm3
input
IPs of TAP-Sld5
