# Supplementary material for: CMG helicase disassembly is essential and driven by two pathways in budding yeast
Source: EMBO J. 2024 Jul 22;43(18):2. doi: 10.1038/s44318-024-00161-x (PMC11405719; doi:10.1038/s44318-024-00161-x)
Supplement: Supplementary file 15 — EV Figure Source Data [file 44318_2024_161_MOESM15_ESM.zip › Source_Data_Figure_EV5.zip!/Source Data_Figure EV5/EV5G/Figure EV5G_Blots_Mcm7-Mcm6.pdf]

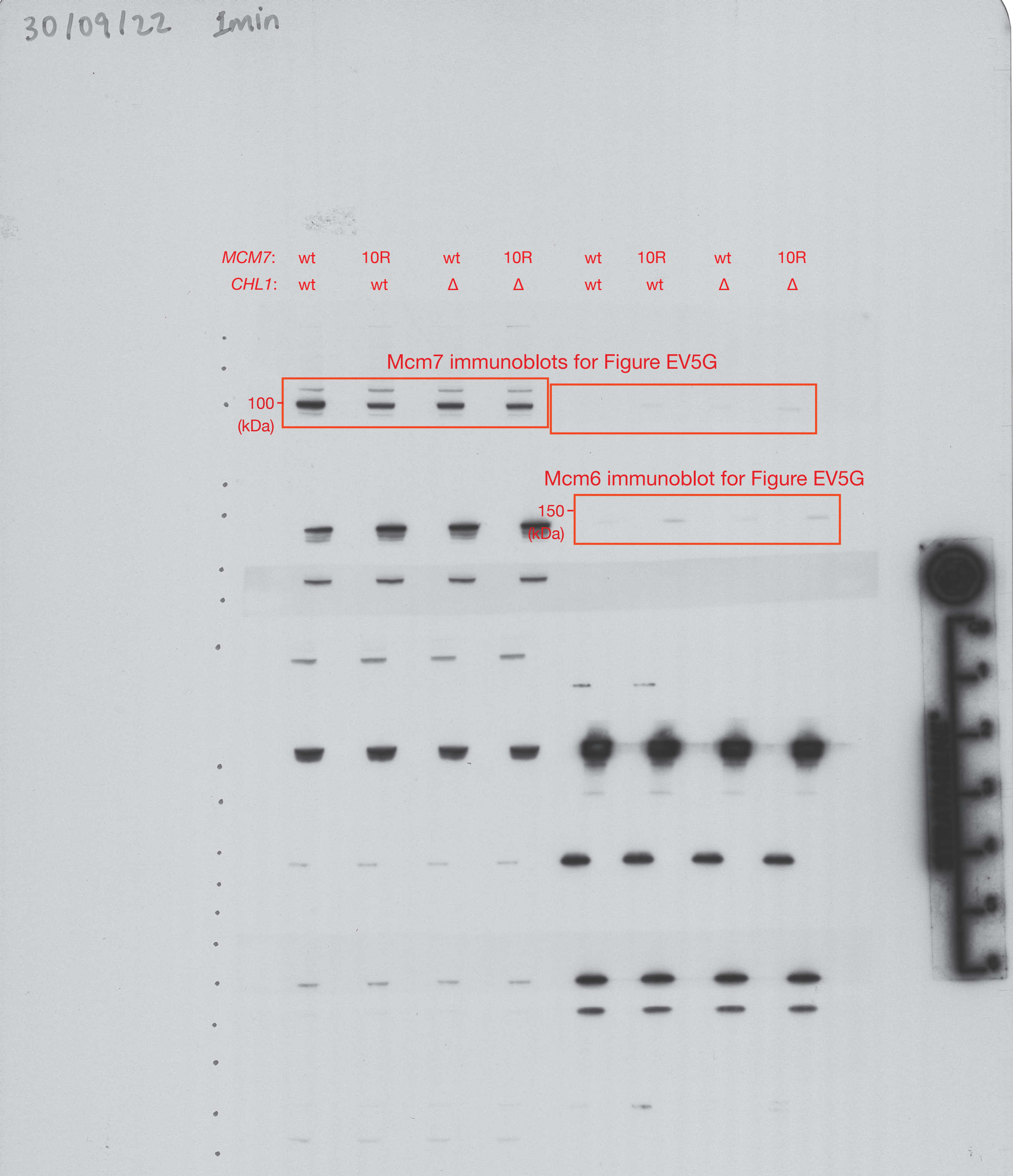

30/09/22 1min
MCM7: wt 10R wt 10R wt 10R wt 10R
CHL1: wt wt Δ Δ wt wt Δ Δ
Mcm7 immunoblots for Figure EV5G
100
(kDa)
Mcm6 immunoblot for Figure EV5G
150
(kDa)
