# Supplementary material for: CMG helicase disassembly is essential and driven by two pathways in budding yeast
Source: EMBO J. 2024 Jul 22;43(18):2. doi: 10.1038/s44318-024-00161-x (PMC11405719; doi:10.1038/s44318-024-00161-x)
Supplement: Supplementary file 15 — EV Figure Source Data [file 44318_2024_161_MOESM15_ESM.zip › Source_Data_Figure_EV5.zip!/Source Data_Figure EV5/EV5G/Figure EV5G_Blots_Cdc45-Psf2.pdf]

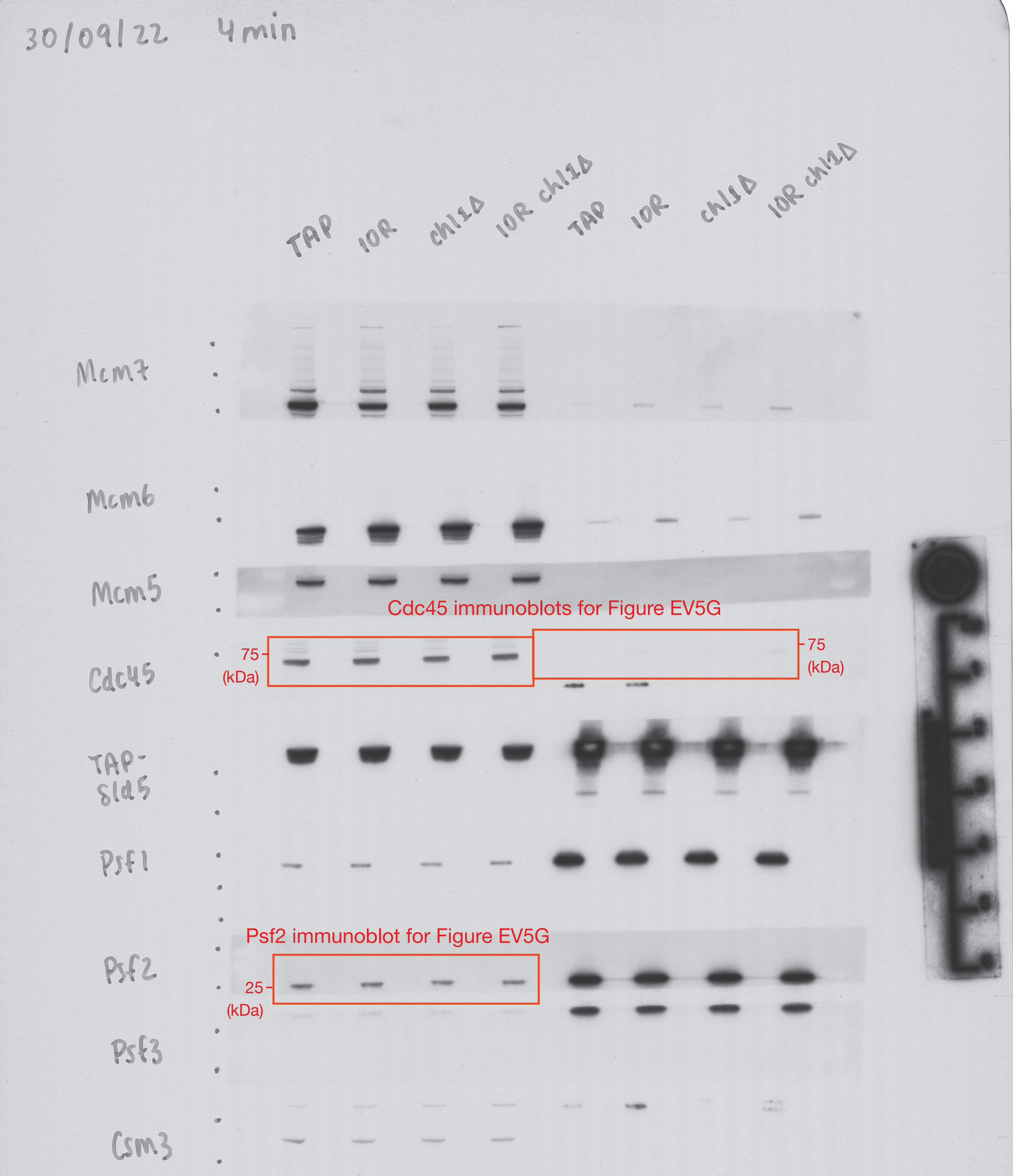
30/09/22 4min
TAP
10R
chl1Δ
10R chl1Δ
TAP
10R
chl1Δ
10R chl1Δ
Mcm7
Mcm6
Mcm5
Cdc45
TAP-Sld5
Psf1
Psf2
Psf3
Csm3
Cdc45 immunoblots for Figure EV5G
75 (kDa)
75 (kDa)
Psf2 immunoblot for Figure EV5G
25 (kDa)
